# Universal prediction of cell-cycle position using transfer learning

**DOI:** 10.1186/s13059-021-02581-y

**Published:** 2022-01-31

**Authors:** Shijie C. Zheng, Genevieve Stein-O’Brien, Jonathan J. Augustin, Jared Slosberg, Giovanni A. Carosso, Briana Winer, Gloria Shin, Hans T. Bjornsson, Loyal A. Goff, Kasper D. Hansen

**Affiliations:** 1 Department of Biostatistics, Johns Hopkins Bloomberg School of Public Health, Baltimore, USA; 2 Department of Genetic Medicine, Johns Hopkins School of Medicine, Baltimore, USA; 3 Department of Neuroscience, Johns Hopkins School of Medicine, Baltimore, USA; 4 Kavli Neurodiscovery Institute, Johns Hopkins University, Baltimore, USA; 5 Division of Biostatistics and Bioinformatics, Department of Oncology, Johns Hopkins School of Medicine, Baltimore, USA; 6 Department of Pediatrics, Johns Hopkins School of Medicine, Baltimore, USA; 7 Faculty of Medicine, Univeristy of Iceland, Reykjavik, Iceland; 8 Landspitali University Hospital, Reykjavik, Iceland

**Keywords:** Cell cycle, Single-cell RNA-sequencing, Transfer learning

## Abstract

**Background:**

The cell cycle is a highly conserved, continuous process which controls faithful replication and division of cells. Single-cell technologies have enabled increasingly precise measurements of the cell cycle both as a biological process of interest and as a possible confounding factor. Despite its importance and conservation, there is no universally applicable approach to infer position in the cell cycle with high-resolution from single-cell RNA-seq data.

**Results:**

Here, we present tricycle, an R/Bioconductor package, to address this challenge by leveraging key features of the biology of the cell cycle, the mathematical properties of principal component analysis of periodic functions, and the use of transfer learning. We estimate a cell-cycle embedding using a fixed reference dataset and project new data into this reference embedding, an approach that overcomes key limitations of learning a dataset-dependent embedding. Tricycle then predicts a cell-specific position in the cell cycle based on the data projection. The accuracy of tricycle compares favorably to gold-standard experimental assays, which generally require specialized measurements in specifically constructed in vitro systems. Using internal controls which are available for any dataset, we show that tricycle predictions generalize to datasets with multiple cell types, across tissues, species, and even sequencing assays.

**Conclusions:**

Tricycle generalizes across datasets and is highly scalable and applicable to atlas-level single-cell RNA-seq data.

**Supplementary Information:**

The online version contains supplementary material available at (10.1186/s13059-021-02581-y).

## Background

The cell cycle is the biological process which controls faithful replication and division of cells across all species of life. Despite existing as a continuous process, cell cycle has historically been characterized as having four discrete stages during which the cell performs growth and maintenance (G1), replicates its DNA (S), increases further in size and prepares for mitosis (G2), and undergoes mitosis and cytokinesis (M). Cell cycle is a highly conserved mechanism with an integral role in generating the diversity of cell types within multicellular organisms. As a result, maladaptive modifications of the cell cycle have devastating consequences in development and disease [1–3]. Despite its importance, many of the molecular mechanisms regulating and interacting with cell cycle remain poorly understood.

High-throughput expression data has been utilized for studying the cell cycle since the seminal work on the yeast cell cycle by Spellman et al. [4] and Cho et al. [5] at the dawn of the microarray era. This work used various approaches to synchronize cells in specific cell-cycle stages followed by assaying cells in bulk. The data from Spellman et al. [4] were later used by Alter et al. [6] to show that principal component analysis reveals a circular pattern which represents the cyclical nature of the cell cycle; widely cited as one of the first examples of the use of principal component analysis and singular value decomposition in analysis of high-throughput expression data. Subsequent work sought to systematically identify both periodically expressed genes and cell-cycle marker genes and deposited these into widely used databases [7, 8].

Single-cell technologies have enabled the ability to study the effects of cell cycle in multicellular organisms with a degree of sensitivity and accuracy only previously available in monocellular or clonal systems. Thus, cell cycle has been the subject of substantial interest, both as a biological variable of interest and as a possible confounding feature for other comparisons of interest [9]. A number of methods have been developed to estimate cell cycle state from single-cell expression data [10–15]; some of these approaches are related to more general methods for finding topological structure in single-cell data [16]. These methods differ broadly in the definition of cell-cycle state (discrete stages vs. continuous pseudotime) as well as the use of special training data. Most of these methods have been demonstrated to be effective on datasets consisting of a single cell type. Despite the conservation of the cell-cycle process, none of these methods have been shown to be applicable across single-cell technologies and mammalian tissues.

## Results

### Transfer learning

To develop a universal method for estimating a continuous cell-cycle pseudotime for a single-cell expression data set independent of technology, cell type, or species, we leverage transfer learning via dimensionality reduction [17, 18]. We define a reference cell-cycle embedding (or latent space) into which we project a new data set, an approach originally advocated for in Stein-O’Brien et al. [19]. After projection, we infer cell-cycle pseudotime as the polar angle around the origin. This pseudotime variable takes values in [0,2*π*] and is unrelated to wall time (time measured by a clock in SI units), but rather represents progression through the cell-cycle phases. We refer to this pseudotime variable as cell cycle position to avoid confusion with wall time and to emphasize its periodic nature.

To define a reference cell-cycle embedding, we leverage key features of principal component analysis of cell-cycle genes. Previous work has found that principal component analysis on expression data sometimes yields an ellipsoid pattern. This was first described by Alter et al. [6]; it has later been observed independently in multiple data sets [12, 15, 20]. Here, we draw attention to the fact that the ellipsoid pattern is a consequence of a link between Fourier analysis of periodic functions and principal component analysis which links the progression through the cell-cycle process with angular position on the ellipsoid. This is similar in spirit to previous observations of principal component analysis of genotype data [21] and connected to mathematical results on circulant matrices [22].

We use the first two principal components to define a reference embedding representing the cell cycle. Because this reference embedding is a low-dimensional linear space, we obtain an orthogonal projection operator allowing us to project any new data set into the reference embedding. We show that projecting new data into the reference cell-cycle embedding overcomes technical and biological challenges posed by data sets where substantial variation is explained by one or more factors different from cell cycle, such as cellular differentiation.

### Principal component analysis and periodic functions

**Fig. 1:**
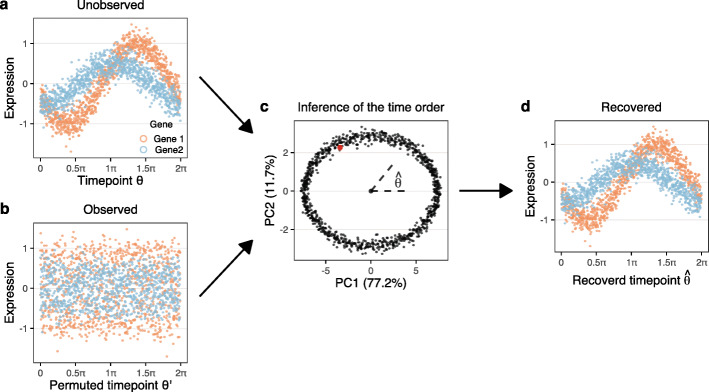
Principal component analysis recovers time ordering in simulations. Simulations are based on cosine functions with Gaussian noise (see the “Methods” section). (a) Expression vs. time for 2 genes with different peak locations and amplitudes. Each of the two gene peaks are replicated 50 times for a total of 500 genes and 1000 time points (cells). (b) Expression vs. permuted time, representing the unknown time order of observed data which obscures the periodicity of the functions. (c) Principal component analysis of the data from (b) and (a); the two datasets have equivalent principal components. We infer cell-cycle position ($\hat {\theta }$) by the angle of the ellipsoid. The red dot indicates *θ*=0. (d) Expression vs. inferred cell-cycle position

To gain insight into gene expression dynamics over the cell cycle, we start by analyzing principal component analysis of periodic functions. Our model is a collection of periodic functions with a single peak, taking the form

$$x_{g}(\theta) = A_{g} \cos(\theta - L_g) $$

with a gene-specific amplitude (*A*_*g*_) and location of the peak (*L*_*g*_) with 0≤*θ*<2*π* representing the unknown cell-cycle position. Figure 1 (a, b) depicts the unobserved (true) time ordering, observed on a discrete grid of time points, together with a random permutation of these time points; this represents the observed data which is not ordered by time. A key insight is the fact that the first two principal components are the same for the observed and the unobserved data (Fig. 1 (c)), when performed on a discrete set of observation times. The unknown time order can be inferred from the principal component plot as the angle of each point, making it possible to fully reconstruct the unobserved time order (Fig. 1 (d)), i.e., the first two principal components form an orthogonal projection into a two-dimensional space representing the periodic time. Our “Methods” section contains a constructive proof in the case of single peak genes, which are particularly relevant for cell-cycle expression.

The simulated data depicted in Fig. 1 has Gaussian noise, but we have verified that the result holds for data generated using the negative binomial distribution with an associated mean-variance relationship. In addition to using the negative binomial distribution, we also mimic the library size normalization step usually performed in scRNA-seq data analysis, which imposes a constraint on the total counts. With the total counts constraint, we need at least 3 distinct peak locations to be stable (Additional file 1: Figs. S1, S2). For both distributions, this approach is robust to downsampling of the data similar to what is seen with the increased sparsity from droplet-based sequencing technology. In simulations, we can recover cell-cycle position with as little as 10 total counts per cell across 100 genes (depending on noise levels and heights of the peaks) (Additional file 1: Fig. S3).

### Recovering cell-cycle position using principal component analysis on cell-cycle genes

We next show that PCA of cell-cycle genes form an ellipsoid as predicted by the cosine model presented in the previous section, and learn an embedding representing cell cycle. We use 10x Genomics Chromium single-cell RNA-sequencing (scRNA-seq) data on two replicate cultures of E14.5 mouse cortical neurospheres (see the “Methods” section), integrated using Seurat 3 and transformed to log2-scale. The use of an alignment method (CCA in Seurat3) to integrate the two samples is important for the quality of the ellipsoid, by maximizing the correlation structure between the two samples. Since neurospheres are maintained in a proliferative state, we expect that cell-cycle phase is an important contributor to the variation in expression within this single-cell dataset. To confirm this expectation, we consider a UMAP representation of the data based on all variable genes (Additional file 1: Fig. S4) colored according to the predictions from two separate cell-cycle stage estimation utilities (cyclone and a modification of Schwabe et al. [15] we call SchwabeCC, see the “Methods” section); this analysis demonstrates that the cell cycle is a major source of transcriptional variation in the neurosphere dataset.

**Fig. 2:**
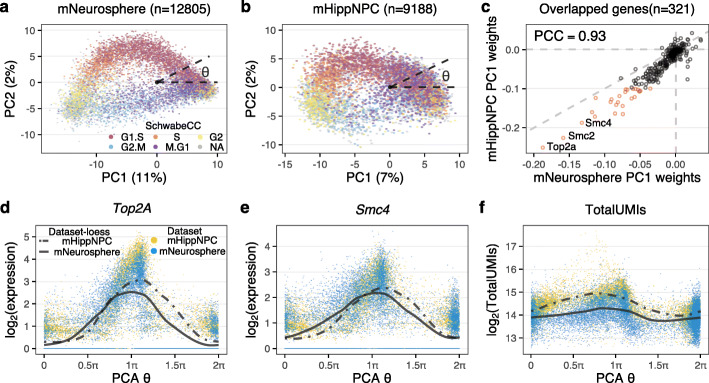
The cell-cycle ellipsoid and cell-cycle position. **a** Top 2 principal components of GO cell-cycle genes from E14.5 primary mouse cortical neurospheres, in which the variation is primarily driven by cell cycle. Each point represents a single cell, which is colored by 5-stage cell-cycle representation, inferred using the SchwabeCC method [15]. The cell-cycle position *θ* (with values in [0,2*π*); sometimes called cell-cycle pseudotime) is the polar angle. **b** As in (**a**), but for a dataset of primary mouse hippocampal progenitor cells from both a mouse model of Kabuki syndrome and a wildtype. **c** A comparison of the weights on principal component 1 between the cortical neurosphere and hippocampal progenitor datasets. Genes with high weights (|score|>0.1 for either vector) are highlighted in red. PCC: Pearson Correlation Coefficient. **d**, **e** The expression dynamics of **d***Top2A* and **e***Smc4* using the inferred cell-cycle position, with a periodic loess line (see the “Methods” section). **f** The dynamics of total UMI using the inferred cell-cycle position, with a periodic loess line, illustrating the high agreement of the dynamics between datasets

We then perform principal component analysis of the top 500 most variable genes amongst the roughly 1700 genes annotated with the Gene Ontology cell cycle term (*GO:0007049*; see the “Methods” section) [23]. The first two principal components form an ellipsoid with a sparse/empty interior (Fig. 2a); this reflects the behavior of our cosine model. Using the SchwabeCC cell-cycle stage predictor, we observe a strong relationship between polar angle on the ellipsoid and predicted cell-cycle stage.

The strong relationship between polar angle on the ellipsoid and predicted cell-cycle stage was also observed on an independent dataset on cultured primary mouse hippocampal progenitors from a wild-type mouse as well as from a *Kmt2d* ^+/*βgeo*^ mouse, a previously described model of Kabuki syndrome [24]. The data were processed similarly to the neurosphere data. Again, we select the top 500 most variable cell-cycle genes and perform a principal component analysis (Fig. 2b) which reveal an ellipsoid pattern. The shape of the principal component plot differs between the two datasets, but the weights used to form the first two principal components are highly concordant (Fig. 2c, Additional file 1: Fig. S5 for PC2) for the 321 genes present in both cell-cycle embeddings. Almost all of the highly ranked genes (absolute weights >0.1, highlighted in red and labeled with gene name) represent important regulators of, or participants in, the cell cycle. For example, the highest ranked gene is *Top2A* (Topoisomerase 2A) which controls the topological state of DNA strands and catalyzes the breaking and rejoining of DNA to relieve supercoiling tension during DNA replication and transcription [25]. Also highly ranked are *Smc2* and *Smc4* which compose the core subunits of condensin, which regulates chromosome assembly and segregation [26, 27].

Given our mathematical analysis as well as the strong empirical relationship between polar angle on the ellipsoid and cell-cycle stage predictions, we define a method to learn cell-cycle position as the polar angle around the origin on the coordinate plane which we denote by *θ*. We center the coordinate plane on (0,0). Because we mean-center the expression data prior to performing PCA, this location corresponds to having mean (across the dataset) expression for all 500 variable cell-cycle genes. Linking to our idealized example with periodic functions, this will correspond to a timepoint (cell) where all genes have 0 expression after mean centering.

To demonstrate that cell-cycle position reflects the true biological cell-cycle progression, we consider expression dynamics of specific cell-cycle genes. For *Top2A* and *Smc4* the peak expressions are observed at G2 stage around *π* (Fig. 2e), consistent with their known increased expression through S phase and into G2 [27–29]. Furthermore, the dynamics are highly similar between the independently analyzed cortical neurosphere and hippocampal NPC datasets, which supports the observation that the two different embeddings yield concordant cell-cycle positions (despite each including dataset-specific genes). These observations hold for all genes with high weights (Additional file 1: Fig. S6). This approach serves as an internal control in any single-cell RNA-seq data set and can be used to assess the quality of any continuous ordering.

Next, we directly relate estimated *θ* to the measured transcription values. Figure 2f shows the log2 transformed total UMI numbers against *θ*, with a periodic loess smoother for each dataset. In both datasets, the maximum level is reached around *π* and the minimum around 1.5*π*, which corresponds to the end of G2 and the middle of M stage respectively. We observe the total UMI number begins to increase at the beginning of G1/S phase and to decrease sharply as cells progress through M phase. The difference between the maximum and minimum of the periodic loess line is 1, corresponding to a twofold difference in total UMI, which is known to be proportional to cell size [30, 31]. This observation, and the timing with respect to cell-cycle position, is consistent with the approximate reduction in cellular volume by one half as a result of cytokinesis in M phase and the formation of two daughter cells of roughly equal size.

This approach of using expression dynamics and (if available) log2-totalUMI can be used to evaluate whether *any* continuous position is related to the cell cycle. We name these “internal controls” and we note that they are available for *any* single-cell expression dataset. These internal controls will be used extensively throughout this manuscript.

Note that these principal component analyses are differentiating G2/M cells from G1/G0 cells on the first principal component. This contrasts with the mathematical analysis where the starting point (*θ*=0) can be any location (red point in Fig. 1) as there is no clear starting point for a periodic function. That the first principal component differentiates G2M from G1/G0 can be explained by the nature of principal component analysis. Before principal component analysis, we subtract each gene’s mean expression. However, genes marking G2/M usually have very high expression compared to other stages, with G0/G1 being the lowest (Additional file 1: Fig. S7), ensuring that this becomes the first principal component. A clustering analysis of the expression patterns provides further evidence that cell-cycle genes have a single peak pattern of expression (Additional file 1: Fig. S7). Thus, the observed behavior of the cell-cycle genes in these data sets satisfy the assumptions of the cosine model.

In summary, principal component analysis of the cell-cycle genes predicts cell-cycle progression for the mNeurosphere and mHippNPC datasets with a high degree of similarity between the cell-cycle position inferred independently in the two datasets. This observed behavior aligns with the results from the cosine model.

### When principal component analysis fails to reflect cell-cycle position

**Fig. 3:**
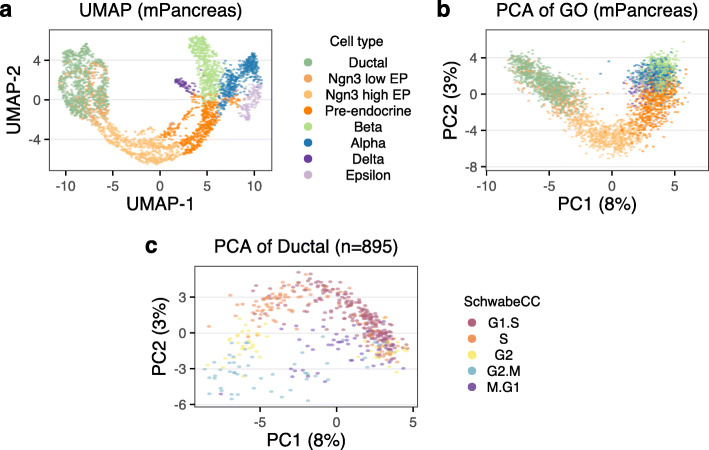
When principal component analysis fails to describe the cell cycle. Data is from the developing mouse pancreas. **a** UMAP embedding using all variable genes. Cells are colored by cell type. **b** PCA plot of the cell-cycle genes; this reflects the differentiation path in (**a**). **c** PCA plot of the cell-cycle genes for ductal cells only; this plot reflects cell cycle

A principal component analysis does not always yield an ellipsoid pattern; a requirement for this to work is for the first principal component to be dominated by cell cycle. To illustrate this, we used an existing mouse developing pancreas dataset, with cell type labels [32]. A major source of variation in this dataset is cellular differentiation as demonstrated by a standard UMAP embedding (based on all variable genes) illustrating the previously described [32] differentiation trajectories (Fig. 3a). When we perform principal component analysis using only the variable cell-cycle genes, the resulting PCA plot still reflects the differentiation trajectory and does not resemble the ellipsoid pattern observed in the previous section (Fig. 3b). Note that PC1 has some relationship with cell cycle since the differentiation path goes from cycling to non-cycling cells, but it also reflects the progression from cycling multipotent cells to terminally differentiated cells. This result strongly suggests that some of the cell-cycle genes may participate in biological processes other than the cell cycle and demonstrates that PCA of cell-cycle genes does not always exclusively capture cell-cycle variance.

However, when we perform principal component analysis only on a subset of cells from a single, proliferating progenitor cell type, the ellipsoid pattern returns (Fig. 3c and Additional file 1: Fig. S8a,b). This highlights the challenge of inferring cell cycle for datasets that contain many different cell types, including postmitotic cells.

### Transfer learning through projection

To overcome the challenges of inferring cell-cycle position in arbitrary datasets, we propose a simple, yet highly effective transfer learning approach we term tricycle (transferable representation and inference of cell cycle). In short, we first construct a reference embedding representing the cell-cycle process using a fixed dataset where cell cycle is the primary source of transcriptional variation. For the remainder of this manuscript, we will use the cortical neurosphere data as this reference, and we claim this is a useful reference regardless of dataset. We project new data into this embedding and infer cell-cycle position for each cell by the polar angle around the origin.

**Fig. 4:**
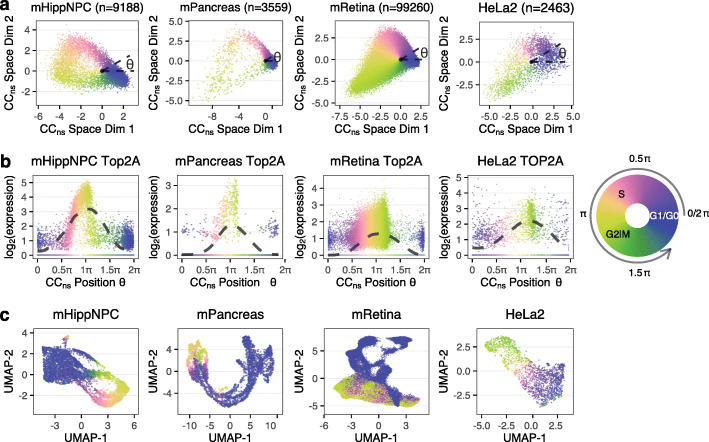
A pre-learned weights matrix learned from proliferating cortical neurospheres enables cell-cycle position estimation in other proliferating datasets. **a** Different datasets (hippocampal NPCs, mouse pancreas, mouse retina, and HeLa set 2) projected into the cell-cycle embedding defined by the cortical neurosphere dataset. Cell-cycle position *θ* is estimated as the polar angle. **b** Inferred expression dynamics of *Top2A* (*TOP2A* for human), with a periodic loess line (Methods). **c** UMAP embeddings of top variable genes. All the cells are colored by cell-cycle position using a circular color scale. We put the discrete stage labels in approximated position on the circular legend to help relate the continuous *θ* to the discrete stages

As a demonstration, we consider a diverse selection of single-cell RNA-seq datasets representing different species (mouse and human), cell types, and technologies (10x Chromium, SMARTer-Seq, Drop-seq, and Fluidigm C1) (Table 1). We project these datasets into the cell-cycle embedding learned from the neurosphere data (Fig. 4a, Additional file 1: Fig. S9a). To effectively visualize cell-cycle position defined as the polar angle, we use a circular color scale to account for the fact that position “wrap around” from 2*π* to 0. Although the shape of the projection varies from dataset to dataset, the cells of the same stage always appear at a similar position of *θ*, such as cells at S stage centering at 0.75*π*.

**Table 1:** Datasets

Dataset	Species	Platform	# cells	Note	Reference
mNeurosphere	Mouse	10x	12805		here
mHippNPC	Mouse	10x	9188		here
mPancreas	Mouse	10x	3559		[32]
mHSC	Mouse	SMARTer	1343		[33]
mRetina	Mouse	10x	99260		[34]
HeLa 1	Human	Drop-seq	1398		[15]
HeLa 2	Human	Drop-seq	2463		[15]
mESC	Mouse	Fluidigm C1	279	FACS	[9]
hESC	Human	Fluidigm C1	226	FACS	[10]
hU2OS	Human	SMART-seq2	1114	FUCCI	[20]
hiPSCs	Human	Fluidigm C1	888	FUCCI	[14]
Fetal tissue atlas	Human	sci-RNA-seq3	Varies		[35]

To verify our cell-cycle ordering, we again use the internal controls as they exist in all datasets. Specifically, we show the expression dynamics of *Top2A* and *Smc4* as a function of *θ* (Fig. 4b, Additional file 1: Figs. S9b, S10). In contrast, PCA plots of the GO cell-cycle genes for each dataset illustrates the advantage of using a fixed embedding to represent cell cycle (Additional file 1: Fig. S11). Together, these results strongly support that tricycle generalizes across data modalities.

Having inferred cell-cycle position, we can visualize the cell-cycle dynamics on a UMAP plot representing the full transcriptional variation, as is standard in the scRNA-seq literature (Fig. 4c, Additional file 1: Fig. S9c). Doing so reveals the smooth behaviour of the tricycle predictions (despite not using smoothing or imputation) and argues for representing cell cycle in gene expression data as a continual progression rather than discrete states.

We draw attention to two specific datasets in these figures: the mPancreas and mRetina datasets (Fig. 4) and the HCA (human cell atlas) Pancreas data (Additional file 1: Fig. S9). These three datasets contain many different cell types and strong drivers of gene expression in addition to the cell cycle, such as differentiation. We have previously seen how principal component analysis fails to be ellipsoid on the mPancreas dataset (Fig. 3) and this example shows how tricycle—by projecting the data into a fixed reference embedding—overcomes the limitations of principal component analysis (Additional file 1: Fig. S12). Finally, note that the HCA Pancreas dataset is sparse with a median of 892 total UMIs per cell.

### Cell-cycle position estimation on gold-standard datasets

We validated tricycle on multiple datasets containing “gold-standard” cell-cycle measurements, including measurements by proxy using the fluorescent ubiquitination-based cell-cycle indicator (FUCCI) system and by fluorescence-activated cell sorting (FACS) of cells into discrete cell-cycle stages. Both of these approaches allow for assignment to or selection of cells from discrete phases of the cell cycle. The FUCCI system uses a dual reporter assay in which the reporters are fused to two genes with dynamic and opposing regulation during the cell cycle [36], allowing for a quantitative assessment of whether cells are in G1 or S/G2/M phase. In contrast to FACS, FUCCI systems, combined with an appropriate quantification method, make it possible to continuously measure cell cycle progression by placing the 2 protein measurements in a 2-dimensional space. Cell-cycle pseudotime needs to be inferred from these 2-dimensional measurements, which is usually done by a variant of polar angle [14, 20].

**Fig. 5:**
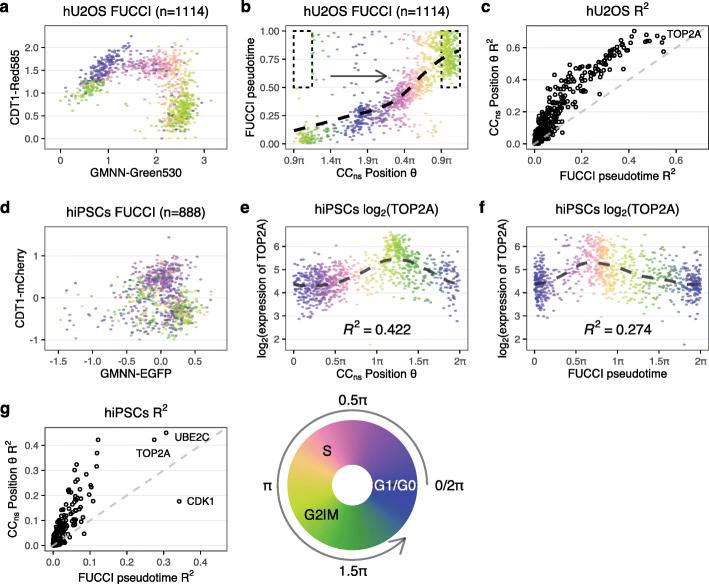
Evaluation of tricycle on FUCCI datasets. **a**–**c** Data from Mahdessian et al. [20]. **a** FUCCI scores colored by tricycle cell-cycle position. **b** Comparison between FUCCI pseudotime and tricycle cell-cycle position with a periodic loess line. We are displaying the data on [0.9*π*,0.9*π*+2*π*] (compared to [0,2*π*] elsewhere) because FUCCI pseudotime of 0 roughly corresponds to a tricycle position of 0.9*π*. Cells in the dotted rectangle were moved for display purposes in this panel by adding 2*π* (one period) to tricycle *θ* to reflect the higher temporal resolution around the anaphase-metaphase transition for FUCCI pseudotime (see the “Results” section). **c** *R*^2^ values of periodic loess line of all projection genes when using tricycle *θ* and FUCCI pseudotime as the predictor. The dashed line represents *y*=*x*. **d**–**g** Data from Hsiao et al. [14]. **d** FUCCI scores colored by tricycle cell-cycle position. **e**, **f** Expression dynamics of *Top2A* with a periodic loess line using either **e** tricycle cell-cycle position or **f** FUCCI pseudotime inferred by Hsiao et al. [14]. Cells are colored by corresponding *x*-axis values. **g** Similar to (**c**), but for the data from Hsiao et al. [14]

Mahdessian et al. [20] measured human U-2 OS cells to derive a FUCCI-based pseudo-time scoring. Their FUCCI measurements form a distinct horseshoe shape with the left side of the horseshoe representing time post-metaphase-anaphase transition with a continuous progression through G1, S, G2 and ending pre-metaphase-anaphase transition (Fig. 5; this depiction mirrors other data presentations [36, 37]). Cell cycle is a continuous process which is not immediately reflected in the horseshoe form because of the large gap (in the x-axis) between the two ends of the horsehoe. The *x*-axis reflects the protein levels of geminin (GMNN) which is degraded during the metaphase-anaphase transition [38] and the two “open” ends of the horseshoe are closely connected in time despite the visual gap in the scatterplot. This fact gives the FUCCI system the ability to assess whether a cell in M phase is before or after this transition, or said differently, a high temporal resolution around this transition despite the relatively short wall time compared to the rest of the cell cycle. We observe a close correspondence between tricycle cell-cycle position and FUCCI pseudotime (circular correlation coefficient *ρ*=0.70). The only cells for which there is a apparent disagreement are placed in M phase by tricycle (cell-cycle position around 0.9*π*) and are split between pre-metaphase-anaphase transition and post-metaphase-anaphase transition by FUCCI pseudotime, for this particular transition the FUCCI system has higher temporal resolution than tricycle; adding a small offset to these cells results in a remarkable concordance between the two systems (Fig. 5). Elsewhere in the cell cycle, there is no evidence of better temporal resolution with FUCCI; examining expression dynamics suggests that tricycle does at least as well as FUCCI at ordering key cell-cycle genes. We can use tricycle to examine the expression dynamics of *GMNN* and *CDT1* which reveals that *GMNN* expression is stable across the cell cycle (Additional file 1: Fig. S13), suggesting the protein is predominantly regulated post-transcriptionally during mitosis.

Hsiao et al. [14] used FUCCI on human induced pluripotent stem cells (iPSC) followed by scRNA sequencing using Fluidigm C1. While the Mahdessian et al. [20] FUCCI data look like a horseshoe, the Hsiao et al. [14] FUCCI data are more akin to a cloud (the data differ in quantification and normalization of the FUCCI scores). These data are used to estimate a continuous cell-cycle position (which we term “FUCCI pseudotime”) based on polar angle of the FUCCI scores. Compared with the data in Mahdessian et al. [20], there are larger differences between FUCCI pseudotime and tricycle cell-cycle position. However, we can directly compare the associated expression dynamics of key cell-cycle genes (Fig. 5 for *TOP2A*, Additional file 1: Fig. S14 for 8 additional genes). These results suggest that tricycle cell-cycle position is at least as good or better as the FUCCI pseudotime at ordering the cells along the cell cycle; the *R*^2^ for *TOP2A* is 0.42 for tricycle compared with 0.27 for peco.

In contrasts to FUCCI measurements, FACS sorting and enrichment of cells yields groups of genes in (supposedly) distinct phases of the cell cycle. We consider 2 different datasets where FACS has been combined with single-cell RNA-seq. Buettner et al. [9] assays mouse embryonic stem cells (mESC) using Hoechst 33342-staining followed by cell isolation using the Fluidigm C1. They use very conservative gating for G1 and G2M at the cost of less conservative gating for S phase. Leng et al. [10] uses FACS on FUCCI-labeled H1 human embryonic stem cells (hESC) followed by cell isolation using the Fluidigm C1. In both experiments, cells largely appear as expected in the cell-cycle embedding defined by the cortical neurosphere reference embedding (Additional file 1: Fig. S15). For the mESC, we note that some cells labeled S (but not G1 or G2M) appear outside the position expected for this stage, consistent with the gating strategy used for these data.

Summarizing this evidence, we conclude that tricycle recapitulates and refines the cell cycle ordering consistent with current “state of the art” experimental methods. Tricycle cell-cycle position is competitive with FUCCI-based measurements, except for cells in the metaphase to anaphase transition during mitosis.

### Comparison with existing tools for cell-cycle position inference

We next sought to compare tricycle cell-cycle position estimates with those obtained from other available methods. Existing methods for cell-cycle assessment can be divided into those which infer a continuous position and those which assign a discrete stage. We have evaluated the following methods: peco [14], Revelio [15], Oscope [10], reCAT [12], cyclone [11], Seurat [13], the original Schwabe [15], and the SchwabeCC 5 stage assignment method. Each method differs in which datasets it works well on and which issues it might have; a detailed comparison is available in the Supplementary Materials (Additional file 1: Supplementary Methods, and Figs. S16-S22).

Issues with existing methods include (a) the ability to work on datasets with multiple cell types, (b) the ability to scale to tens of thousands of cells or more, and (c) the ability to work on less information-rich datasets such as those generated by droplet-based or *in situ* scRNA-seq methods. Oscope requires data on many genes due to its use of pairwise correlations and therefore does not work on less information rich platforms (e.g. 10x Chromium or Drop-Seq). peco works better on less sparse and information-rich data (e.g. Fluidigm C1), but even on data from this platform, it is outperformed by tricycle. reCAT is critically dependent on the extent to which a principal component analysis of the cell-cycle genes reflects cell cycle and only infers a cell ordering; it is not straightforward to interpret the reCAT ordering, especially across datasets. Revelio is primarily a visualization tool, which appears to fail on datasets where substantial variation is driven by processes other than the cell cycle. Of the discrete predictors, Seurat agrees well with tricycle (and is very scalable) but is limited by only predicting a 3-stage cell-cycle representation (G1/S/G2M). Cyclone appears to do poorly in labeling cells in S phase and only predicts 3 stages. The SchwabeCC predictor assigns 5 stages, but has many missing labels and mis-assigns cells from G0/G1 to other stages.

Additionally, we benchmarked the computational speed and performance of tricycle against other cell-cycle estimation algorithms. We briefly compared the running time of several methods using subsets of the mRetina dataset (Additional file 1: Fig. S23). To compute continuous estimates using tricycle takes a mean of about 0.58, 0.86, and 1.48 s when the number of cells is 5000, 10,000, and 50,000 respectively. In contrast, to compute finite discrete stages, Seurat takes a mean of about 1.10, 1.22, and 4.95 s for a three-stage estimation and cyclone takes a mean of about 7.96, 11.50, and 50.66 min for a three-stage estimation, when the number of cells is 5000, 10,000, and 50,000 respectively. Other methods (peco, Oscope, reCAT) are not capable of processing large (10k–100k+) datasets. All of the comparisons were run on Apple Mac mini (2018) with 3.2 GHz 6-Core Intel Core i7 CPU, 64GB RAM, and operating system macOS 11.2. Thus, tricycle is able to scale with the increasing size of datasets.

### Application of tricycle to a single-cell RNA-seq atlas

**Fig. 6:**
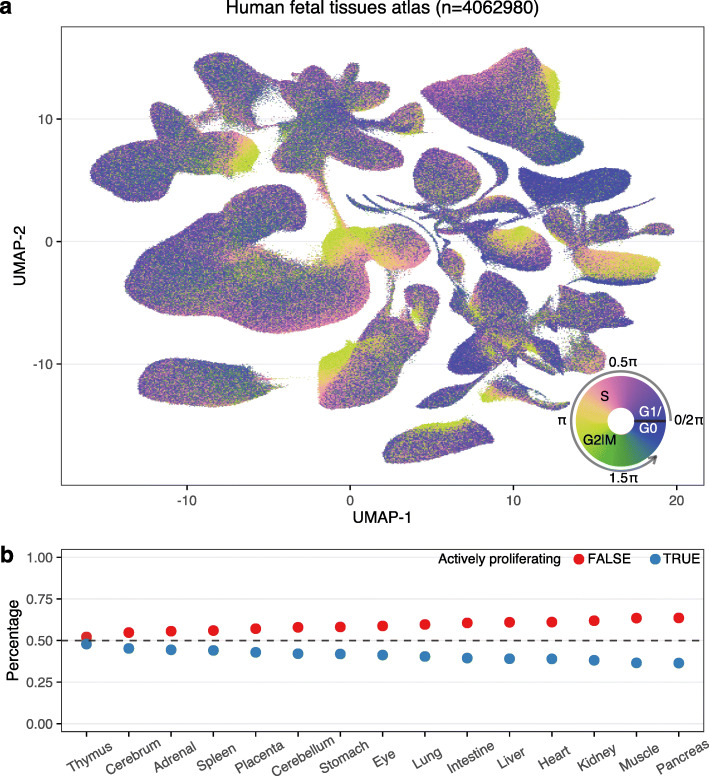
Application of tricycle on a human fetal tissue atlas. Data is from Cao et al. [35]. **a** UMAP embedding of human fetal tissue atlas data colored by cell-cycle position *θ* estimated using mNeurosphere reference. **b** The percentage of actively proliferating cells in human fetal tissue atlas. Tissues are ordered decreasingly with the percentage. Tissue and cell type annotations are available in Additional file 1: Fig. S25

To demonstrate the scalability and generalizability of tricycle we applied it to a recent dataset of ≈4 million cells from the developing human [35]. The data were generated using combinatorial indexing (sci-RNA-seq3) and are relatively lightly sequenced with a median of 429−892 total UMIs for 4 single-cell profiled tissues and 354−795 for 11 single-nuclei profiled tissues (Additional file 1: Fig. S24). Using tricycle, we are able to rapidly and robustly annotate cell-cycle position for each of the cells/nuclei in this atlas (Fig. 6a, Additional file 1: Fig. S25). Within a global UMAP embedding, tricycle annotations enable immediate visual identification of proliferating and/or progenitor cell populations for most cell types and tissues. The rapid annotation of cell-cycle position on this reference dataset further allowed us to examine the relative differences in the proportion of cells actively proliferating across different tissues and cell types in the developing human. To quantify this, we discretized all cells along *θ* into two bins corresponding to actively proliferating (0.25*π*<*θ*<1.5*π*; S/G2/M) or non-proliferating (G1/G0). We next ranked each tissue by the relative proportion of actively proliferating cells to identify the tissues and cell types with the highest proliferative index (Fig. 6b). To examine cell-type–specific differences in proliferation potential, we computed the cell-cycle embedding as well as the proliferative index for the 9 most abundant cell types within each tissue (Additional file 1: Figs. S26, S27).

Tissue-level proliferation indexes identified the thymus, cerebrum, and adrenal gland as having the highest overall proportions of dividing cells across the sampled fetal timepoints. Within the thymus, thymocytes represent both the most abundant cell type and the most “prolific” cell types as a function of the proportion of mitotic cells. Thymocytes exhibit a circular embedding in UMAP space that effectively recapitulates the estimated cell-cycle position predictions from tricycle (Additional file 1: Fig. S27k). Within this circular embedding, there is a gap of cells with cell-cycle position estimates at ≈*π*, consistent with dropout of cells and lower information content in M-phase. Comparison of tricycle cell-cycle annotations to SchwabeCC cell-cycle phase calls in this embedding suggests that tricycle more accurately estimates cell-cycle position even on cell types with a mean total UMI of 354 (Additional file 1: Fig. S28).

Within tissues, lymphoid cells are often the cell type with the highest proliferation index (Additional file 1: Figs. S26, S27), often with a greater number of actively proliferating cells than not. Within the fetal liver and spleen — both sites of early embryonic erythropoiesis during human development [39] — erythroblasts represent the cell type with the highest fraction of proliferating cells. Across developmental time, most tissues maintain relatively monotonic proliferation indices; however, several (liver, placenta, intestine) exhibit dynamic changes across the sampled timepoints. This application illustrates the utility of tricycle to atlas-level data.

### Stability of the cell-cycle position assignments

To test the robustness of tricycle, we performed in silico experiments to determine the stability of cell-cycle position assignments. We evaluated three different types of stability wrt: (a) missing genes, (b) sequencing depth, and (c) data preprocessing.

When projecting new data into the cell-cycle reference embedding, it is common that the feature mapping between the two data sets contains only a subset of the 500 genes used in the embedding. The number of genes available for feature mapping has an impact on the shape of the resulting embedding; the mNeurosphere and mHippNPC datasets have almost the same shape when restricted to a set of common genes (Additional file 1: Fig. S29). To establish the stability of tricycle, we randomly removed genes from the neurosphere dataset and computed tricycle cell-cycle positions; we used the neurosphere dataset as a positive control to ensure all genes are present. We used the circular correlation coefficient to assess the similarity between the tricycle cell-cycle position for the full dataset vs. the dataset with randomly pruned genes (Additional file 1: Figs. S30, S31). This reveals excellent stability (circular *ρ*>0.8) using as little as 100 genes.

To examine the impact of sequencing depth, we downsampled the mHippNPC dataset (Additional file 1: Figs. S32, S33) and used the circular correlation coefficient to quantify the similarity to the cell-cycle position inferred using the full sample. Originally, the median of library sizes (total UMIs) is 10,000 for mHippNPC data. Downsampling to 20% of the original depth(approximate median of library sizes 2000) kept circular *ρ*>0.8. This is congruent with the observed robustness of the method to the varying sequencing depth of the various datasets examined above.

Next, we examined the stability of tricycle regarding the choice of reference embedding. Above, we show a cell-cycle space estimated separately for the mNeurosphere and the mHippNPC datasets (Fig. 2). We observe that the inferred expression dynamics are more alike in the two datasets if we project the mHippNPC into the mNeurosphere embedding compare to using its own embedding. To quantify this, we pick key cell-cycle genes (previously examined in Additional file 1: Fig. S6) and compare the location of peak expression in the mNeurosphere dataset to that of the mHippNPC dataset with cell-cycle position estimated using these two approaches (Additional file 1: Fig. S34). For the vast majority of genes, the highest expression appear at a closer position when we estimate cell-cycle position by projecting the mHippNPC dataset into the mNeurosphere embedding.

To examine the impact of preprocessing data prior to projection, we compared cell-cycle position inferred using data processed with and without Seurat. Note that when we estimate the cell-cycle space, we use Seurat to align the different biological samples. But this is not done when we project new data using the prelearned reference. We observe negligible differences, whether or not Seurat is used (Additional file 1: Fig. S35). We also confirmed the direction of cell-cycle position *θ* is consistent with the direction of RNA velocity projections (Additional file 1: Fig. S36).

These results demonstrate the high sensitivity of tricycle to accurately estimate the cell cycle position across a high dynamic range of number of detectable genes within the feature map as well as depth of the information content in the target cells.

## Discussion

Here, we have demonstrated the ability of tricycle to infer cell-cycle position in 26 datasets across species, cell types, and assay technologies. To do so — as is common in the field — we have made extensive use of gold standard datasets, with a particular emphasis on the FUCCI assay. We show tricycle compares favorably to FUCCI-based pseudotime, specifically the tricycle inferred cell-cycle position is a better predictor of expression dynamics of key cell-cycle genes compared to FUCCI-based pseudotime; however, FUCCI pseudotime has higher temporal resolution during the metaphase to anaphase transition, a very specific point during cell division.

An important limitation of existing gold standard datasets is that the measurements are done on cell lines where the main driver of expression can be assumed to be cell cycle. In contrast, many common applications of single-cell expression contain multiple cell types (e.g., tissue samples) and/or other strong drivers of expression such as differentiation. Predicting cell-cycle position in such datasets is much harder than predicting cell-cycle position on homogeneous cell lines. For this reason, it is not enough to merely assess a method on gold standard datasets.

To address the limitation of gold-standard measurements on cell lines, we have made extensive use of internal controls. Specifically, we use inferred cell-cycle position to assess whether key cell-cycle genes (and log totalUMI if available) exhibit the expected expression dynamics across the learned *θ* progression. These internal controls are available in *any* single-cell expression dataset, including complex tissue samples. These internal controls do not by themselves give a clear answer to how precise the predictions are, but they undoubtedly carry some information on whether the inferred cell-cycle position is at all associated with cell-cycle phases. By using these internal controls, we overcome the limitations of the available gold standard datasets — which we can think of as having “external controls” — and show that tricycle performs well on differentiation datasets and datasets with multiple cell types. Additionally, we are able to ascertain the generalizability of the method. The cell-cycle genes we use for evaluation are also used to construct the reference embedding and for projection. However, at the projection stage, the weights are fixed without any dataset-dependent optimization. This removes — in our opinion — any circular reasoning. We note that internal controls are useful to assess any continuous prediction of cell-cycle position.

Here, we use a fixed reference embedding to represent cell cycle, defined using the mouse cortical neurosphere dataset. This raises the question: is there a single best embedding? One part of this question is whether there is a minimal best set of genes to construct the embedding? Empirically, when we project the neurosphere data into itself and remove genes, we get good performance with around 100 genes. However, this experiment does not measure generalizability, and we have anecdotally observed that reducing our gene list this much impacts performance in some datasets. Another part of this question is whether we can optimize the embedding to be as circular as possible. We observe that, despite different shapes, embeddings based on the cortical neurosphere and the primary hippocampal NPC datasets result in similar cell-cycle position estimates. One interpretation of these observations is that the robustness of the approach is derived from the structure created by the relationship of the genes to each other rather than the behavior of any individual marker gene, as described in Stein-O’Brien et al. [19].

In many single-cell experiments, cell cycle is often considered a confounding factor and as such, methods exist to remove this effect from the data prior to analysis. We caution against *removing* cell-cycle progression blindly as it can be intimately intertwined with other sources of variation of interest. Taking the mPancreas data as an example, there is a clear relationship between the number of cycling cells and differentiation as the multi-potent ductal cells advance to be terminally differentiated alpha and beta cells. If correction for cell-cycle progression is warranted, our analysis of the mPancreas data suggests that the common approach of regressing out principal components of cell-cycle genes may remove additional biological variation of interest.

It is currently unknown what will happen if tricycle is applied to a dataset without cycling cells. In this case, all cells belong to the G0/G1 cloud, and we hypothesize that the G0/G1 cloud will be centered on the (0,0) origin and tricycle inferred cell-cycle position will be wrong. We expect to be able to diagnose this situation using internal controls, but we caution against using tricycle or other cell-cycle inference methods on a dataset without cycling cells.

Tricycle is a locked-down prediction procedure. There are no tuning parameters, neither explicitly set nor implicitly set through the use of cross-validation or alternatives. And in our applications, we have not aligned different samples to each other. That being said, we observe that different datasets exhibit small discrepancies. An example is that the precise location of peak expression in *Top2A* differs slightly from dataset to dataset. Possible sources of dataset-to-dataset variation include both biological and technical candidates such as technology and batch effects.

There are two specific sources of variation we want to highlight. First is the variation in nonzero expression. Specifically, we observe that 300–500 genes — out of a total of 500 genes — have non-zero expression in a given dataset, and we show that differences in these genes can cause changes in the shape of the embedding (Additional file 1: Figs. S29, S30, and S31). These differences in nonzero expression are also connected to the utility of using a larger set of genes to define the embedding space, as discussed above.

Second, the variation in the proportion of cells in G0 and G1, which is associated with the actual wall clock length of the cell cycle. Reflecting the biology of the system, this ratio also affects the placement of the origin of the projected data. However, this also potentially complicates across data set comparisons, as the only normalization currently performed in tricycle is the mean centering of each gene, which is susceptible to differences in this ratio. Lastly, we note that the peak expression location variations might be a consequence of different cell-cycle dynamics in different systems. We hope the biological implications will be examined closely in the future. Methods to expand tricycle to allow cross data comparisons are currently an active area of research.

We anticipate that the ability to model cell cycle as the continuous process that it is, will enable considerable advancements in the modeling of developmental and disease processes in which it plays a major role.

## Conclusions

We have explained why the cell cycle — in datasets where the primary source of variation is cell cycle — is visible as an ellipsoid shape in the principal components of the data. We have shown that principal component analysis of cell-cycle genes sometimes reflects other processes such as differentiation. We have proposed to use projections into a reference embedding to isolate the specific cell-cycle signal in a dataset with many sources of variation, and we have shown that this approach allows us to isolate a specific, pre-specified signal.

We have shown that tricycle is capable of inferring continuous cell-cycle position and can be applied to datasets with multiple cell types, across species and a variety of single-cell technologies from relatively deeply sequenced plate based technologies to shallower sequenced droplet-based technologies, including very sparse data. As part of applying tricycle, a user can use internal controls to assess the validity of the inferred cell-cycle positions on their own dataset. Tricycle is highly scalable and available in an open-source implementation from the Bioconductor project.

## Methods

### Using principal component analysis to recover time ordering

We will consider the following statistical model. The mean expression of each gene is modeled as

$$f_{g} (\theta) = A_{g} \cos(\theta - d_g) $$

Here, *A*_*g*_ is a gene-specific amplitude and *d*_*g*_ is a mean-specific displacement (location of the peak). In this formulation, the mean function has a single peak and is periodic. We have *G* genes and each gene has its own (but not necessarily unique) (*A*_*g*_,*d*_*g*_). We are assuming at least two peak locations not separated by *π* (i.e., two different *d*_*g*_’s).

Basic trigonometry yields the identity

$$\begin{array}{*{20}l} f_{g}(\theta) &= A_{g} \cos(\theta - d_{g}) \\ &= A_{g} \cos(d_{g}) \cos(\theta) + A_{g} \sin(d_{g}) \sin(\theta) \end{array} $$

which we can write as

$$\begin{array}{*{20}l} f_{g}(\theta) &= \left(A_{g} \cos(d_{g}) \sqrt{\pi} \right) \phi_{1}(\theta)\\ &+ \left(A_{g} \sin(d_{g}) \sqrt{\pi} \right) \phi_{2}(\theta) = a_{g}^{t} \phi(\theta) \end{array} $$

using the orthonormal functions

$$ \theta \mapsto \phi(\theta) = (\phi_{1}(\theta), \phi_{2}(\theta)) = \frac{1}{\sqrt{\pi}} (\cos(\theta), \sin(\theta)) $$

Our derivation is based on Ramsey and Silverman [40] section 8.4. This section shows that the variance-covariance operator is given by

$$v(s,\theta) = \phi^{t}(s) \left(G^{-1} \mathbf{C}^{t} \mathbf{C} \right) \phi(\theta) $$

where the inner matrix (which turns out to determine the principal components) is a 2×2 matrix equal to

$$\begin{array}{*{20}l} \frac{1}{G} \mathbf{C}^{t}\mathbf{C} &= \frac{1}{G} \sum_{g} a_{g} a_{g}^{t} = \frac{1}{G} \sum_{g} A_{g}^{2} \pi \phi(d_{g}) \phi(d_{g})^{t} \\ &= \frac{1}{G} \sum_{g} A_{g}^{2} \left(\begin{array}{cc} \cos^{2}(d_{g}) & \cos(d_{g}) \sin(d_{g}) \\ \cos(d_{g})\sin(d_{g}) & \sin^{2}(d_{g}) \end{array}\right) \end{array} $$

The principal component analysis is given by the Eigen-functions and -values of the variance-covariance operator. Such an Eigen-function and -value pair *ξ*,*ρ* takes the form

$$ \xi(\theta) = b^{t} \phi(\theta) $$

for a vector *b* which satisfies

$$ G^{-1}\mathbf{C}^{t}\mathbf{C} b = \rho b $$

i.e., *b*,*ρ* are Eigen-vectors and -values for the *G*^−1^**C**^*t*^**C** matrix. Specifically, if *q*_1_,*q*_2_,*λ*_1_,*λ*_2_ are two such Eigen-vectors- and -values, then the two first principal components are given by

$$ \theta \mapsto \xi(\theta) = (\cos(\theta), \sin(\theta))^{t} q_{i} \sqrt{G} \sqrt{\lambda_{i}} $$

### Simulations

For Fig. 1 we performed the following simulation. Fifty realization of a cosine function with a location of 0.2 and an amplitude of 0.5 as well as 50 realizations of a cosine function with a location of 1.2 and an amplitude of 1. Each function was evaluated on an equidistant grid of 1000 points and independent Gaussian noise with a standard deviation of 0.2 was added. The depictions in Fig. 1a, b were each one of the realizations of the two different cosine functions.

For Additional file 1: Figs. S1, S2 and S3, we simulated data using the negative binomial distribution, inspired by the setup in Splatter [41]. In addition to a gene-specific amplitude (*A*_*g*_) and location of the peak (*L*_*g*_), we also consider different library size (*l*), which is an approximate as we still have some cell-to-cell variance. For a cell, we let $\lambda ^{0}_{g} = A_{g} \cos (\theta - L_{g}) + c$, with *c* a constant to ensure positivity of $\lambda ^{0}_{g}$. Then the cell mean is $\lambda '_{g} = l \cdot \lambda ^{0}_{g} / \sum ^{G} \lambda ^{0}_{g}$. The trended cell mean is simulated from a Gamma distribution as *λ*_*g*_∼*Gamma*(1/*B*^2^,*λg*′*B*^2^), with *B* the biological coefficient of variation (we fix *B* as 0.1 in our simulations). Thus, the counts for gene *g* is given as *y*_*g*_∼*Pois*(*λ*_*g*_). We always simulate a 100 genes times 5000 cells count matrix, with cell timepoint *θ* uniformed distributed between 0 and 2*π*. We only vary one of *L*_*g*_,*A*_*g*_ and *l* in Additional file 1: Figs. S1, S2, and S3. Specifically, in Additional file 1: Fig. S1, we used different number of distinct peak locations across 100 genes and fixed the amplitudes (across 100 genes) as 3 and library size as 2000. In Additional file 1: Fig. S2, we used different numbers of distinct amplitudes across 100 genes and fixed the number of distinct peak locations (across 100 genes) to 100 and library size to 2000. In Additional file 1: Fig. S3, we changed the library size *l* and fixed the number of distinct peak locations (across 100 genes) as 100 and the amplitudes (across 100 genes) as 3. PCA was performed on the library size normalized and log2 transformed matrix after we got the count matrix.

### Generation of mouse primary hippocampal NPC scRNA-seq dataset

Hippocampal neural stem/progenitor cells (NPCs) were isolated by microdissection from E17 day embryos (offspring of male *Kmt2d* ^+/*βgeo*^ and female C57Bl/6J) and cultured on Matrigel as described in [24]. Cells were maintained in an undifferentiated state via supplementation with growth factors (EGF, FGF2) in Neurobasal media. In a prior publication, we have demonstrated that the *Kmt2d* ^+/*βgeo*^ cells exhibit defects in proliferation [24]. Replicate cultures from both genotypes were collected at the undifferentiated state (day 0) and then 2, 4, and 8 days after growth factor removal to induce neuronal differentiation. Cells were collected via trypsinization and pellets were washed and resuspended in Neurobasal media. scRNA-seq libraries were created with a Chromium Single-Cell 3’ library & Gel Bead Kit v3 (10x Genomics) according to the manufacturer’s protocol. Approximately 10,000 cells were targeted for each library. Only cells from day 0 are analyzed here.

### Generation of mouse E14.5 Neurosphere scRNA-seq dataset

Cortical neurospheres were generated from the dissociated telencephalon of embryonic day 14.5 (E14.5) wild-type embryos. Embryos were harvested and the dorsal telencephalon was dissected away and collected in 1X HBSS at RT temperature. The dorsal telencephalon was gently triturated using p1000 pipette tips and the resultant cell suspension was spun at 500G for 5min and the media was aspirated off. The cell pellets were resuspended in complete neurosphere media 7ml (CNM) and plated in ultra-low adherence T25 flasks. CNM is made by combining 480ml DMEM-F12 with glutamine, 1.45g of glucose, 1X N2 supplement, 1X B27 supplement without retinoic acid, 1x penicillium/streptomycin, and 10ng/ml of both epidermal growth factor (EGF) and basic fibroblast growth factor (bFGF). The cell pellets were cultured for 3–5 days, or until spheroids have formed. The neurospheres were then collected and spun at 100G for 5min and the supernatant was removed. Neurospheres were resuspeneded in 5ml TrypLE and incubated for a maximum of 5min at 37 ^∘^*C* with gentle trituration every 1.5min with a p1000 until the neurospheres are mostly a single-cell suspension. The cells were spun down at 500 G for 5 min and the supernatant was removed. The cells were resuspended in 15 ml of CNM and gently passed through a 40-uM filter to remove large cell clumps. The resultant cell suspension was then plated in T75 flasks for another 2–5 days or until spheres began to have dark centers. This process was repeated two more times before cells were collected for 10X Genomics single-cell library prep. Before single-cell library preparation, the neurospheres were dissociated as described above and passed through a 40-uM filter to ensure a single-cell suspension. Approx. 7000 cells were selected from each sample for input to the scRNA-seq library prep. scRNA-seq libraries were created using the Chromium Single-Cell 3’ library & Gel Bead Kit v3 (10x Genomics) according to manufacturer protocol.

### Reference genome and mapping index building

For mouse, GRCm38 reference genome fasta file and primary gene annotation GTF file (v25) were downloaded from GENCODE (https://www.gencodegenes.org). Similarly, GRCh38 reference genome fasta file and primary gene annotation GFT file(v35) were downloaded for human. We built a reference index for use by alevin as described in [42] using R package eisaR(v1.2.0), which we use to quantify both spliced and unspliced counts of annotated genes.

### scRNA-seq preprocessing

#### Mouse Neurosphere (mNeurosphere) dataset

fastqs files were used to quantify both spliced and unspliced counts by Alevin (Salmon v1.3.0) with default settings as described in [42]. Abundances matrices were read in by R package tximeta (v1.8.1). The spliced counts were treated as the expression counts. We removed cells with less than 200 expressed genes, and cells flagged as outliers (deviating more than triple median absolute deviations(MAD) from the median of log2(TotalUMIs), log2(number of expressed genes), percentage of mitochondrial gene counts, or log10(doublet scores)). The doublet scores were computed using doubletCells function in R package scran (v1.18.1). All mitochondrial genes and any genes which were expressed in less than 20 cells were further excluded from all subsequent analyses. Expression abundances were then library size normalized and log2 transformed by function normalizeCounts in R package scuttle (v1.0.2). The biological samples were integrated by Seurat (v3.2.2). We then run PCA on the top 2000 highly variable genes of the integrated log2(expression) using the runPCA function with default parameters, followed by running the runUMAP function on the resulting top 30 principal components with default parameters. Note that we did not restrict genes to cell-cycle genes in this step, as we would like to see the overall variation of the data. Cell types were inferred by SingleR package v1.4.0 using built-in MouseRNAseqData dataset as the reference.

#### Mouse primary hippocampal NPC (mHippNPC) dataset

All preprocessing are the same as for the mouse Neurosphere (mNeurosphere) dataset.

#### Mouse developing pancreas (mPancreas) dataset

We obtained the spliced and unspliced count matrices of the Mouse developing pancreas dataset from the python package scvelo (v0.2.1). The spliced counts were treated as the expression counts. We removed cells with less than 200 expressed genes, and any cells flagged as outliers (deviating more than triple median absolute deviations (MAD) from the median of log2(TotalUMIs), log2(number of expressed genes), percentage of mitochondrial gene counts, or log10(doublet scores)). Here, the doublet scores were computed using doubletCells function in R package scran (v1.18.1). All mitochondrial genes and any genes which were expressed in less than 20 cells were further excluded from all subsequent analyses. Expression abundances were then library size normalized and log2 transformed by function normalizeCounts in R package scuttle (v1.0.2). We run PCA on the top 500 highly variable genes using the runPCA function with default parameters, followed by running the runUMAP function on the resulting top 30 principal components. When running the UMAP, we set *min_dist* to 0.5 instead of default value 0.01 to replicate the UMAP figure shown in [43] with other parameters default. Of note, the single-cell libraries of the data was generated using 10x Genomics’ Chromium v2 system.

#### Mouse Hematopoietic Stem Cell (mHSC) dataset

We downloaded processed log2 transform TPM matrix directly from GEO under accession number GSE59114 [33]. We only used the cells from C57BL/6 strain, of which contains more cells, as the number of overlapped genes between xlsx file of C57BL/6 strain and DBA/2 strain is too small. Because the data was already processed and filtered, we did not perform any other processing. Unlike the abovementioned dataset, the SMARTer protocol was applied during library preparation.

#### Mouse Retina (mRetina) dataset

This dataset is available at https://github.com/gofflab/developing_mouse_retina_scRNASeq[34]. We removed cells flagged as outliers (deviating more than triple median absolute deviations (MAD) from the median of log2(TotalUMIs), log2(number of expressed genes), percentage of mitochondrial gene counts, or log10(doublet scores)). As the total UMIs depend on cell type, we filtered the cells by blocking for each cell type. The doublet scores were computed using doubletCells function in R package scran (v1.18.1). All mitochondrial genes and any genes which were expressed in less than 20 cells were further excluded from all subsequent analyses. Expression abundances were then library size normalized and *log*_2_ transformed by function normalizeCounts. We used the cell type annotations as the *new_CellType* column in the provided phenotype file. The single-cell libraries of the data was generated using 10x Genomics’ Chromium v2 system.

#### HeLa cell lines datasets

The spliced and unspliced count matrices of HeLa Set 1 (HeLa1) and HeLa Set 2 (HeLa2) were downloaded from GEO website with accession number GSE142277 and GSE142356 [15]. Both datasets were generated by the same lab under the same protocol, while the sequencing depth of Set 2 is only about half that of Set 1. For each dataset, we only used the genes existing in both spliced and unspliced count matrices. The spliced counts were treated as the expression counts. We removed cells with less than 200 expressed genes, and cells flagged as outliers (deviating more than triple median absolute deviations (MAD) from the median of log2(TotalUMIs), log2(number of expressed genes), percentage of mitochondrial gene counts, or log10(doublet scores)). All mitochondrial genes and any genes which were expressed in less than 20 cells were further excluded from all subsequent analyses. Expression abundances were then library size normalized and log2 transformed by the function normalizeCounts. The single-cell libraries of the data were generated using Drop-seq system.

#### Mouse embryonic stem cell (mESC) dataset

The processed count matrix was downloaded from ArrayExpress website under accession number E-MTAB-2805 (https://www.ebi.ac.uk/arrayexpress/experiments/E-MTAB-2805/) [9]. We only retained 279 cells with *log*_2_(counts) greater than 15. The count matrix was library size normalized across cells and *log*_2_ transformed by function normalizeCounts. The RNA-seq data was generated using Fluidigm C1 system in this dataset.

#### Human embroyonic stem cells (hESC) dataset

The processed count matrix was downloaded from GEO under accession number GSE64016 [10]. We only retained FACS sorted cells. The count matrix were library size normalized across cells and log2 transformed by function normalizeCounts. The RNA-seq data was generated using Fluidigm C1 system in this dataset.

#### Human U-2 OS cells (hU2OS) dataset

The TPM matrix was downloaded from GEO under accession number GSE146773 [20]. We only retained FACS sorted cells with log2(counts) greater than the 3 times MAD range. Genes which were expressed in less than 20 cell were removed. The left TPM matrix were library size normalized across cells and log2 transformed by function normalizeCounts. The RNA-seq data was generated using SMART-seq2 chemistry in this dataset. We got the FUCCI coordinates and FUCCI pseudotime directly from the authors of [20] (version 1.2).

#### Human induced pluripotent stem cells (hiPSCs) dataset

The processed FUCCI intensity and RNA-seq data was downloaded from https://github.com/jdblischak/fucci-seq/blob/master/data/eset-final.rds?raw=true. The preprocessing was described in [14]. The count matrix were library size normalized across cells and log2 transformed by function normalizeCounts. The RNA-seq data was generated using Fluidigm C1 system in this dataset.

#### Fetal tissue dataset

We got the loom file containing gene counts of all tissue from GEO under accession number GSE156793 [35]. We then processed and analyzed each tissue separately. For each tissue type, cells of which log2(TotalUMIs) is lower than median−3×MAD, and genes expressed in less than 20 cells were excluded from further analyses. The count matrix was library size normalized across cells and log2 transformed by function normalizeCounts. All 4 tissues profiled using single-cell and 9 tissues profiled using single-nuclei were generated on sci-RNA-seq3 system.

### Five-stage cell-cycle assignments

The 5-stage (G1S, S, G2, G2M, and MG1) cell-cycle assignments were adapted from [15] with some modifications. Briefly, the assignments use the high expression genes list for each stage, curated by [7]. Let *k* represent one of the 5 stages, and $l_{k} = \left \{g_{k}^{1}, g_{k}^{2}, \cdots, g_{k}^{p_{k}}\right \}$ represent the gene list with *p*_*k*_ genes. For each stage *k*, we could calculate the mean expression across genes in the gene list *l*_*k*_ for the *j*th cell as $m_{j,k}= \frac {1}{p_{k}} \Sigma _{g_{k}^{i} \in l_{k}}E_{g_{k}^{i}, j}$ with $E_{g_{k}^{i}, j}$ as the log2 transformed expression value of gene $g_{k}^{i}$ and cell *j*. Then we assess how well a gene in a gene list correlates to the mean expression level of that gene list as $c_{g_{k}^{i}} = \text {cor}\left (E_{g_{k}^{i}}, m_{k} \right)$. For each stage, the gene list is pruned to genes with $c_{g_{k}^{i}} > 0.2$. (For the fetal tissues dataset, we used $c_{g_{k}^{i}} > 0.15$ since the extremely shallowly sequenced data shows less co-expression patterns and the threshold 0.2 could leave us with no genes.) We label this pruned new gene list as $L_{k} = \left \{g_{k}^{1}, g_{k}^{2}, \cdots, g_{k}^{q_{k}}\right \}$ with *q*_*k*_ the number of genes. The stage assignment score for cell *j* and stage *k* is given as

$$A_{k,j} = \frac{1}{q_{k}} \Sigma_{g_{k}^{i} \in L_{k}}E_{g_{k}^{i}, j} $$

The 5-by-n matrix **A**, of which the number of columns equals to the number of cells, follows *z*-score transformations w.r.t. first rows and then columns, resulting the 5-by-n matrix $\mathbf {\widetilde {A}} = (\widetilde {A}_{k,j})$. For each cell, we compute the preliminary stage assignment as $s_{j} = \underset {k}{\text {arg\,max}}\{ \widetilde {A}_{k,j}\}$.

As in the [15], we also apply two filtering steps. The first filtering, which is unchanged from the original method, is as follows. We require $s_{\tilde {j}}$, the stage with the second largest assignment score, to be the neighboring stage to *s*_*j*_. This requirement corresponds to the 5 stages being continuously cyclic processes.

As for the second filtering step, the original method discards all cells with the second largest assignment score $\widetilde {A}_{s_{\tilde {j}},j} > 0.75$. We found the threshold of 0.75 to some extent not applicable, as in some datasets it leads to losing 90% of cells. Therefore, we use a more adaptive threshold by requiring $\widetilde {A}_{s_{j},j} - \widetilde {A}_{s_{\tilde {j}},j} > 0.3$.

If the cell passes two filtering steps, it will be assigned to a stage *s*_*j*_. Otherwise, it would be assigned as *NA* w.r.t. 5 stages of the cell cycle. To mitigate the batch effect on the 5 stage assignments, the assigning procedures are done for each sample/batch separately within each dataset, as recommended in Revelio package [15].

### PCA of GO cell-cycle genes

For each dataset, we subsetted the preprocessed log2 transformed expression matrix to genes in the GO term cell cycle (GO:0007049). If there are clear batches defined in the dataset, such as sample or batch, we use Seurat3 to remove batch effect. In the case of using Seurat3, we used a library size normalized count matrix as input instead of log2 transformed values. The integration anchors were searched in the space of the top 30 PCs. The output integrated matrix is a log2 transformed matrix of the top 500 most variable genes. We then performed principal component analysis on the gene-wise mean centered expression matrix. In the case of no batch exiting, we also restricting to the top 500 variable genes among GO cell-cycle genes.

### Projection of new data to cell-cycle embedding and calculation of cell-cycle position *θ*

The projection using pre-learned weights matrix during PCA of GO cell-cycle genes is straight forward, given by

$$\mathbf{P} = \Tilde{\mathbf{E}} ^ t \cdot \mathbf{R} $$

where **R** represents the o-by-2 reference matrix (*o*≤500), contains the weights of top 2 PCs learned from PCA of GO cell-cycle genes; **E** ~ is a o-by-n matrix, subsetted from **E** (the log2 transformed expression matrix) with genes in the weights matrix and row-means centered. The resulting n-by-2 **P** is the cell-cycle embedding projected by the reference. The calculation of the cell-cycle position *θ* is given by

$$\theta = \arctan\left(\frac{P_{2}}{P_{1}}\right) $$

where *P*_*i*_ is the *i*th column of matrix **P**. When mapping the genes between weights matrix and the data that we want to project, the Ensemble ID is given higher priority than the gene symbol for mouse. For across species projection, we only consider the homologous genes of the same gene symbols.

### Periodic loess

As *θ* is a circular variable bound between 0 to 2*π*, fitting a traditional loess model *y*∼*θ*, with *y* as any response variable, such as the gene expression of gene, or log2(TotalUMIs), has problems around the boundaries 0 and 2*π*. Hence, we concatenate triple *y* and triple *θ* with one period shift to form [*y*,*y*,*y*] and [*θ*−2*π*,*θ*,*θ*+2*π*], on which the loess line is fitted. We then only use the fitted value $\hat {y}$ when *θ* is between 0 and 2*π* for visualization purpose.

The calculation of the coefficient of determination *R*^2^ of the fitted loess model is given by

$$R^{2} = 1 - \frac{{SS}_{\text{res}}}{{SS}_{\text{total}}} $$

Here ${SS}_{\text {res}} = \sum _{i}^{n} (y_{i} - \hat {y_{i}})^{2}$ and ${SS}_{\text {total}} = \sum _{i}^{n} (y_{i} - \bar {y})^{2}$. Note that instead of using all three copies of data points, we restrict the calculation of *SS*_res_ and *SS*_total_ on the original data points (the middle copy). The residuals are not the same for the three copies, especially at the beginning and end of [−2*π*,2*π*].

### The circular correlation coefficient *ρ*

We use the circular correlation coefficient *ρ* defined by [44] to evaluate concordance between two polar vectors *θ*_1_ and *θ*_2_. It is defined as follows

$$\rho = \frac{\Sigma [\sin(\theta_{1} - \mu_1) \cdot \sin(\theta_{2} - \mu_2)]}{\sqrt{\Sigma [\sin^{2}(\theta_{1} - \mu_1)] \cdot \Sigma [\sin^{2}(\theta_{2} - \mu_2)]}} $$

*μ*_1_ and *μ*_2_ represent the mean of *θ*_1_ and *θ*_2_ respectively, and are estimated by maximum likelihood estimation under von Mises distribution assumption.

### Running other methods

For other cell-cycle inference methods, we use all default parameters and its built-in reference (if needed) in the following packages: cyclone in scran (v1.18.5), CellCycleScoring in Seurat (v4.0.0.9015), Revelio (v0.1.0), peco (v1.1.21), and reCAT (v1.1.0).

### Silhouette index on angular separation distance of tricycle cell-cycle position *θ*

For cyclone and Seurat, we could use Silhouette index to describe consistency between discretized cell-cycle stage and tricycle cell-cycle position *θ*. We use angular separation distance metric to quantify the distance between cell *i* and cell *j* as

$$d(i, j) = 1 - \cos(\theta_{i} - \theta_j) $$

For a cell $\phantom {\dot {i}\!}i \in S_{k^{(i)}} \ni k^{(i)} \in \{G1, S, G2M \}$. The mean distance between cell *i* and all other cells assigned to the same stage

$$a(i) = \frac{1}{|S_{k^{(i)}}| - 1} \sum_{j \in S_{k^{(i)}}, i \not = j} d(i, j) $$

with $\phantom {\dot {i}\!}|S_{k^{(i)}}|$ the cardinality of $\phantom {\dot {i}\!}S_{k^{(i)}}$. Specially, *a*(*i*)=0 if $\phantom {\dot {i}\!}|S_{k^{(i)}}| = 1$.The mean distance from cell *i* to all cells assigned to other stage *k*^′^ such that *k*^′^≠*k*^(*i*)^∧*k*^′^∈{*G*1,*S*,*G*2*M*} is

$$b(i) = \min_{k' \not = k^{(i)}} \frac{1}{|S_{k'}|} \sum_{j \in S_{k'}} d(i, j) $$

The Silhouette index for cell *i* is given as

$$s(i) = \left\{\begin{array}{ll} \frac{b(i) - a(i)}{\max\{ a(i), b(i)\}} & \text{if}\ |S_{k^{(i)}}| > 1 \\ 0 & \text{if}\ |S_{k^{(i)}}| = 1 \end{array}\right. $$

For any cell *i*, the Silhouette index *s*(*i*) is bound between −1 and 1 (−1≤*s*(*i*)≤1). An *s*(*i*) close to 1 means the cell is consistently assigned to its neighbors w.r.t. its cell-cycle position *θ*_*i*_. An *s*(*i*) close to −1 means the cell is closer to the other stage. An *s*(*i*) equals to 0 means the cell is on the border of two stages. The mean Silhouette index on all cells measures how tight the stage assignments are. In this context, this value must be interpreted carefully as it is different from traditional clustering which might put hard boundaries and gaps between clusters. As the cell-cycle process is continuous in nature, there must be cells assigned on the boundaries and ambiguous to either stage, and no gap should appear between stages. Thus, the mean silhouette index greater than 0 might be appropriate to conclude the agreement between tricycle cell-cycle position *θ* and discretized cell cycle stages.

## Supplementary Information

**Additional file 1** Supplementary Materials. Supplementary methods about comparison with existing cell cycle tools and supplementary figures (Fig. S1-S36).

**Additional file 2** Review history.

## Data Availability

The mouse E14.5 Neurosphere data is available under GEO accession number GSE171636 [45]. The mouse primary hippocampal NPC data is available under GEO accession number GSE190514 [46]. Other public datasets used from GEO or the corresponding webpage: mouse developing pancreas (mPancreas) dataset (https://scvelo.readthedocs.io/scvelo.datasets.pancreas/#scvelo.datasets.pancreas[32, 43]), mouse hematopoietic stem cell (mHSC) dataset (GSE59114 [33]), mouse retina (mRetina) dataset (https://github.com/gofflab/developing_mouse_retina_scRNASeq [34]), Hela cell lines datasets (GSE142277 and GSE14235 [15]), mouse embryonic stem cell (mESC) dataset (https://www.ebi.ac.uk/arrayexpress/experiments/E-MTAB-2805/ [9]), human embroyonic stem cells (hESC) dataset (GSE64016 [10]), human U-2 OS cells (hU2OS) dataset (GSE146773 [20]), human induced pluripotent stem cells (hiPSCs) dataset (https://github.com/jdblischak/fucci-seq/blob/master/data/eset-final.rds?raw=true [14]), and fetal tissue dataset (GSE156793 [35]). All code to analyze the data and generate figures is available at https://github.com/hansenlab/tricycle_paper_figs[47]. The processed data is available at 10.5281/zenodo.5519841 [48]. The tricycle method is implemented in the R package tricycle containing the mNeurosphere reference, which is available on https://github.com/hansenlab/tricycle [49] and on Bioconductor under the GPL-3 license (https://www.bioconductor.org/packages/tricycle [50]).
