# Supplementary material for: Universal prediction of cell-cycle position using transfer learning
Source: Genome Biol. 2022 Jan 31;23:41. doi: 10.1186/s13059-021-02581-y (PMC8802487; doi:10.1186/s13059-021-02581-y)
Supplement: Supplementary file 1 — Additional file 1 Supplementary Materials. Supplementary methods about comparison with existing cell cycle tools and supplementary figures (Fig. S1-S36). [file 13059_2021_2581_MOESM1_ESM.pdf]

SUPPLEMENTARY MATERIALS

Universal prediction of cell cycle position using transfer learning

Shijie C. Zheng, Genevieve Stein-O'Brien, Jonathan J. Augustin, Jared Slosberg, Giovanni A. Carosso, Briana Winer, Gloria Shin, Hans T. Bjornsson, Loyal A. Goff*, and Kasper D. Hansen*

* Correspondence to loyalgoff@jhmi.edu (LAG), khansen@jhsph.edu (KDH)

Contents

- Supplementary Methods.
- Supplementary Figures: Fig. S1-S36.

SUPPLEMENTARY METHODS

Comparison with existing cell cycle tools

Oscope

Oscope poses significant challenges when run on shallow data (10X, sci-RNA-seq3, or DropSeq), since the method requires quantification of a high number of genes in every cell. For this reason, we do not evaluate Oscope.

peco

Peco supplies 2 models: one trained on 101 genes and one trained on 5 genes. We used the 101 gene model to be robust to some genes not being measurable in all datasets. We applied peco to all dataset described in Table 1, except mRetina and human fetal tissues. For human fetal tissues, we only use a subset of 2000 random cells selected from human fetal intestine data (termed "hfIntestineSub").

We assess the expression dynamics of 4 genes highlighted in Hsiao et al. (2020): *CDK1*, *TOP2A*, *UBE2C* and *H4C3* (Fig. S16); not all datasets have these genes measured in which case they are absent from the figure. To systematically compare tricycle and peco we use the R^2 associated with two different cell cycle positions. This is a comparison between R^2 for the same data, but using the same periodic loess approach with two different position variables. For these genes, across all dataset, tricycle cell cycle position has a higher R^2 than peco cell cycle position (Fig. S16). Generally, information-rich Fluidigm C1 data does better with peco compared to information-poor 10X, Drop-Seq.

Revelio

Revelio is designed to search for an ellipsoid pattern amongst (rotated) principal components, by finding the directions having the strongest association with 5 discrete cell cycle stages. The output of Revelio is therefore supposed to be an ellipsoid. Revelio by itself does not quantify cell cycle position, although it seems natural to do so by the angle. When we use Revelio, we do indeed observe an ellipsoid in 4 datasets (Fig. S17a, b, f, g, i and j), but it clearly fails in 3 datasets: mPancreas dataset, mRetina dataset, and mHSC dataset (Fig. S17c, d, and e). These 3 datasets all have substantial variation which is not associated with cell cycle, such as cell types and differentiation, which we believe explains the non-ellipsoidal embedding. For example, in the mPancreas data, some of the differentiation effect is perfectly confounded with cell cycle as the terminally differentiated cells stop cycling. It is not clear that simply rotating the principal components will help us find a better cell cycle exclusive dimension. Additionally, Revelio removes any cell which does not have a prediction using the Schwabe stage predictor; in the mRetina dataset only 30k out of more than 90k cells are retained.

reCAT

reCAT starts with a principal component analysis of the cell cycle genes, and infers an ordering by solving a traveling salesman problem on this representation. This produces an ordering, but this ordering is hard to interpret because it is not directly linked to cell cycle stage. To address this, the authors provide two different stage predictors. Because the method requires the solution of a traveling salesman problem, it scales poorly. Due to these issues, we only ran reCAT on data with less than 5000 cells. The orderings inferred by reCAT are largely consistent with our cell cycle position θ using mNeurosphere reference for all datasets except the most shallow sequenced hfIntestineSub data (Fig. S18 last sub-panel in each panel). And the expression dynamics of Top2A on the time series also confirms the appropriate ordering of cells (Fig. S18 the third subpanel in each panel). However, the two-stage predictors given by reCAT yield different predictions on stages. For example, for the mPancreas dataset (Fig. S18a), the majority of cells are at S stage based on Bayes scores but are at G1 stage based on mean scores. Note that the reCAT

function requires the user to feed an approximate cutoff position to assign a cell cycle stage based on Bayes scores. However, in all the datasets, we are unable to assign cutoff position to let each stage have its own highest scores interval. Without a useful stage assignment, the ability to make use of the cell orders is substantially restricted as the percentage of each stage is different across dataset.

Cyclone

We observe a general agreement between the 3 stage predictions of cyclone and tricycle cell cycle position, as the cyclone stages cluster together (Fig. S19). We note that cyclone assigns very few cells to the S stage. We believe this is caused by the assignment strategy (cells are assigned to S stage if both G1 and G2M scores are below 0.5). To expand on this comparison, we computed silhouette index with a distance defined by the tricycle cell cycle position (Methods). For cyclone, the under-representation of S stage drags down the silhouette index for both G1 and S stages, as cells at S stages are usually mixed with G1 cells, making the mean distance to all cells at G1 stage and to all cells at S stage not that differentiable. We note that cyclone works best on the last two FACS datasets, with one of them (mESC) being the training dataset for cyclone gene list.

Seurat

We observe good agreement between the 3 stage predictions of Seurat and tricycle cell cycle position, better than cyclone (Fig. S20). Compared to cyclone, we have a much higher silhouette index for Seurat; the highest observed mean is 0.74 for the mHSC dataset, which confirms the highly visual agreement between Seurat assignments and tricycle. The main disadvantage of Seurat is the inherent limitation of a 3 stage prediction.

SchwabeCC

The SchwabeCC method assigns cells to 5 different stages. Because of the higher resolution, it is the main predictor we use in our work. By default, the Schwabe method as reported in Schwabe et al. (2020) produces a substantial amount of missing labels, and we have therefore modified the method to address this (Methods); we used this modified Schwabe predictor unless specified otherwise (named as SchwabeCC).

Broadly, the SchwabeCC predictor agrees with tricycle, with one specific type of disagreement. These inconsistencies are examined in Fig. S21. Some cells with a tricycle cell cycle position of $0/2\pi$ (G0/G1) are assigned to other stages by SchwabeCC (Fig. S21 second sub-panel of each row). It is well appreciated that there are many more genes specifically expressed at S, G2 or M stage as compared to G0/G1 stage (Dolatabadi et al., 2017). For each dataset, we plot out the percentage of nonexpressed genes over all projection genes in the first sub-panels, which show that the dynamics of percentages are captured by cell cycle position θ using mNeurosphere reference. We plot the percentage of non-expressed genes conditioned on stage and whether tricycle cell cycle position is around $0/2\pi$ (Fig. S21 third sub-panel of each row), which confirms that for each stage there exist two distinct groups. This is reinforced by the different expression patterns of *Top2A* and *Smc4* between flagged cells and non-flagged cells in the last two sub-panels. Thus, we conclude the cells around $0/2\pi$ are likely to be wrongly assigned to other stages, probably due to low information content.

To assess whether these inconsistencies are caused by our modification of Schwabe, we repeat the comparison using the original Schwabe assignments and arrive at the same conclusion (Fig. S22). This assessment highlights the large number of missing labels from the original Schwabe predictor, for example only 30k out of 90k cells in the mRetina dataset are labelled.

SUPPLEMENTARY FIGURES

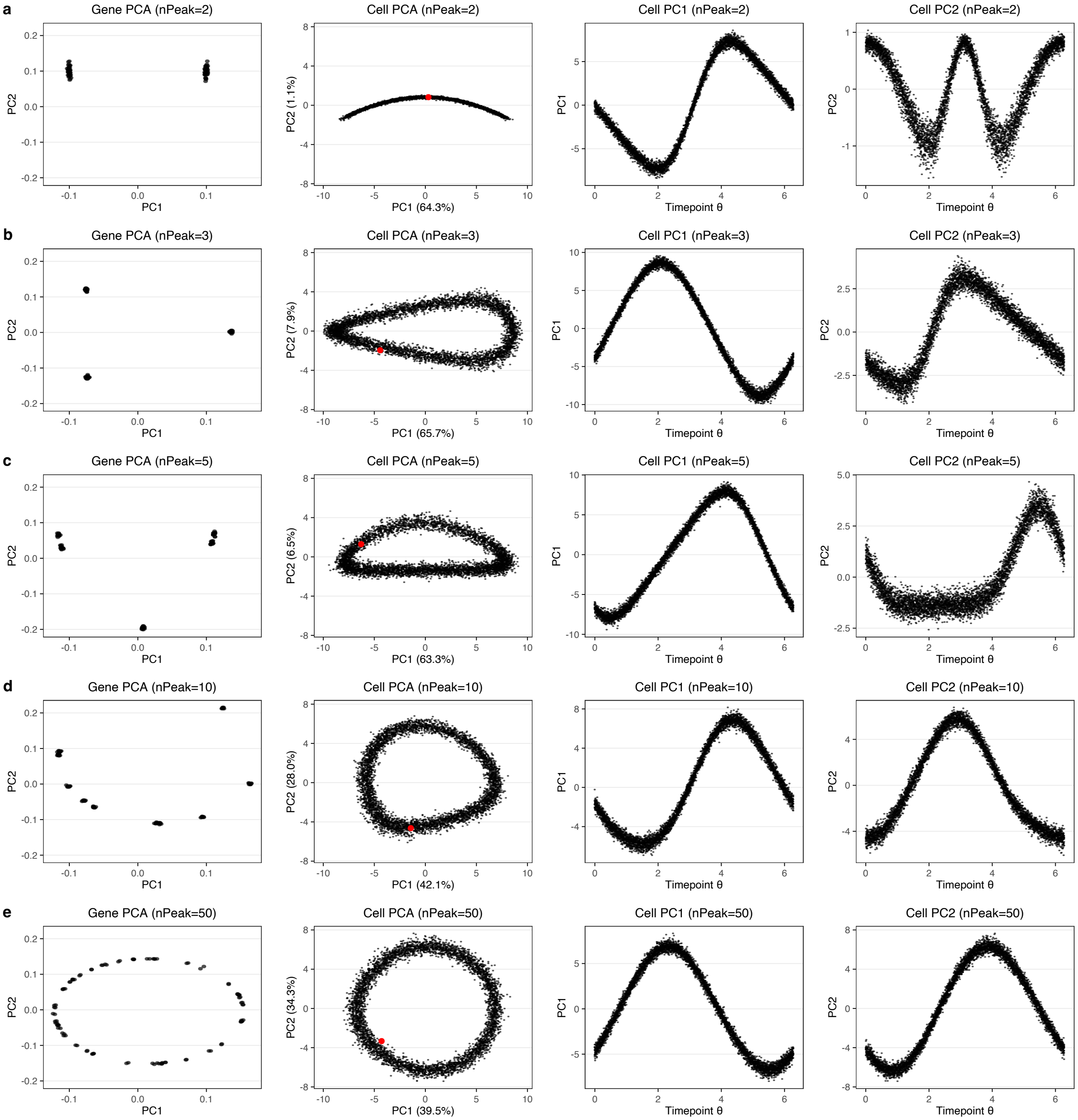

Fig. S1. Simulations using negative binomial distribution with different number of distinct peak locations. We used different number of distinct peak locations across 100 genes, and fixed the amplitudes (across 100 genes) as 3 and library size as 2000. The number of distinct peak locations across 100 genes is **(a)** 2, **(b)** 3, **(c)** 5, **(d)** 10, and **(e)** 50. As long as we have more than 2 distinct peak locations, we get an ellipsoid.

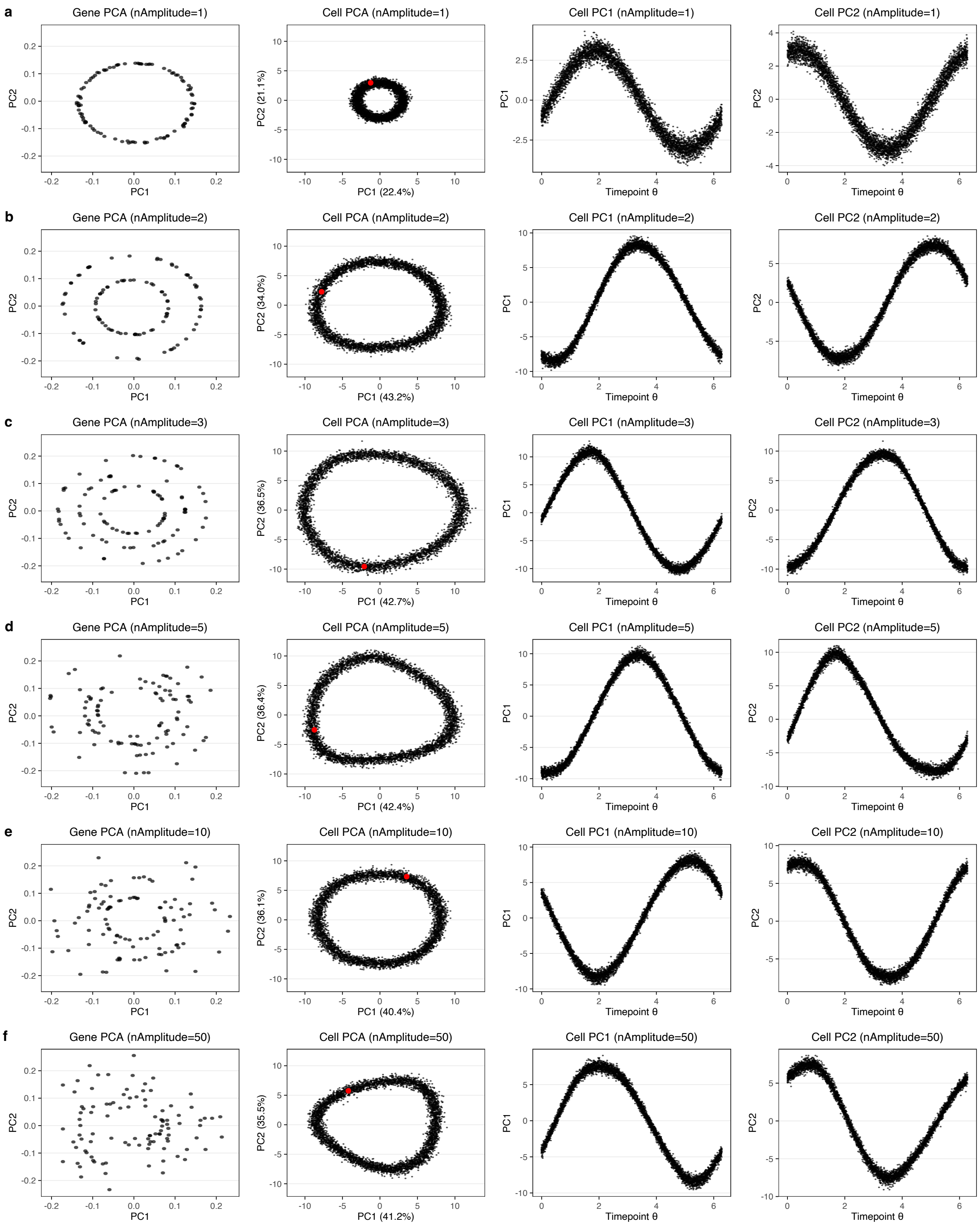

Fig. S2. Simulations using negative binomial distribution with different number of distinct amplitudes. We used different numbers of distinct amplitudes across 100 genes, and fixed the number of distinct peak locations (across 100 genes) as 100 and library size as 2000. The number of distinct amplitudes across 100 genes is **(a)** 1, **(b)** 2, **(c)** 3, **(d)** 5, **(e)** 10, and **(f)** 50. No matter what the number of distinct amplitude(s) is, we always get an ellipsoid.

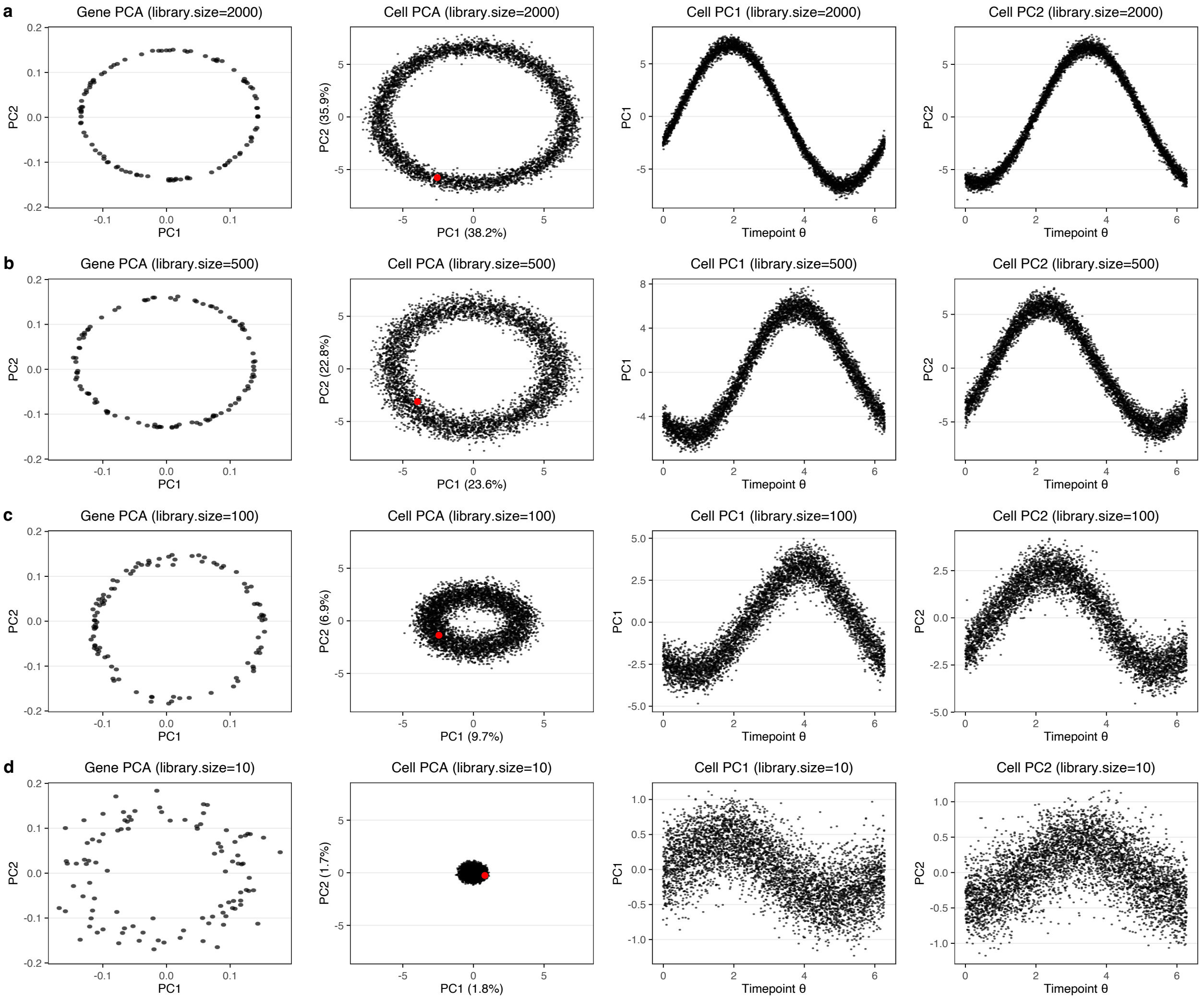

Fig. S3. Simulations using negative binomial distribution with different library size. We changed the library size l, and fixed the number of distinct peak locations (across 100 genes) as 100 and the amplitudes (across 100 genes) as 3. The library size is **(a)** 2000, **(b)** 500, **(c)** 100, and **(d)** 10. The range of x-axis and y-axis of the first two sub-panels are fixed across (a)-(d). With library size decreasing, the ellipsoid shrinks to the $(0, 0)$. However, the orders of cell can still be recovered.

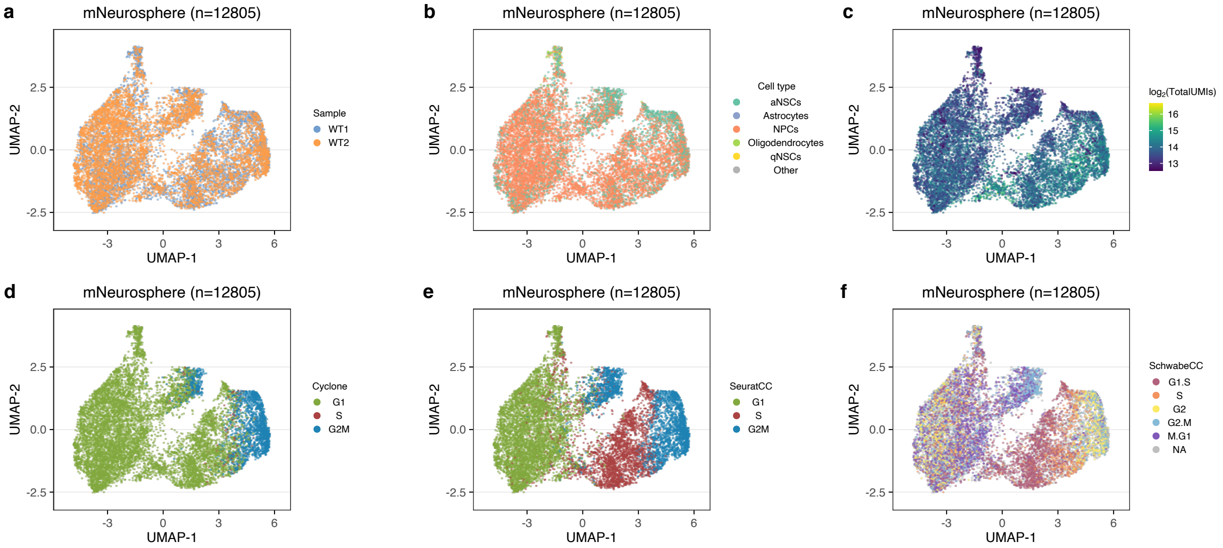

Fig. S4. UMAPs of the mouse cortical Neurosphere dataset. Scatter plots show the UMAPs of Seurat3 mergerd Neurosphere data colored by **(a)** sample, **(b)** cell type inferred by SingleR, **(c)** $\log_2$(TotalUMIs), **(d)** inferred cell cycle stage by cyclone, **(e)** inferred cell cycle stage by Seurat, **(f)** inferred cell cycle stage by the SchwabeCC method (Schwabe et al., 2020) (See Methods). The UMAP coordinates were computed using the PCA on top 2000 highly variable genes after integreation by Seurat3.

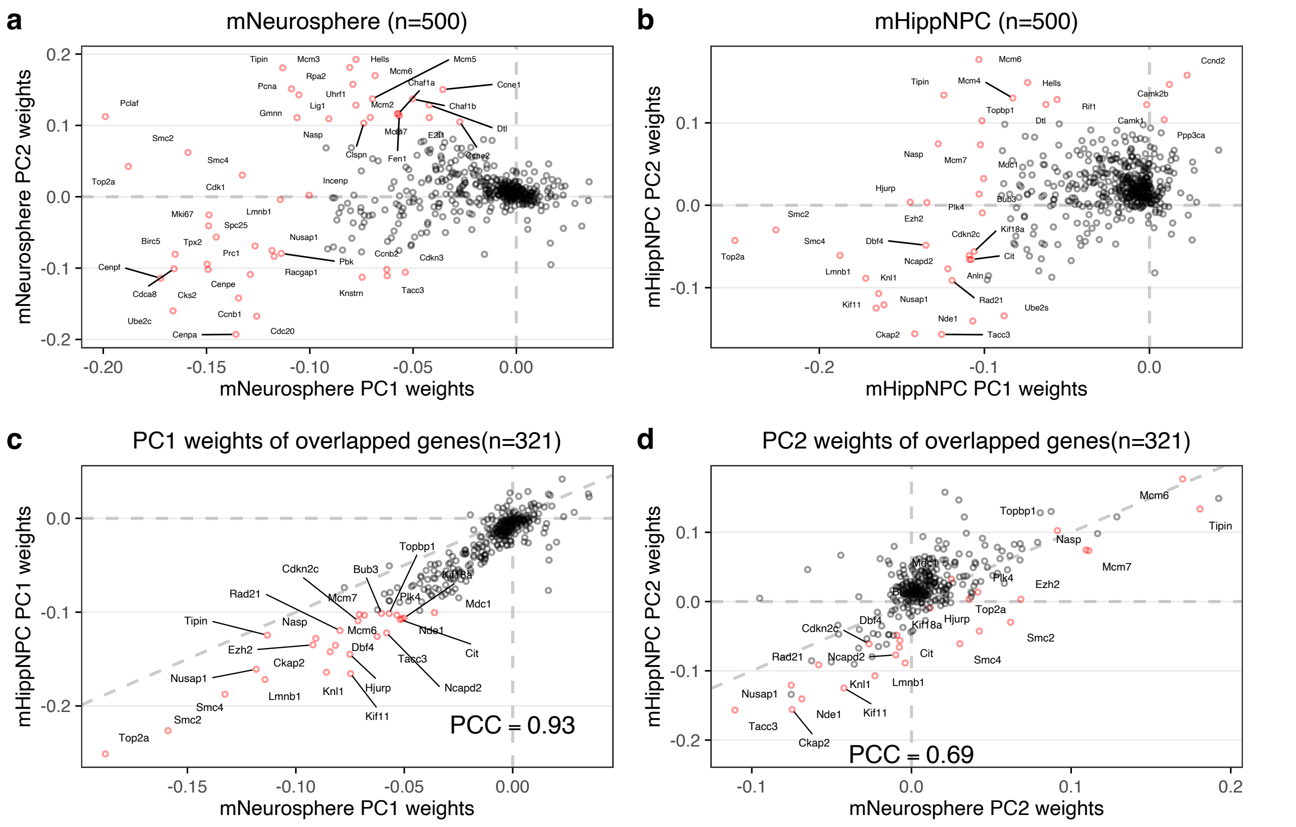

Fig. S5. Weights of PCA on GO cell cycle genes. (a) The weights of top 2 PCs learned from doing PCA on GO cell cycle genes of cortical Neurosphere data. **(b)** The weights of top 2 PCs learned from doing PCA on GO cell cycle genes of mouse primary hippocampal NPC data. **(c)** A comparison of the weights on principal component 1 between the cortical neurosphere and hippocampal progenitor datasets. **(d)** As (c), but for PC2. Genes with high weights ($|\text{score}| > 0.1$ for either vector) are highlighted in red. PCC: Pearson's Correlation Coefficient.

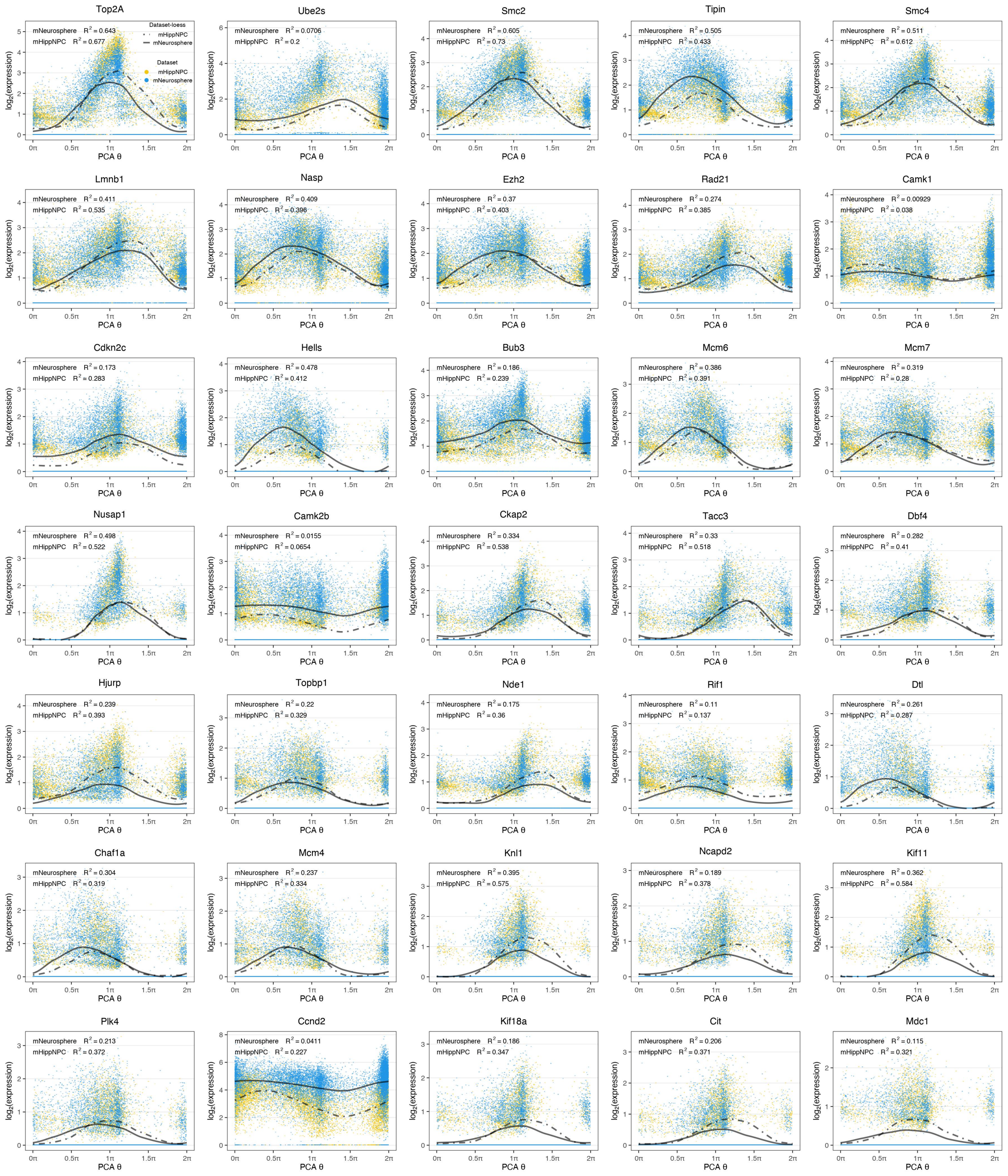

Fig. S6. Expression dynamics of top ranked genes. Similar to Figure 2d and e, but now showing all overlapped projection genes with absolute weights greater than 0.1 in either PC1 or PC2 of either dataset. Yellow points are cells of mHippNPC data, while blue points are cells of mNeurosphere data. Two loess lines were fitted for two dataset respectively. There is high agreement of the dynamics between datasets.

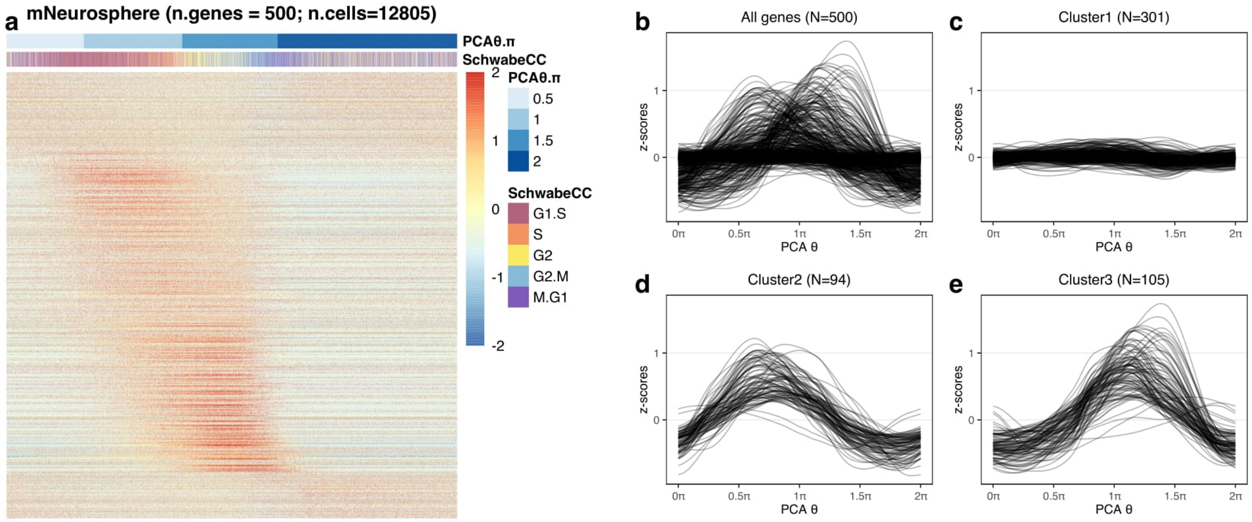

Fig. S7. Characteristics of expression patterns of the mNeurosphere reference. (a) Heatmap shows the z-scores of 500 projections genes in the mNeurosphere data. Each row represents a gene and each column represents a cell, ordered by the cell cycle position θ from PCA. We also annotate the position of half π as the cells are not uniformed distributed along 0 to 2π. **(b)** The fitted loess line of z-scores over cell cycle position θ for all 500 projection genes. **(c-e)** The three different clusters in (b). **(c)** The cluster of genes with highest z-scores less than 0.5. **(d)** The cluster of genes with highest z-scores greater than 0.5 and peak position before π This cluster corresponds to high expression genes at G1/S stage. **(e)** The cluster of genes with highest z-scores greater than 0.5 and peak position after π. This cluster corresponds to high expression genes at G2/M stage.

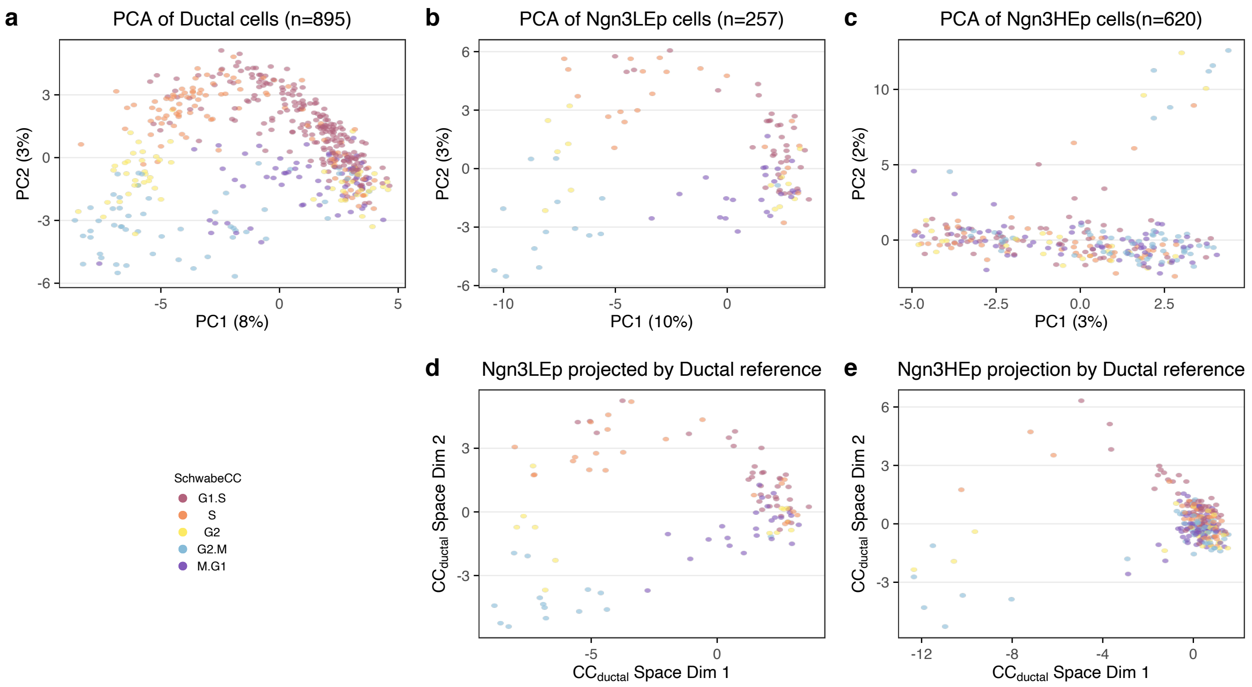

Fig. S8. PCA and projections of the mouse developing pancreas data. (a-c) The top 2 PCs of GO cell cycle genes of the the three most multipotent cell types in the mouse developing pancreas data. PCA was performed independently for each cell type. Note that panel (a) reproduces Figure 3c. **(d)** Projection of allNgn3LEP cells of mPancreas data using the learned top 2 PCs weights on GO cell cycle genes of Ductal cells. **(e)** Projection of allNgn3HEP cells of mPancreas data using the learned top 2 PCs weights on GO cell cycle genes of Ductal cells.

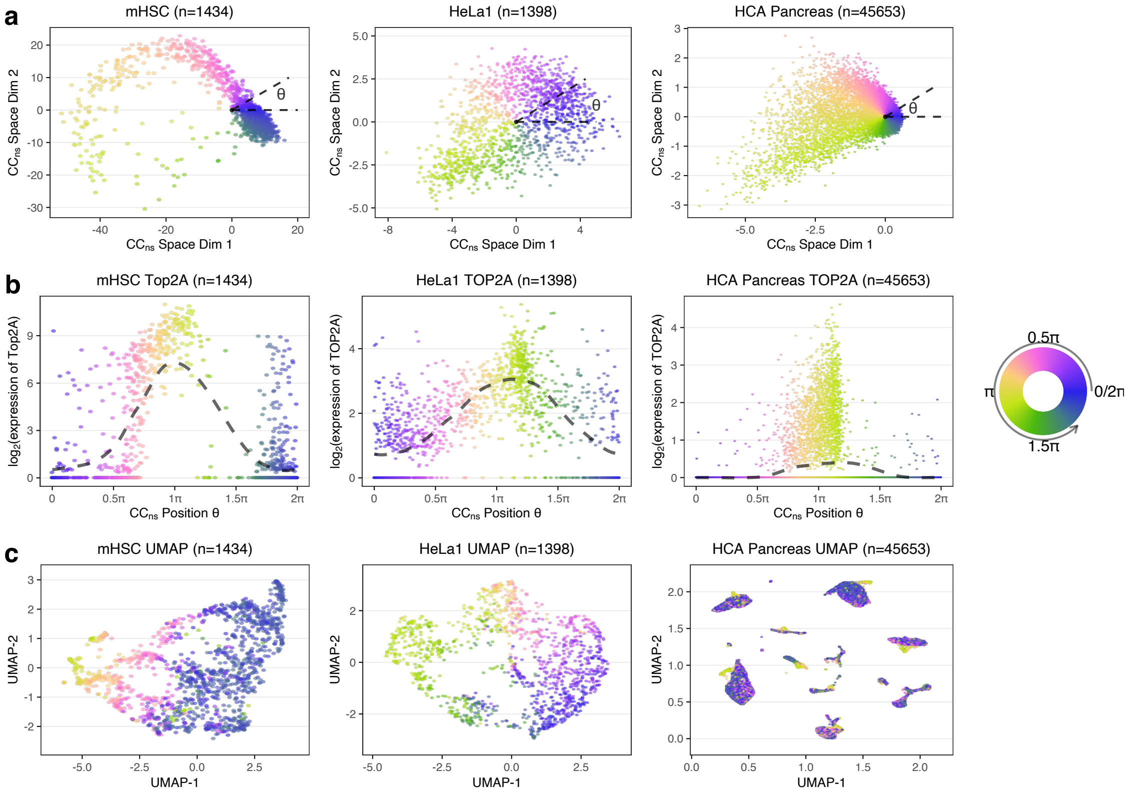

Fig. S9. A pre-learned rotation matrix learned from proliferating cortical neurospheres enables cell cycle position estimation in other proliferating datasets. This figure include three other datasets in addition to the four datasets in Figure 4. **(a)** Different datasets (mouse hematopoietic stem cell, Hela set 1 and human fetal pancreas dataset.) projected into the cell cycle embedding defined by the cortical neurosphere dataset. Cell cycle position θ is estimated using polar angle. **(b)** Inferred expression dynamics of *Top2A*(or *TOP2A* for human), with a periodic loess line (Methods). **(c)** UMAP embeddings of top variable genes. All the cells are colored by cell cycle position using a circular color scale.

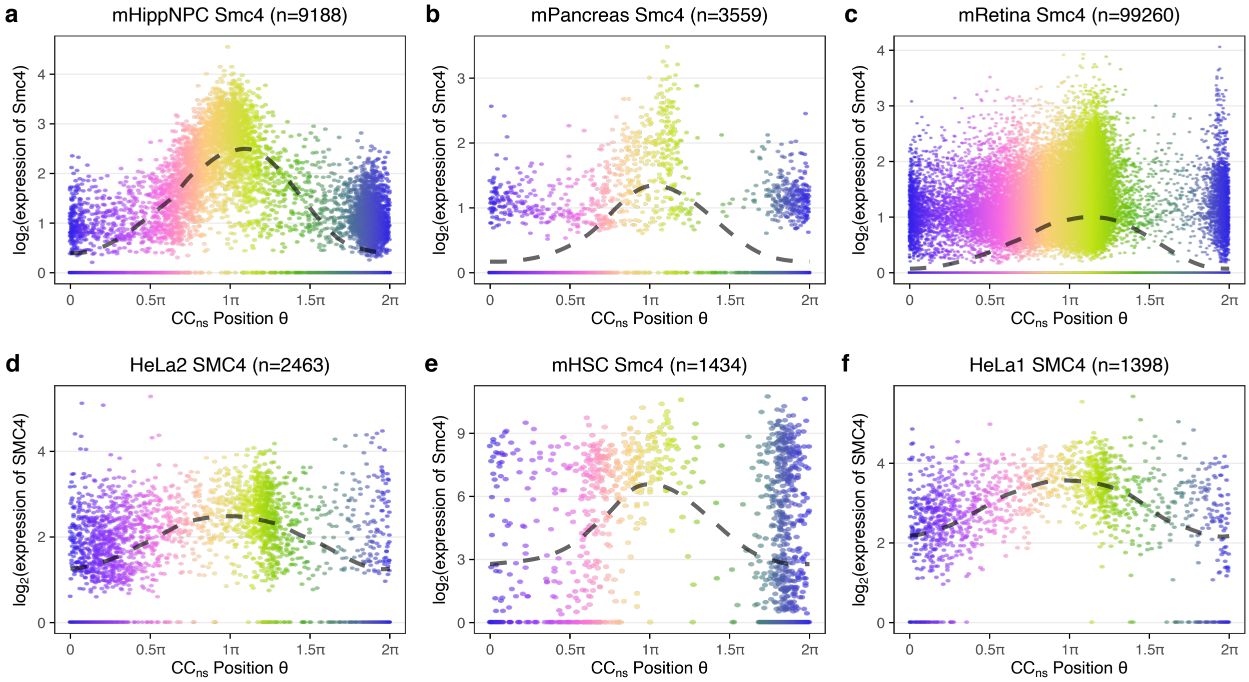

Fig. S10. The dynamics of *Smc4* expression over cell cycle position θ. Inferred expression dynamics of *Smc4*(or *SMC4* for human) over cell cycle position inferred using cortical neurospheres reference, with a periodic loess line (Methods) for **(a)** hippocampal NPCs, **(b)** mouse pancreas, **(c)** mouse retina, **(d)** Hela set 2, **(e)** mouse hematopoietic stem cell, and **(f)** Hela set 1 data. These data are the same data used in Figure 4 and Fig. S9.

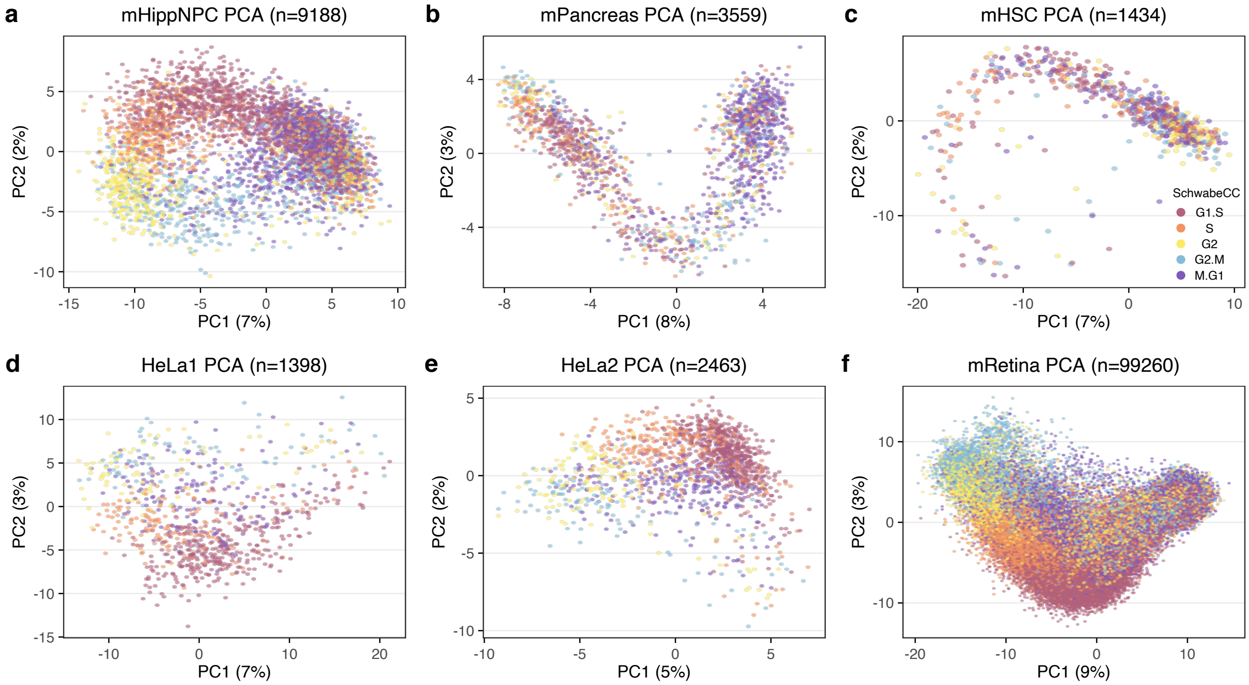

Fig. S11. The top 2 PCs of GO cell cycle genes. The figure consists top 2 PCs of PCA performed on GO cell cycle genes of each dataset. They serve as companion figures to Figure 4 and Fig. S9. Note that the cell cycle progression is hidden by direct PCA on datasets with higher heterogeneity, such as mPancreas and mRetina dataset, while cell cycle progression is visible in other datasets.

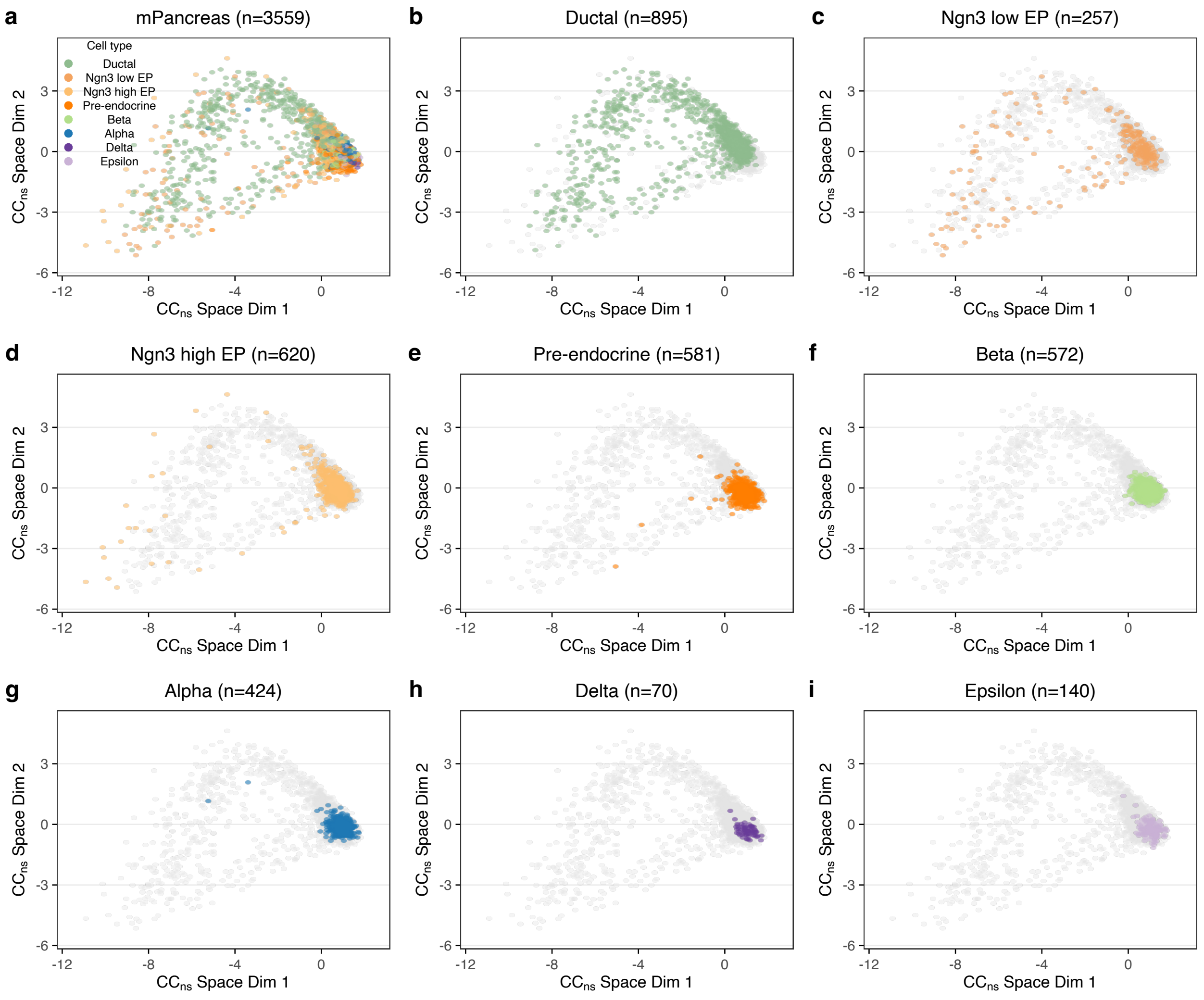

Fig. S12. A pre-learned rotation matrix learned from proliferating cortical neurospheres enables cell cycle position estimation in the mouse developing pancreas data. (a) The mouse developing pancreas data projected into the cell cycle embedding defined by the cortical neurosphere dataset. **(b-i)** As (a), but we only highlight one cell type in each panel, with the rest cells as background grey points. Note that the multi potency is decreasing from the ductal cells to more differentiated secretory cell types, which are not cycling.

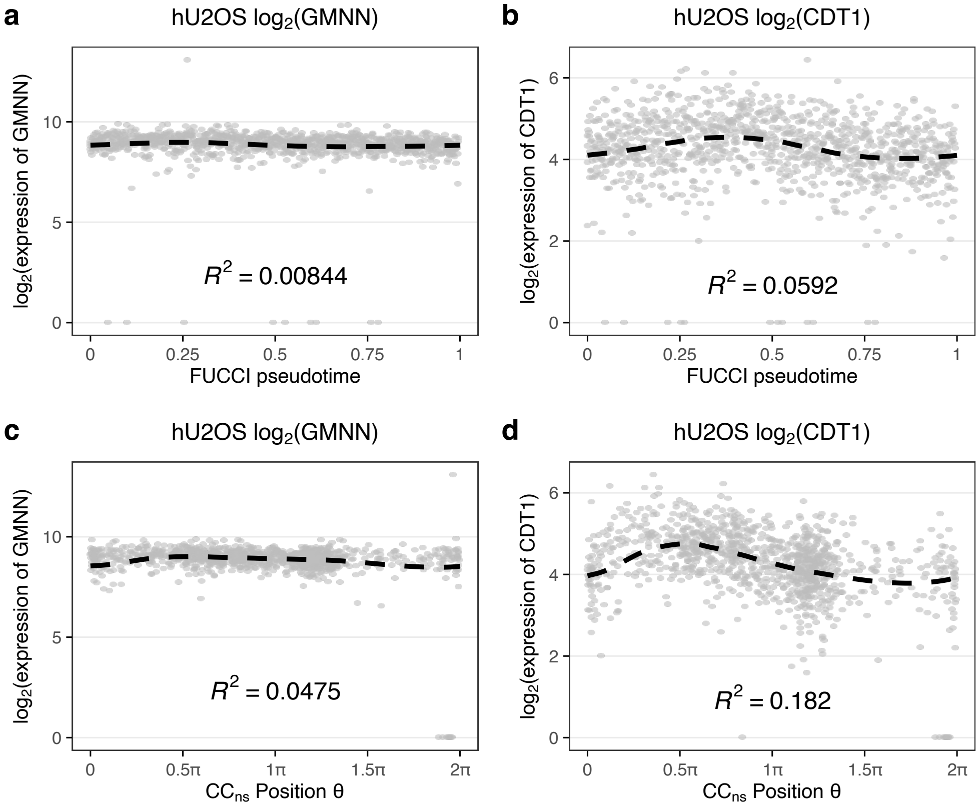

Fig. S13. Expression dynamics of GMNN and CDT1 on FUCCI pseudotime and tricycle position of hU2OS data. All data is from the hu2OS dataset. **(a,b)** The gene expression of (a) GMNN and (b) CDT1 as a function of FUCCI pseudotime derived using imaging of the protein levels. **(c,d)** Like (a,b) but as a function of tricycle cell cycle position inferred using scRNA data. Note that GMNN has constant expression over the cell cycle while CDT1 oscillates. This strongly suggests that the protein level of GMNN is regulated post transcriptionally.

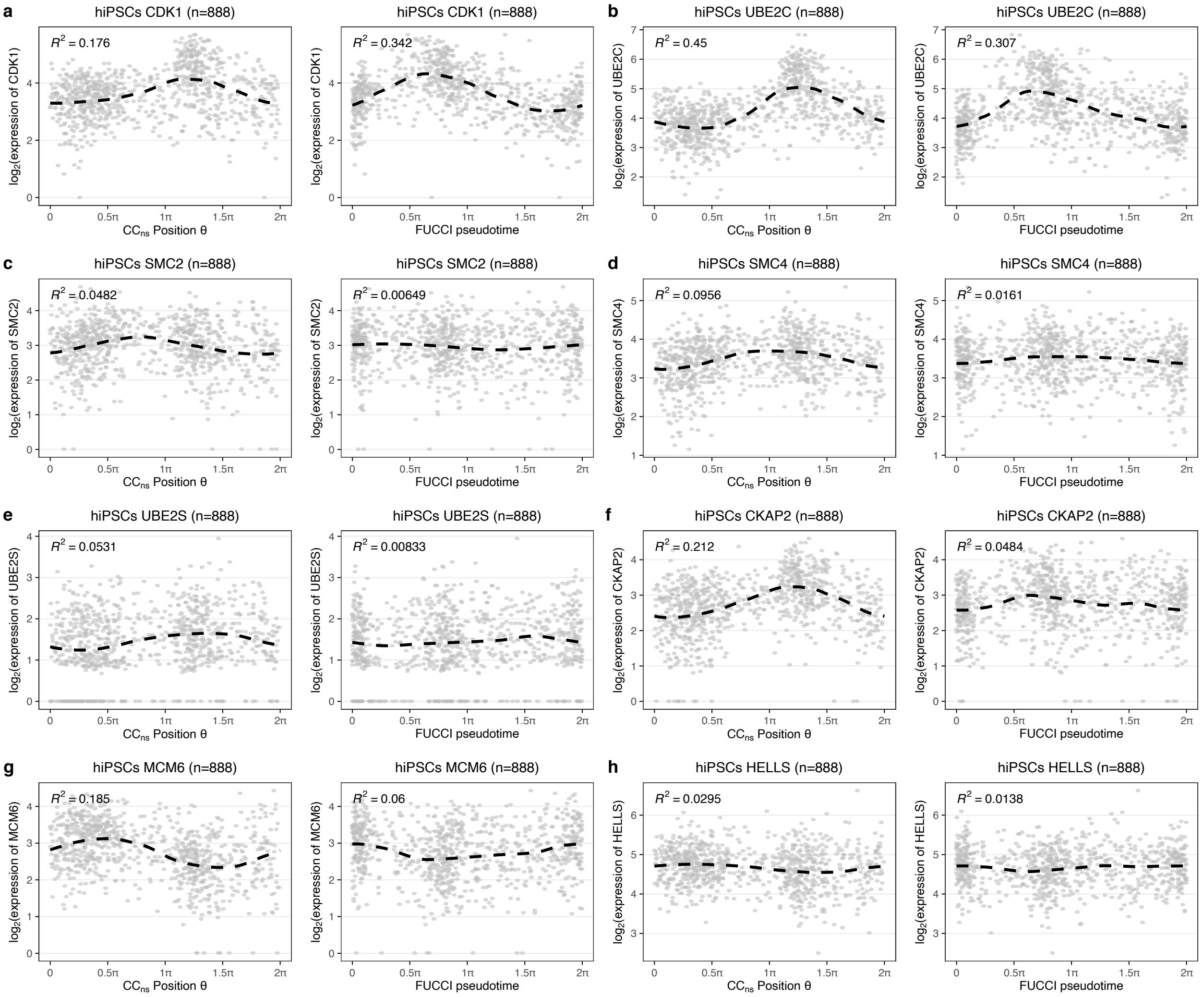

Fig. S14. Expression dynamics of selected cell cycle genes of hiPSCs dataset. Similar to Figure 5e,f, but now we show more cell cycle related genes. In each panel, the left sub-panel shows the expression of the gene over tricycle cell cycle position θ using mNeurosphere reference, and the right sub-panel over the FUCCI pseudotime inferred by Hsiao et al. (2020). Periodic loess lines and R^2 are added for each sub-panel (Methods).

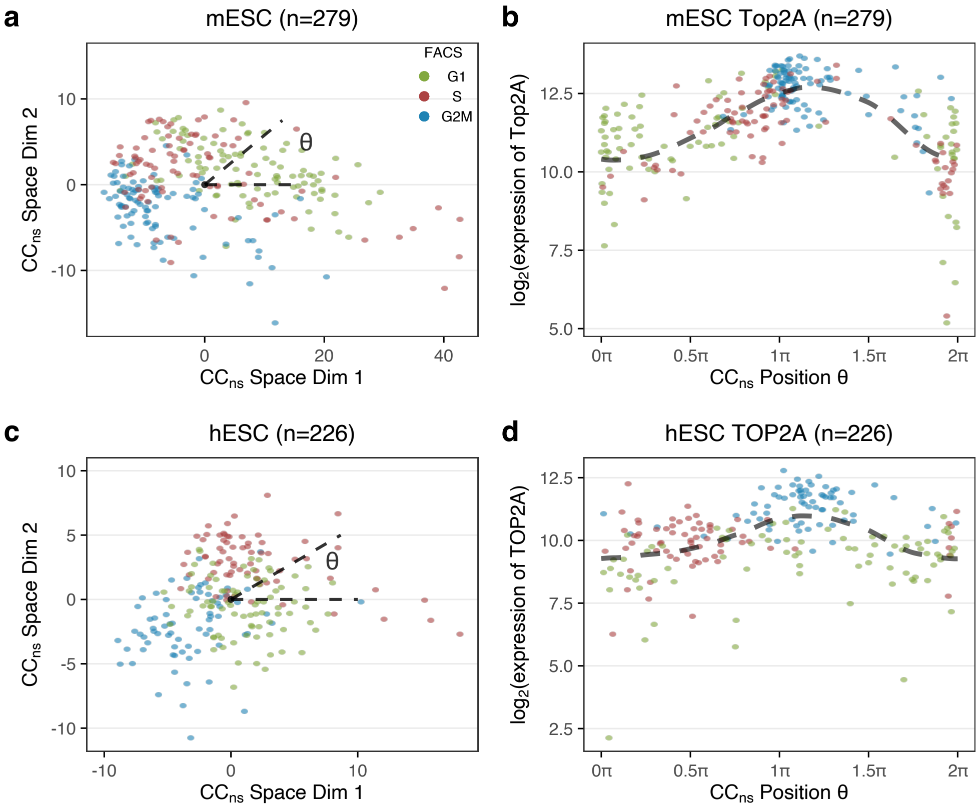

Fig. S15. Evaluation of tricycle on FACS datasets (a-b) Data from Buettner et al. (2015). **(a)** The data is projected to the cell cycle embedding defined by the cortical neurosphere dataset. Cells are colored by FACS labels. **(b)** Expression dynamics of *Top2A* with a periodic loess line using tricycle cell cycle position estimated by projection in (a). **(c-d)** Similar to (a,b), but for data from Leng et al. (2015).

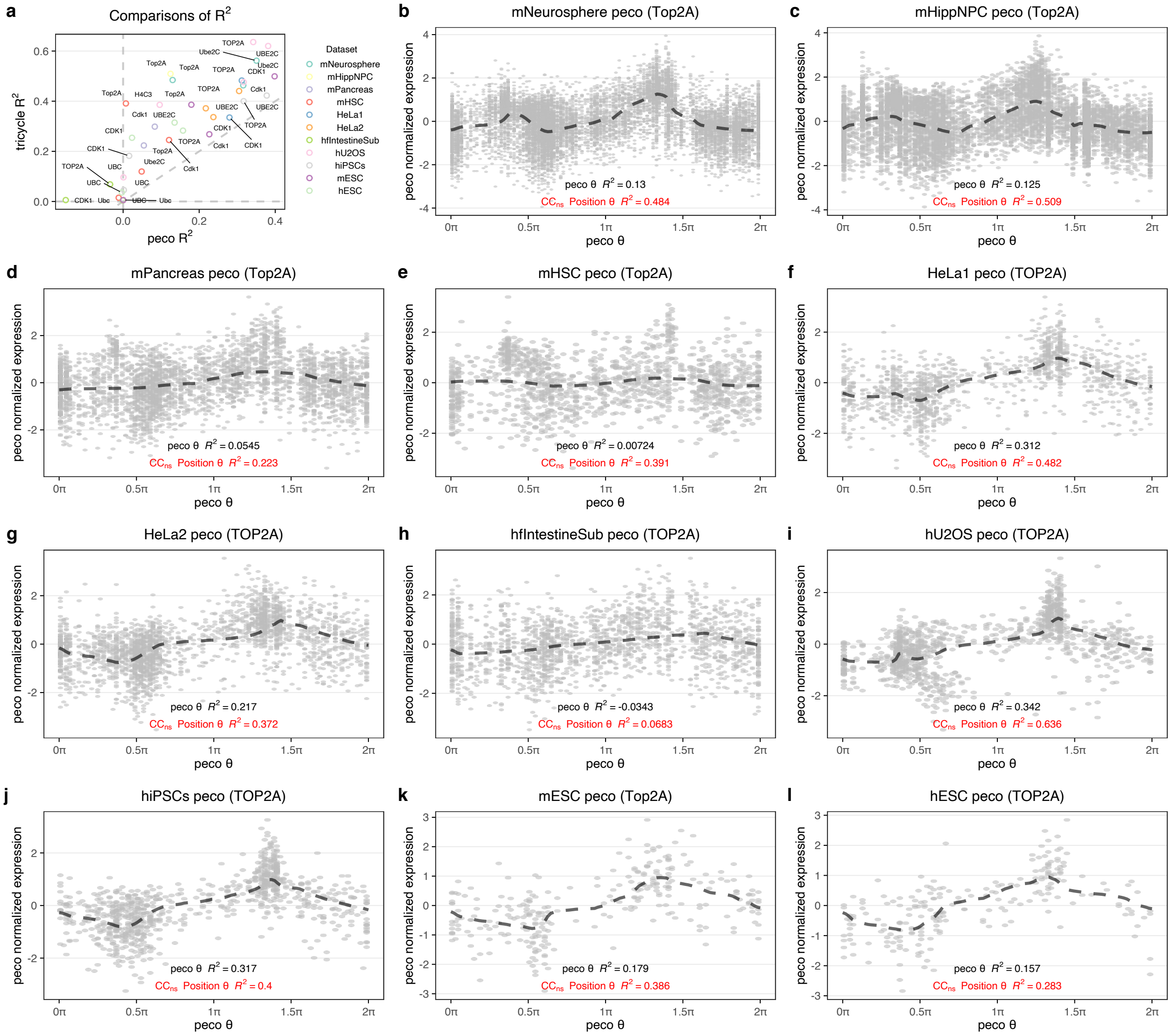

Fig. S16. Expression dynamics of cell cycle genes on peco cell cycle position. We run peco on all dataset described in Table 1, except mRetina and human fetal tissues. mRetina data has too many cells, and for human fetal tissues, we only use a subset of random 2000 cells from intestine data (hfIntestineSub). For each data, the R^2 of expression dynamics of *Cdk1*, *Top2A*, *Ube2C* and *H4c3*, as long as they exist in the target dataset, over peco inferred θ are computed. **(a)** Comparison of R^2 of peco inferred θ and R^2 of loess line on tricycle inferred θ using mNeurosphere reference for all datasets and genes mentioned above. **(b-l)** Expression dynamics of *Top2A* (*TOP2A* for human) over peco inferred θ for each dataset. Note that in these panels, the y-axis represents the peco normalized expression values, as peco has its own normalization requirement. We annotate the each panel with R^2 of loess line calculated on peco inferred θ and R^2 of loess line on tricycle inferred θ using mNeurosphere reference (although we have not plotted out the expression dynamics over tricycle inferred θ). Across all datasets and genes, the tricycle inferred θs have greater R^2 to peco θ, and are highlighted as red.

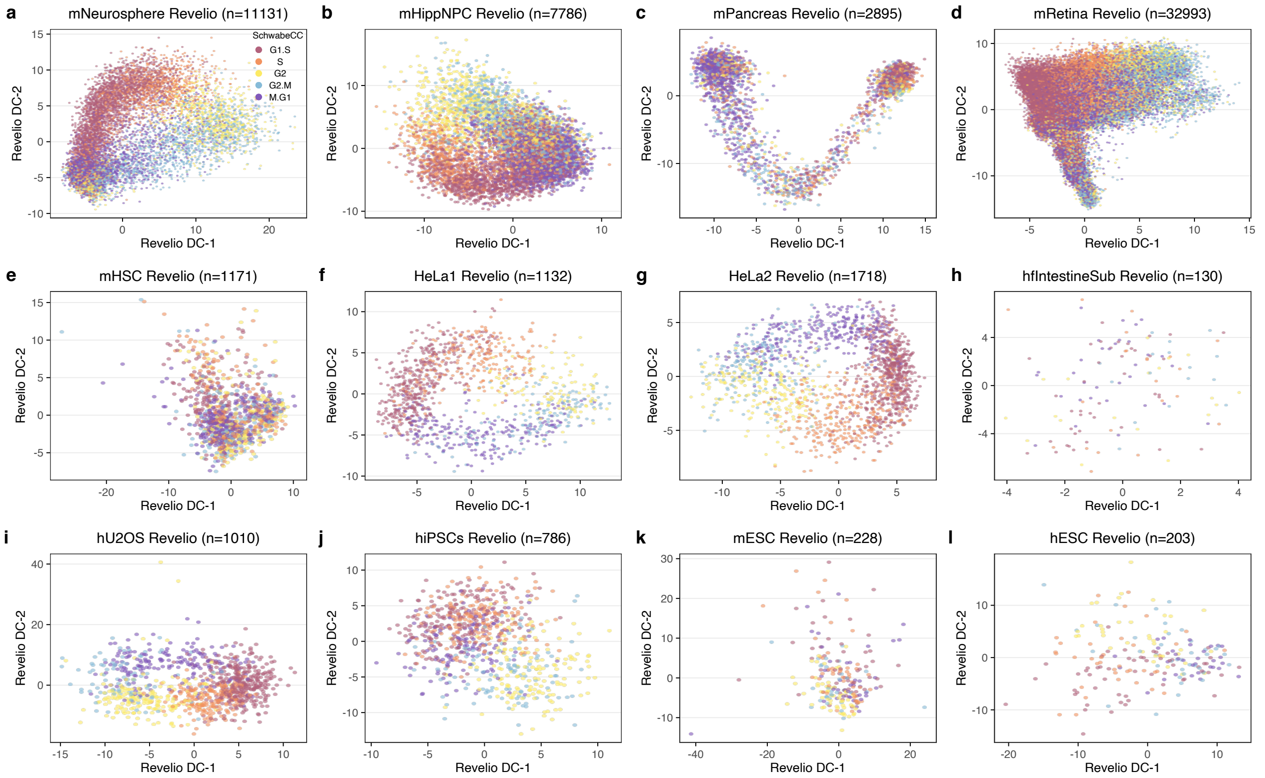

Fig. S17. Cell cycle embeddings by Revelio. The cell cycle embedding produced by Revelio for each data. Cells are colored by 5 stage cell cycle representation, inferred using the original Schwabe method (Schwabe et al., 2020) as implemented in the Revelio package. Note that all cells without a valid stage assignment (assigned to "NA") are removed by the functions in Revelio package.

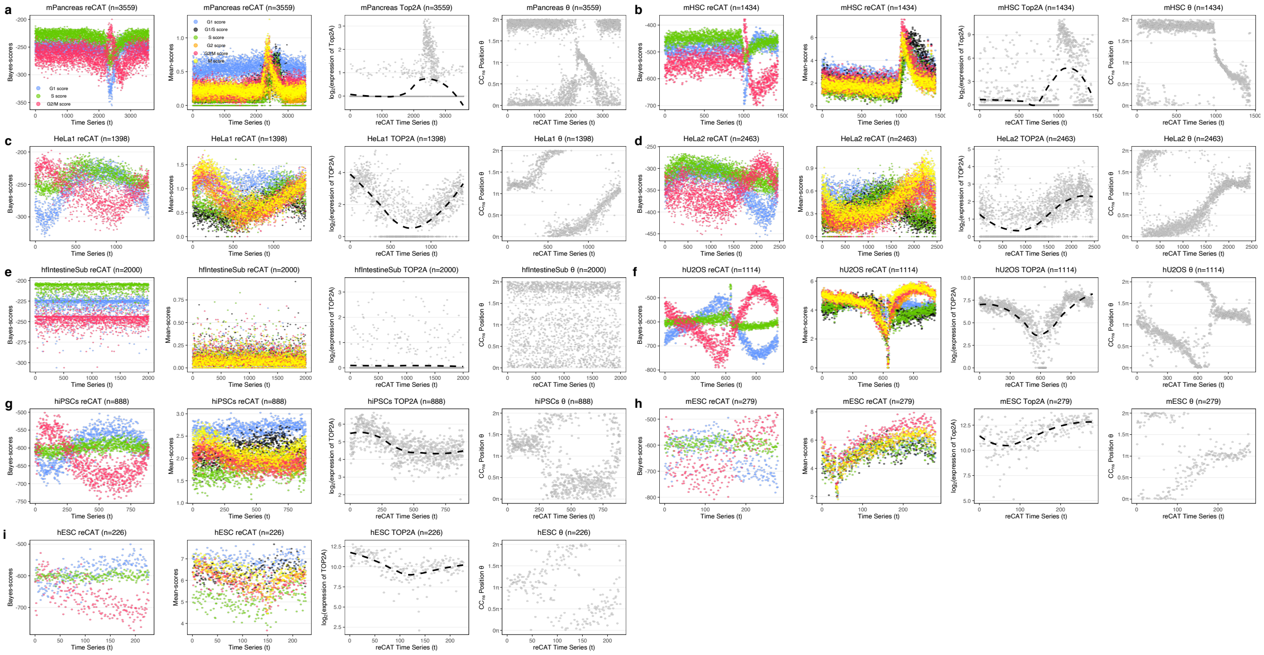

Fig. S18. Cell cycle stage and order estimations by reCAT. Panels show the cell cycle stage scores and cell orders estimated by reCAT for **(a)** mPancreas, **(b)** mHSC, **(c)** HeLa set 1, **(d)** HeLa set 2, **(e)** hfIntestineSub, **(f)** hU2OS, **(g)** hiPSCs, **(h)** mESC, and **(i)** hESC data. Each data point is a cell. For each data, the first sub-panel shows the Bayes scores for G1, S, and G2/M stage over the estimated cell orders(time series t). For each cell, there will be three scores (data points) colored by stage. The second sub-panel shows the mean scores for G1, G1/S, S, G2, G2/M, and M stage over the estimated cell orders. For each cell, there will be six scores (data points) colored by stage. The third sub-panel is the expression dynamic of *Top2A*(or *TOP2A* for human) over reCAT estimated cell orders. Note that although reCAT package provide function to assign cell cycle stage, it requires manual input cutoff for Bayes scores. It is unrealistic for us to pick some appropriate cutoffs for most of the datasets presented here. For example, for mPancreas data in (a), we cannot decide which region has the consistent G1 scores. The last sub-panels compares tricycle cell cycle position using mNeurosphere reference and reCAT cell orders.

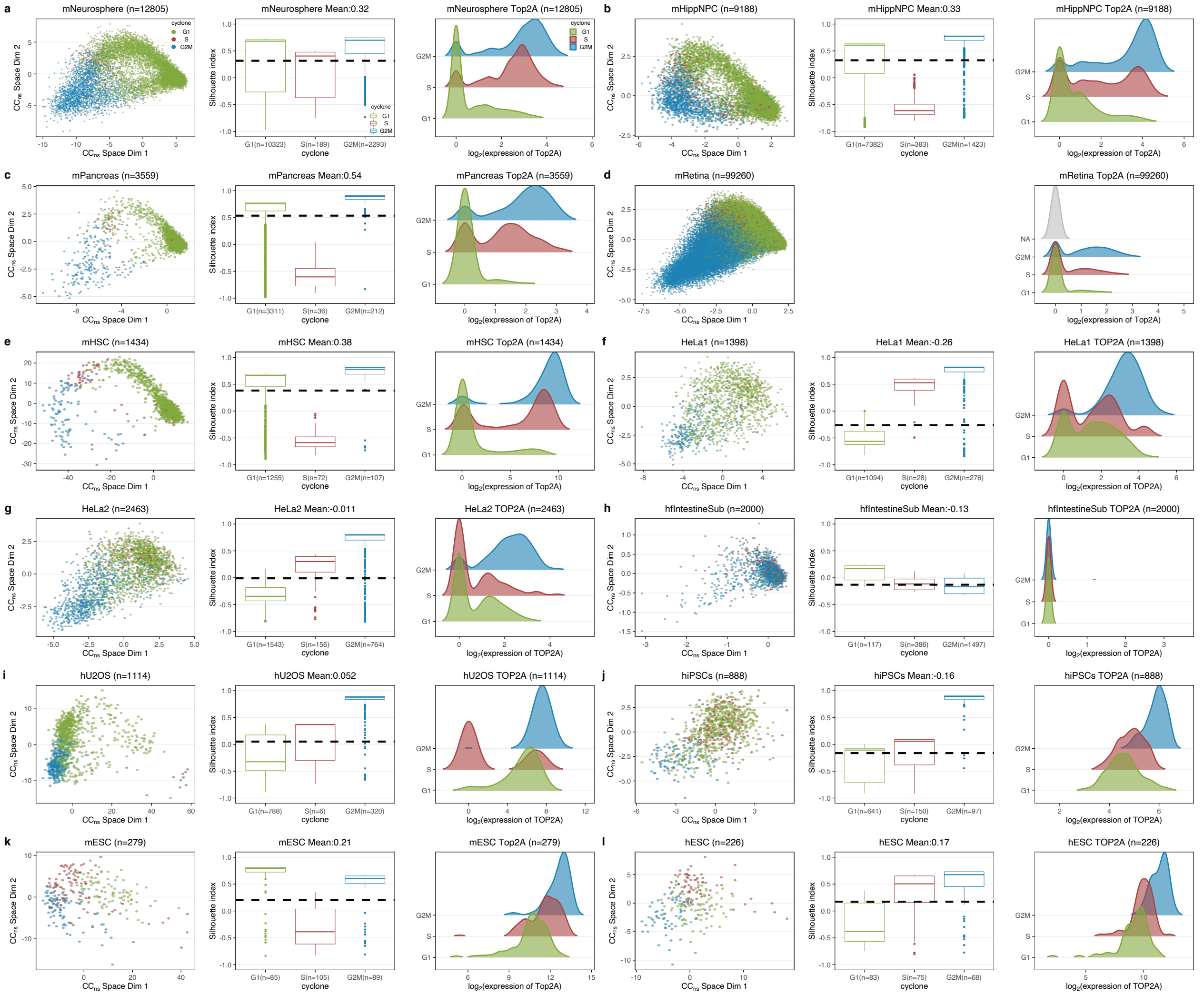

Fig. S19. Comparison between cyclone assigned stages and tricycle cell cycle position using mNeurosphere reference. Each panel describe one data, specifically for **(a)** mNeurosphere, **(c)** mHippNPC, **(c)** mPancreas, **(d)** mRetina, **(e)** mHSC, **(f)** HeLa set 1, **(g)** HeLa set 2, **(h)** hfIntestineSub, **(i)** hU2OS, **(j)** hiPSCs, **(k)** mESC, **(l)** hESC data. For each data, the first sub-panel shows the cell cycle embedding projection by mNeurosphere reference, and each point is a cell, colored by cyclone inferred cell cycle stage. The second sub-panel shows silhouette index computed using angular separation distance of tricycle cell cycle position θ estimated using mNeurosphere reference (Methods), stratified by cyclone inferred cell cycle stage. The mean silhouette index across all cells is given in the title. Boxes indicate 25th and 75th percentiles. Whiskers extend to the largest values no further than 1.5 $\times$ interquartile range (IQR) from these percentiles. For mRetina data, the pairwise distance matrix is too big to substantiate, so we could not compute silhouette index. The third sub-panel shows the marginal density of *Top2A*(or *TOP2A* for human) expression conditioned on cyclone cell cycle stage.

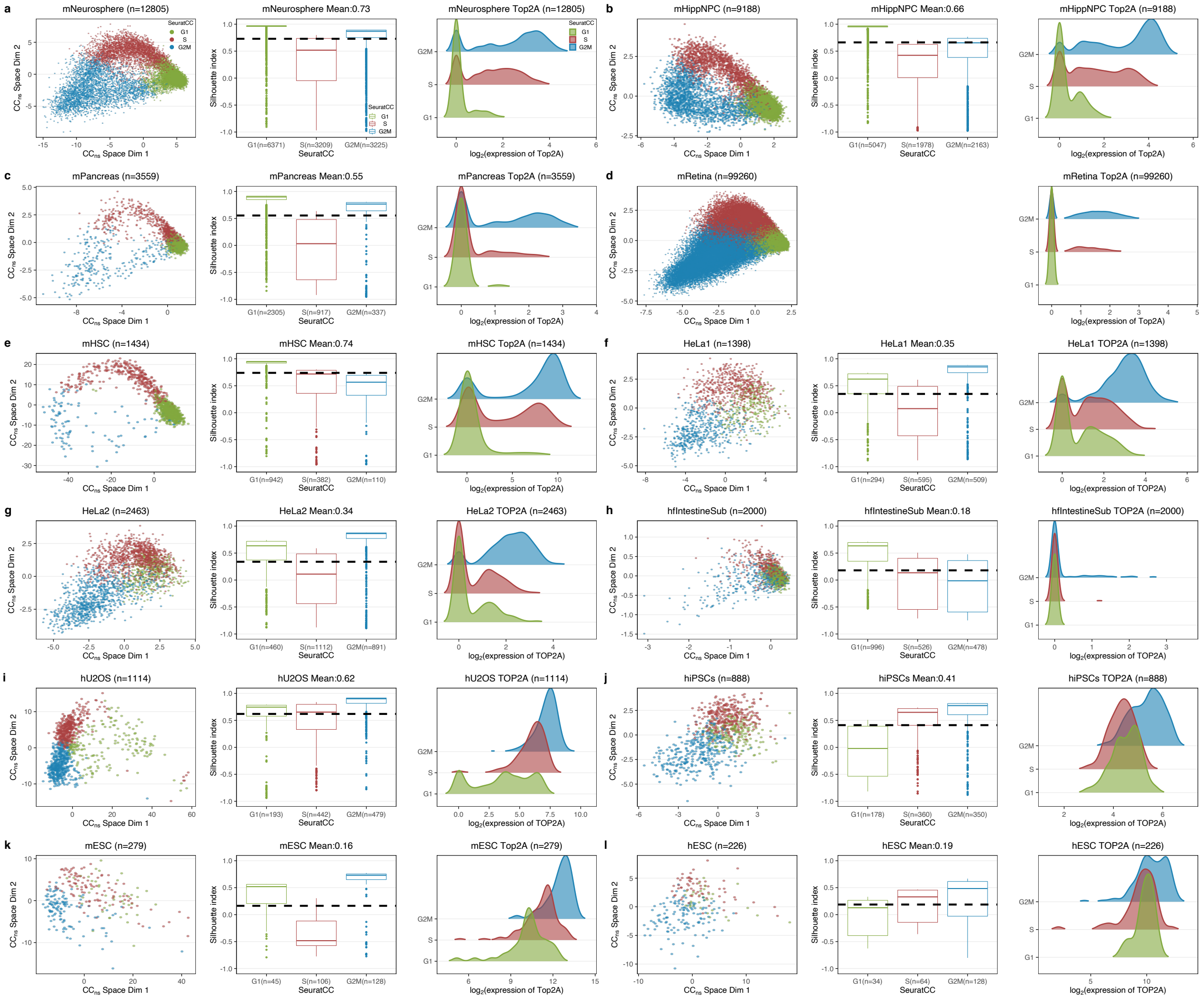

Fig. S20. Comparison between Seurat assigned stages and tricycle cell cycle position using mNeurosphere reference. Each panel describe one data, specifically for **(a)** mNeurosphere, **(c)** mHippNPC, **(c)** mPancreas, **(d)** mRetina, **(e)** mHSC, **(f)** HeLa set 1, **(g)** HeLa set 2, **(h)** hfIntestineSub, **(i)** hU2OS, **(j)** hiPSCs, **(k)** mESC, **(l)** hESC data. For each data, the first sub-panel shows the cell cycle embedding projection by mNeurosphere reference, and each point is a cell, colored by Seurat inferred cell cycle stage. The second sub-panel shows silhouette index computed using angular separation distance of tricycle cell cycle position θ estimated using mNeurosphere reference (Methods), stratified by Seurat inferred cell cycle stage. The mean silhouette index across all cells is given in the title. Boxes indicate 25th and 75th percentiles. Whiskers extend to the largest values no further than 1.5·IQR from these percentiles. For mRetina data, the pairwise distance matrix is too big to substantiate, so we could not compute silhouette index. The third sub-panel shows the marginal density of *Top2A*(or *TOP2A* for human) expression conditioned on Seurat cell cycle stage.

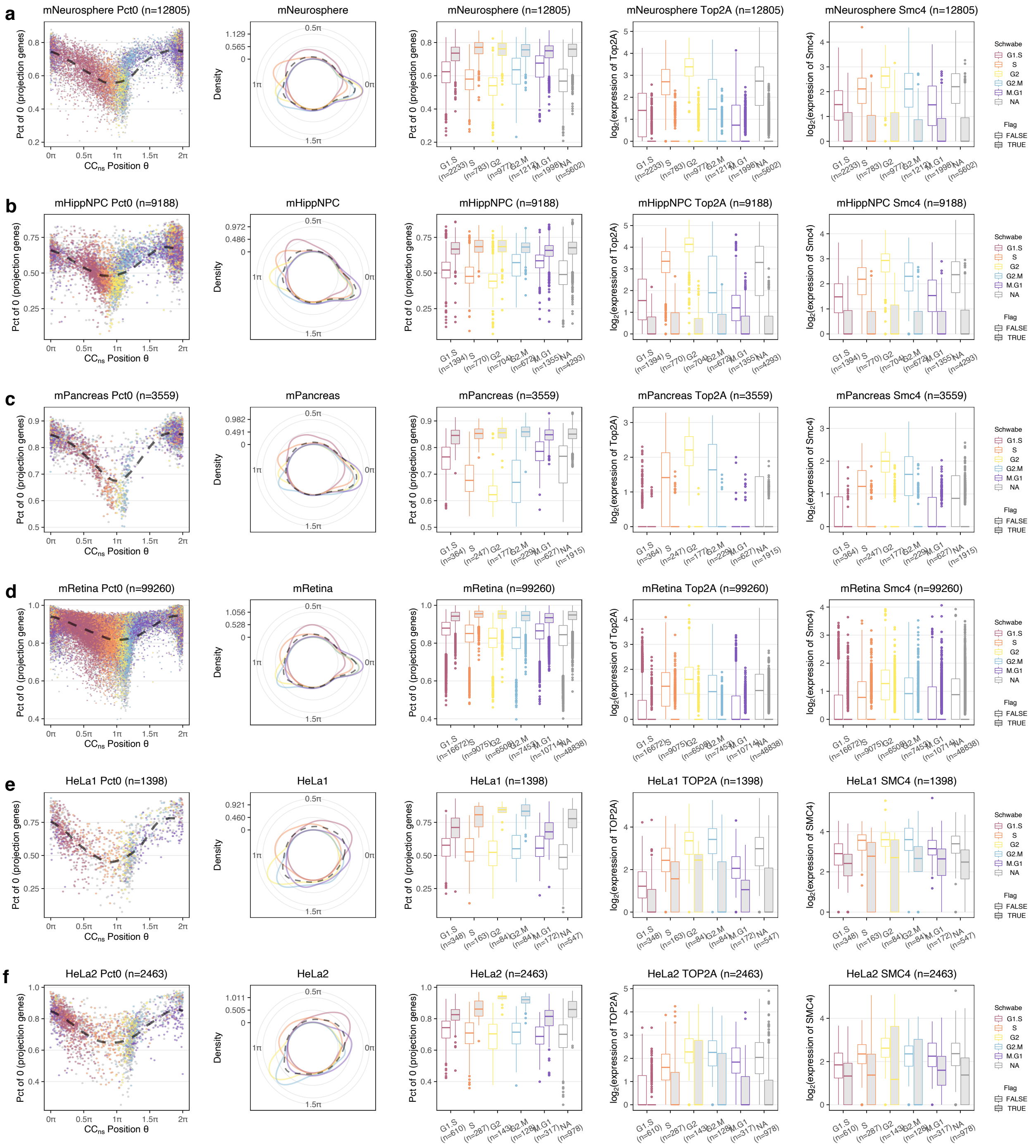

Fig. S21. Comparison between SchwabeCC 5 stage assignments and tricycle cell cycle position using mNeurosphere reference. See next page for caption.

Fig. S21. (Continued). Each row or panel contains analysis for a dataset, specifically **(a)** for mNeurosphere, **(b)** for mHippNPC, **(c)** for mPancreas, **(d)** for mRetina, **(e)** for HeLa set 1, **(f)** for HeLa set 2 data. For each data, the first sub-panel shows the dynamics of percentage of non-expressed genes over all overlapped genes with mNeurosphere projection matrix (number of genes with 0 expression divided by the number overlapped genes with mNeurosphere projection matrix) w.r.t. tricycle cell cycle position θ using mNeurosphere reference. Cells are colored by 5 stage assignment. The second panel shows the marginal density of tricycle cell cycle position θ conditioned on 5 stage assignments using von Mises kernel on polar coordinate system. The third sub-panel shows the percentage of non-expressed genes over all overlapped genes with mNeurosphere projection matrix conditioned on 5 stages assignment and whether cells appear in the G1/G0 cluster - $\theta < 0.25\pi$ or $\theta > 1.5\pi$ as boxplots. The forth and the last sub-panel show the expression of *Top2A* and *Smc4* conditioned on 5 stages assignment and whether cells appear in the G1/G0 cluster. Boxes indicate 25th and 75th percentiles. Whiskers extend to the largest values no further than 1.5·IQR from these percentiles.

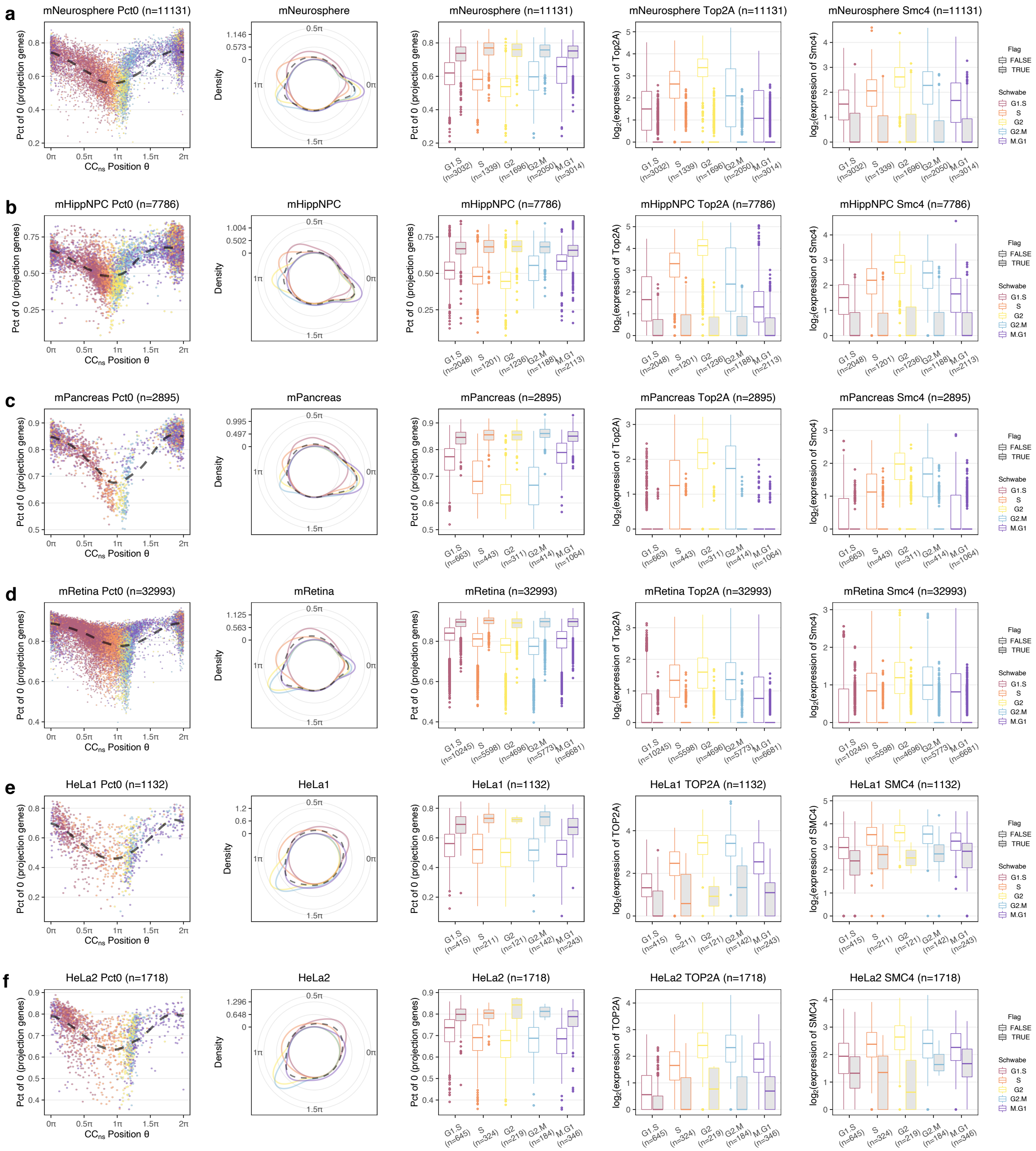

Fig. S22. Comparison between original SchwabeCC 5 stage assignments and tricycle cell cycle position using mNeurosphere reference. This figure shows the exact same data and comparisons as in Fig. S21, but now we use the original SchwabeCC method as implemented in the Revelio package (Schwabe et al., 2020). Note that the number of cells in each dataset is decreased as any cell without a valid stage assignment (assigned to ”NA”) is removed by the functions in Revelio package.

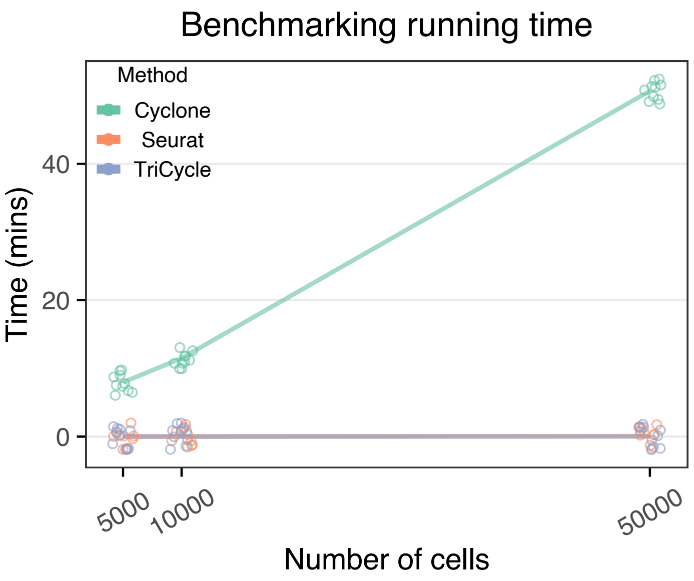

Fig. S23. Running time comparisons between cyclone, Seurat, and tricycle cell cycle inference We record the elapsing time for each method when running them on 10 random subsets of mRetina data with 5000, 10000, and 50000 cells. For cyclone and Seurat, the time is recorded for the cell cycle stage assignment function. For tricycle, the time is recorded for cell cycle position estimation using mNeurosphere reference. Note that we add jitters to the data points to avoid excessive overlaps.

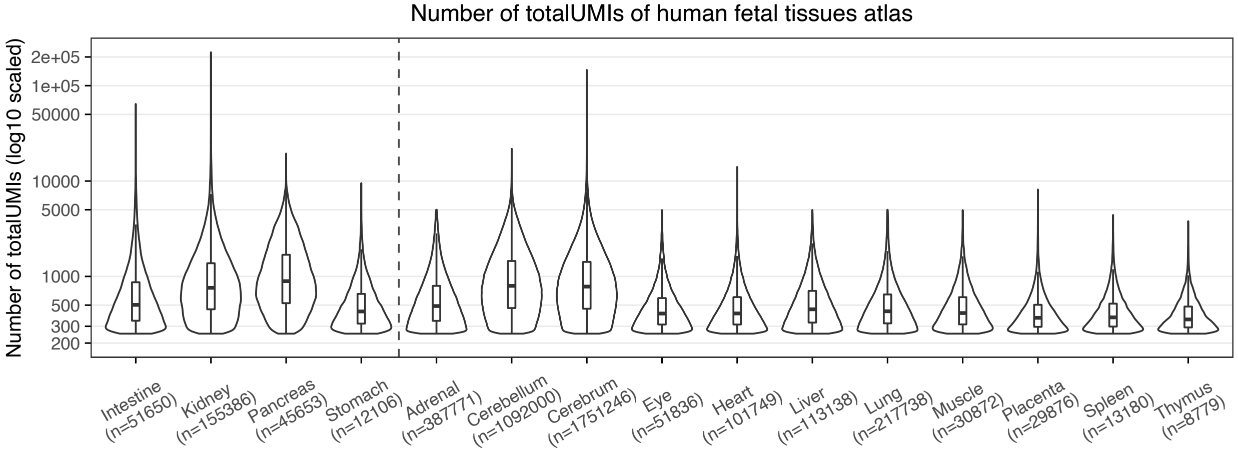

Fig. S24. TotalUMIs of human fetal atlas. For each tissue type of the human fetal atlas data (Cao et al., 2020), we show the total UMIs of a cell. The dashed line separates 4 single-cell profiled tissues with 11 single-nuclei profiled tissues. Boxes indicate 25th and 75th percentiles. Whiskers extend to the largest values no further than 1.5 × interquartile range (IQR) from these percentiles.

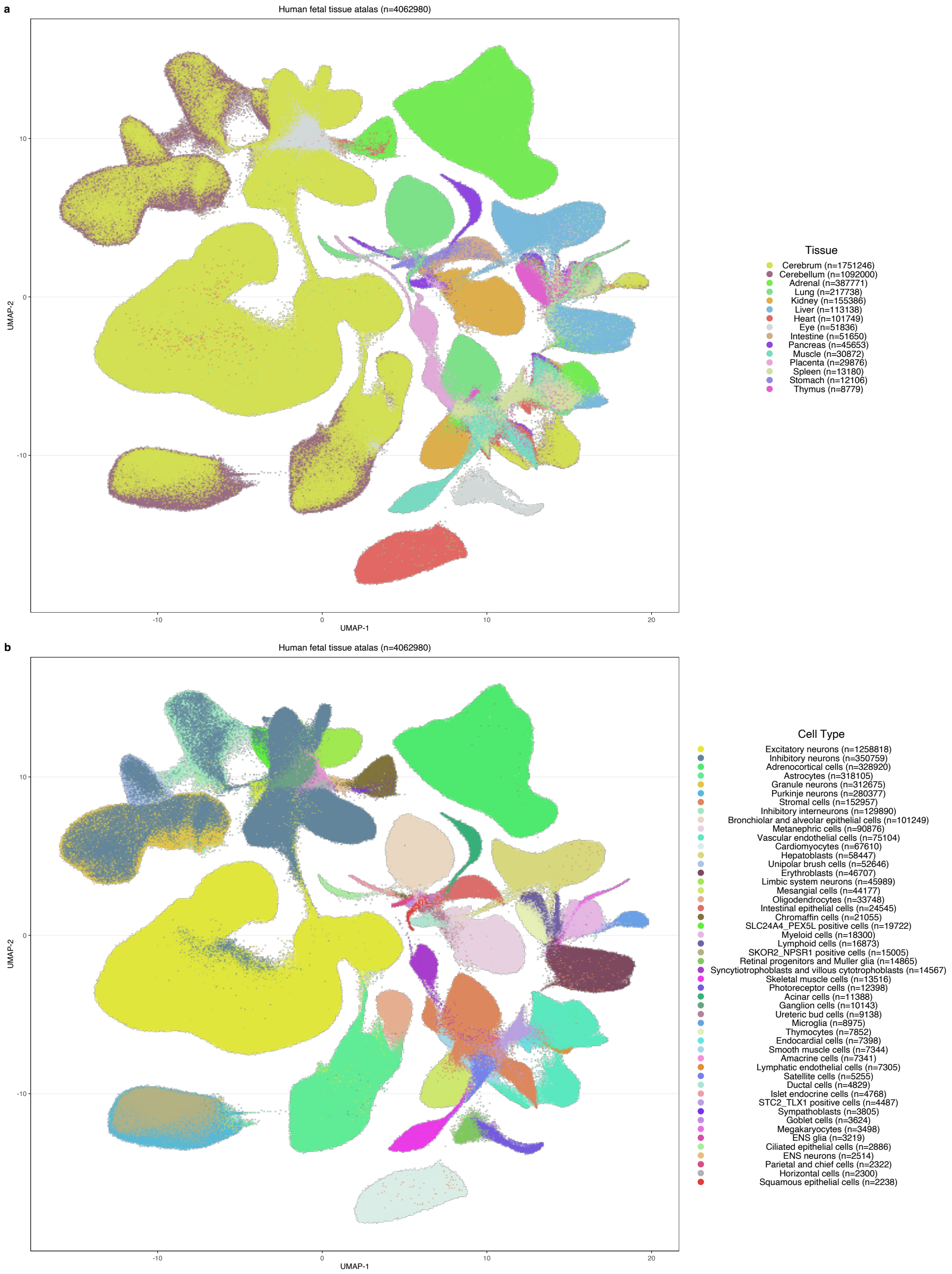

Fig. S25. Human fetal tissue atlas UMAP embeddings with all tissues Human fetal tissue atlas UMAP embeddings with all tissues, colored by **(a)** tissue and **(b)** cell type.

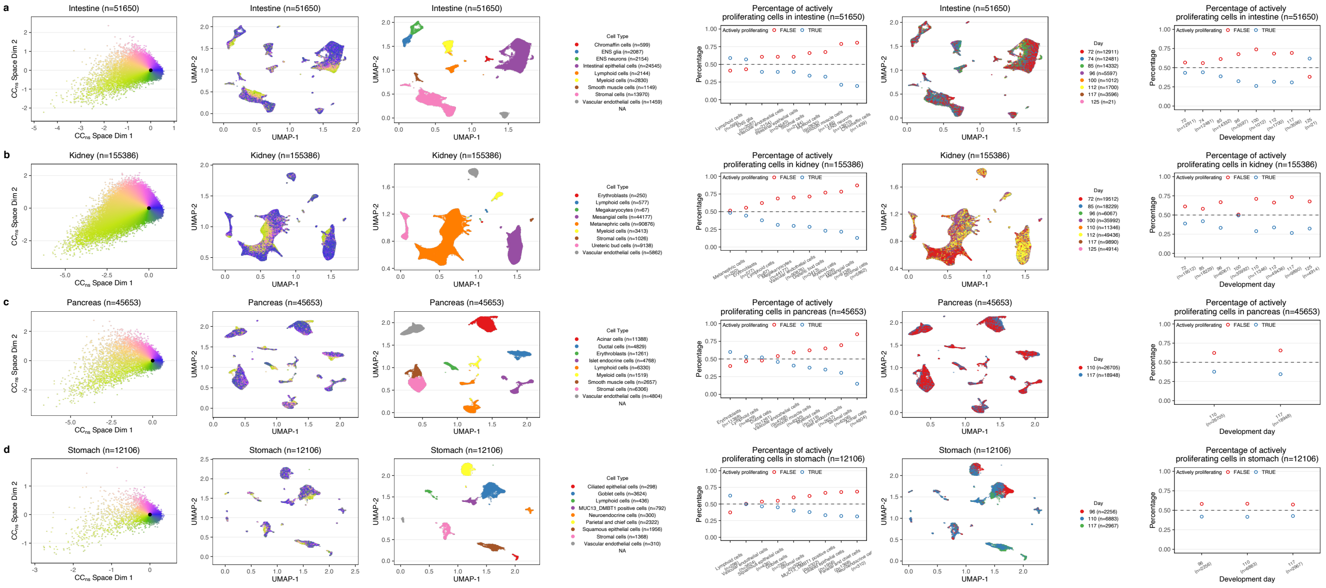

Fig. S26. Application of tricycle on 4 single-cell profiled human tissues We show one tissue type in each row/panel **(a)** intestine, **(b)** kidney, **(c)** pancreas, and **(d)** stomach. For each tissue, the cell cycle embedding using mNeurosphere reference is given in the first sub-panel, tissue-level UMAPs from Cao et al. (2020) colored by cell cycle position θ in the second sub-panel, tissue-level UMAPs from Cao et al. (2020) colored by cell type in the third sub-panel, percentage of actively proliferating cells for each cell type in decreasing order in the forth sub-panel, tissue-level UMAPs from Cao et al. (2020) colored by development days in the fifth sub-panel, and percentage of actively proliferating cells for each development day in the last sub-panel.

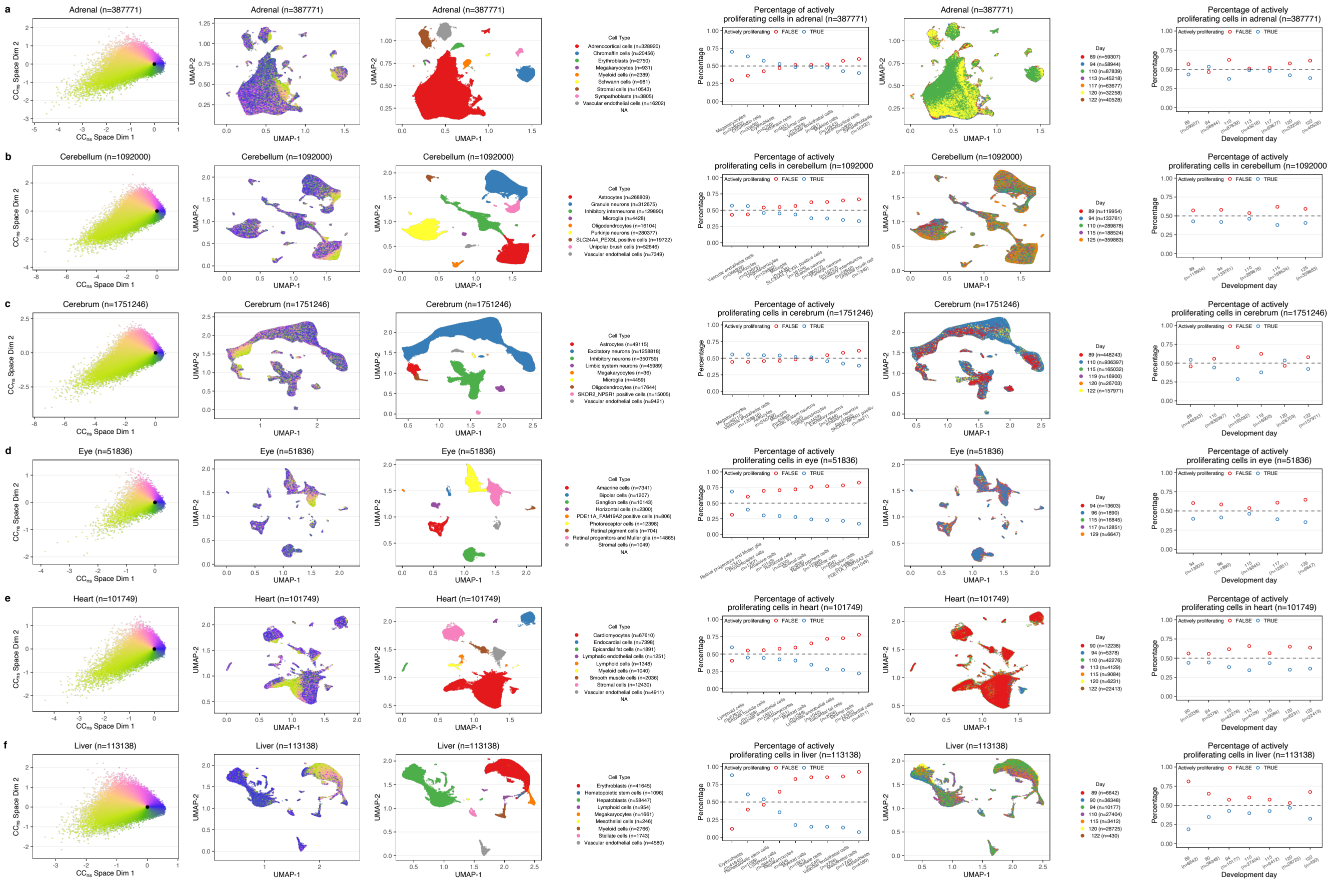

Fig. S27. Application of tricycle on 11 single-nuclei profiled human tissues Similar to Figure S26, but for 11 tissues with single-nuclei RNA profiled. We show one tissue type in each panel **(a)** adrenal, **(b)** cerebellum, **(c)** cerebrum, **(d)** eye, **(e)** heart, **(f)** liver, **(g)** lung, **(h)** muscle, **(i)** placenta, **(j)** spleen, and **(k)** thymus. For each tissue, the cell cycle embedding using mNeurosphere reference is given in the first sub-panel, tissue-level UMAPs from Cao et al. (2020) colored by cell cycle position θ in the second sub-panel, tissue-level UMAPs from Cao et al. (2020) colored by cell type in the third sub-panel, percentage of actively proliferating cells for each cell type in decreasing order in the forth sub-panel, tissue-level UMAPs from Cao et al. (2020) colored by development days in the fifth sub-panel, and percentage of actively proliferating cells for each development day in the last sub-panel.

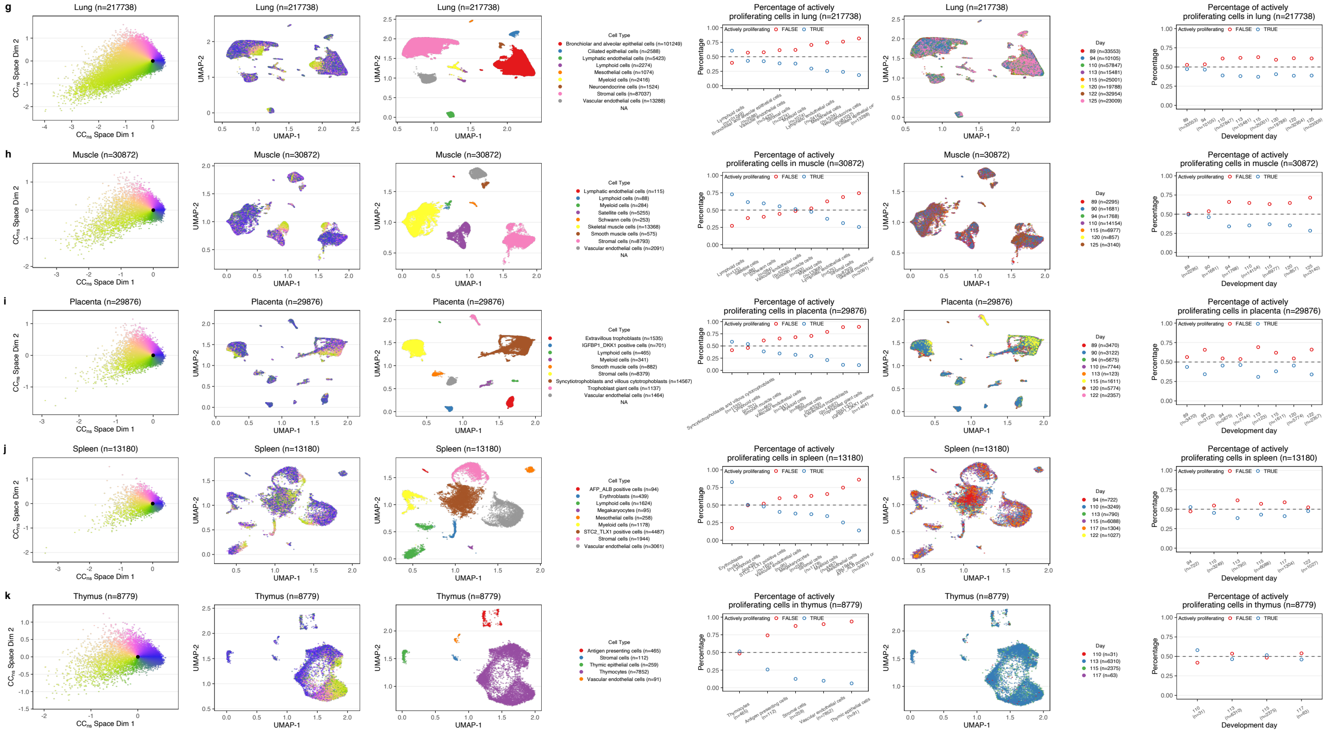

Fig. S27. (Continued) with **(g)** lung, **(h)** muscle, **(i)** placenta, **(j)** spleen, and **(k)** thymus.

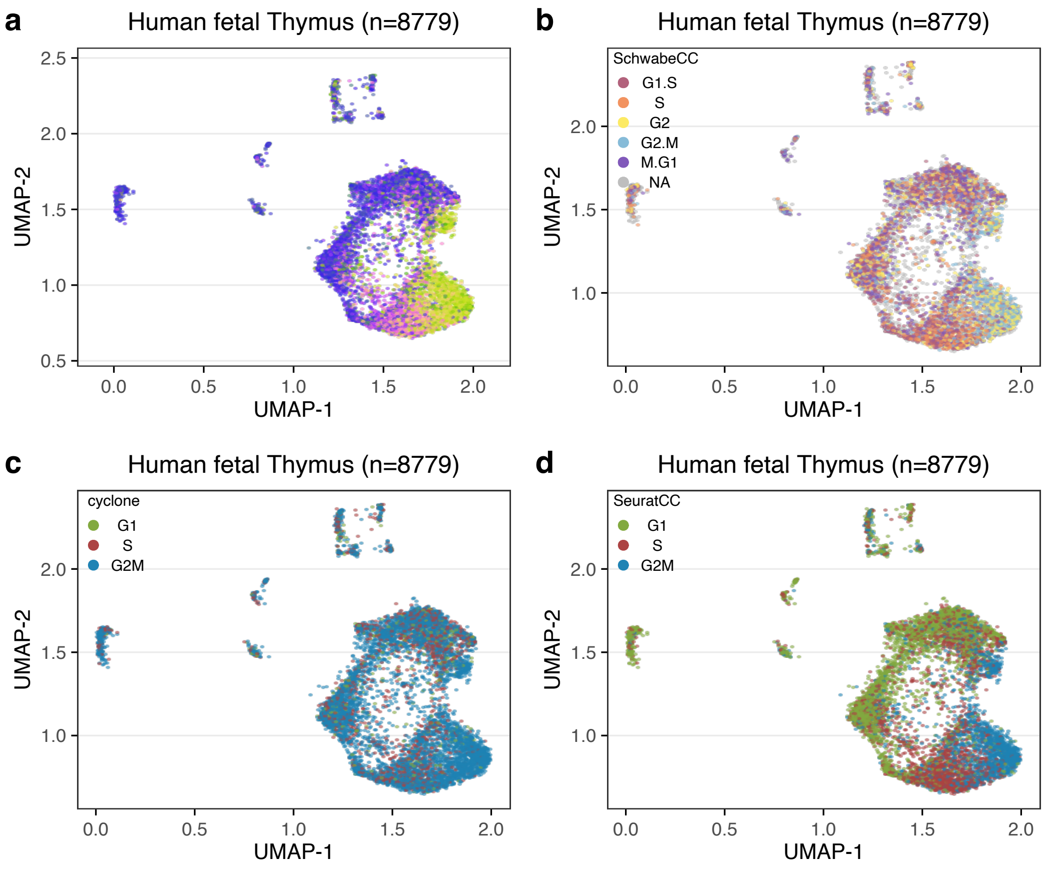

Fig. S28. Human fetal thymus UMAPs colored by cell cycle position or stage. (a) Same as Fig. S27k second sub-panel, which shows the UMAP embeddings of human fetal thymus, colored by cell cycle position θ. **(b)** Same UMAP embedding as in (a), but colored by 5 stage cell cycle representation, inferred using the SchwabeCC method from Schwabe et al. (2020). **(c)** Same UMAP embedding as in (a), but colored by 3 stage cell cycle representation, inferred by cyclone (Scialdone et al., 2015). **(d)** Same UMAP embedding as in (a), but colored by 3 stage cell cycle representation, inferred by Seurat (Stuart et al., 2019).

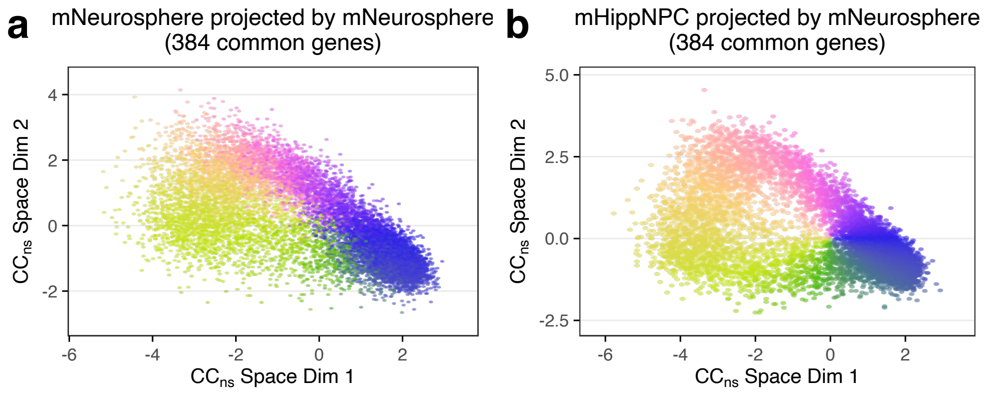

Fig. S29. Projection using the exact same genes on two datasets. This figure shows cell cycle embeddings for **(a)** mNeurosphere and **(b)** mHippNPC dataset using the subset mNeurosphere reference restricted to 384 genes existing in both datasets. Cells are colored by the cell cycle position θ.

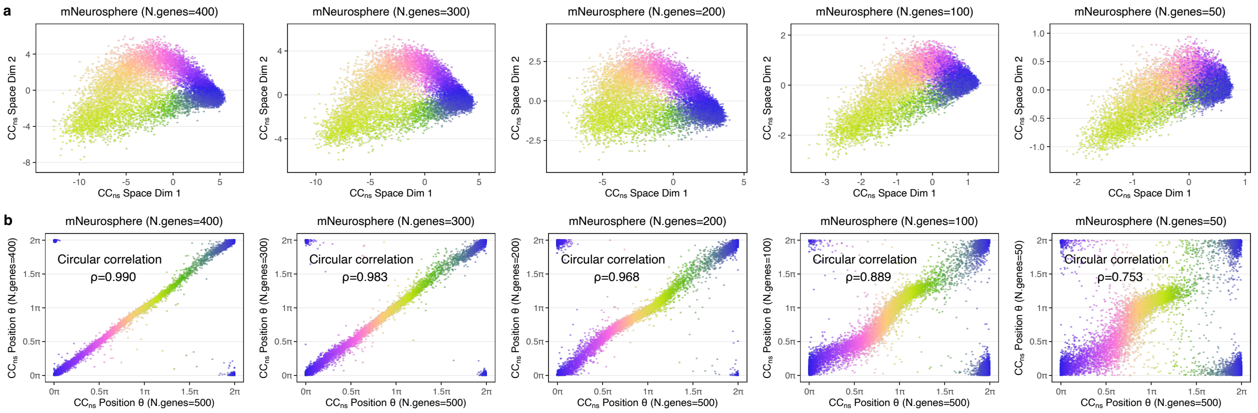

Fig. S30. Examples of mNeurosphere dataset projections with randomly sub-sampled projection matrices. Each column represents an example of a projection using the sub-sampled genes from original 500 projection genes. From left to the right, the numbers of genes are 400, 300, 200, 100, and 50. **(a)** The projected cell cycle embedding using the sub-sampled projection matrix. Cells are colored by cell cycle position inferred using all 500 projection genes. **(b)** Comparisons of cell cycle positions θ estimated using the full 500 projection matrix and using the sub-sampled projection matrix. The circular correlation ρ is given in the figure.

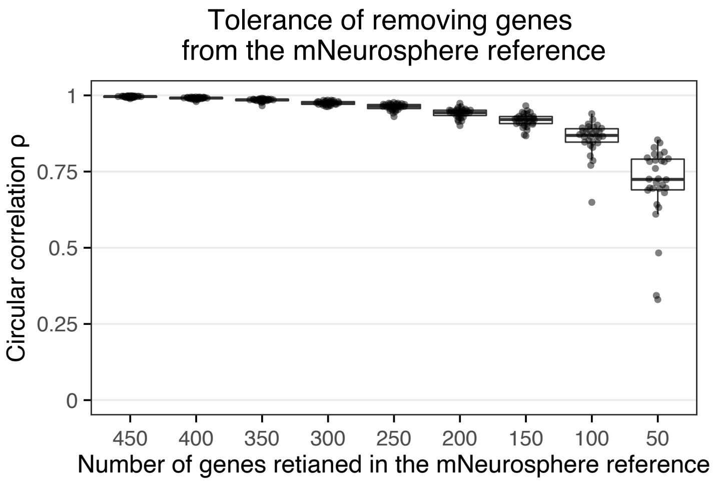

Fig. S31. Stability assessment with projection genes missing. This figure shows comprehensive assessments as complement to Fig. S30. For each target number of genes retained in the mNeurosphere reference matrix, we randomly sampled different genes 30 times. For each run, the circular correlation coefficient ρ was calculated between θ from projection using the full reference matrix and θ from projection using sub-sampled reference.

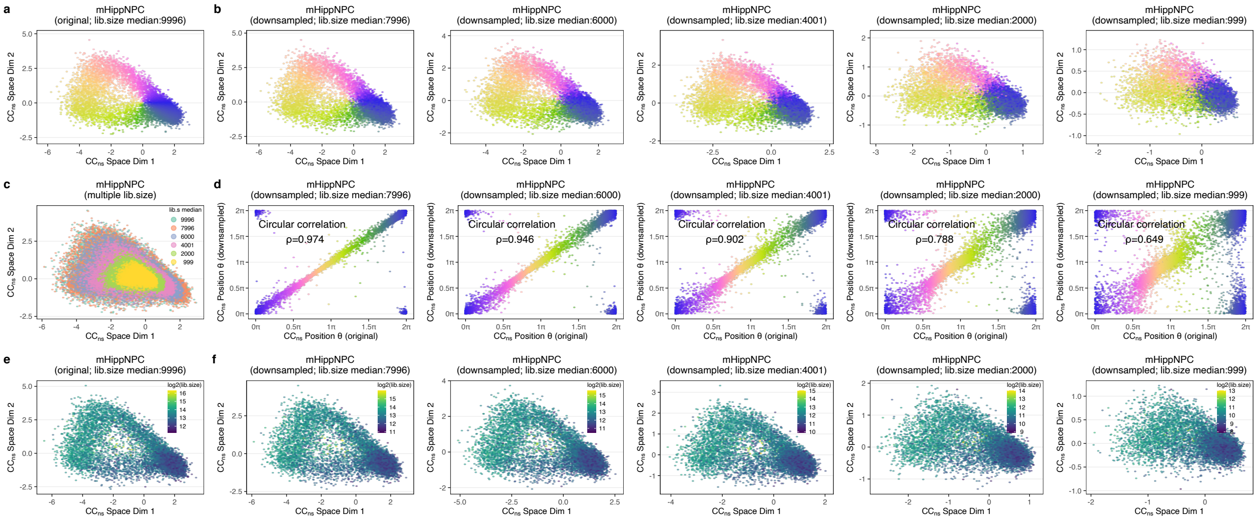

Fig. S32. Examples of projections on downsampled mHippNPC dataset. (a) The cell cycle embedding projection using the mNeurosphere reference on original mHippNPC data. Each point is a cell, colored by cell cycle position (polar angle in the embeddings). **(b)** Each sub-panel represents the same projection as in (a), but the mHippNPC is downsampled to the 80%, 60%, 40%, 20%, and 10% of its original library size (corresponding to median of library size is given in the panel title). Note that the ranges of both x-axis and y-axis are different across sub-panels. Cells are colored by the cell cycle position inferred on original data. **(c)** By overlaying (a) and all sub-panels of (b), it shows the shrinkage of projections with library size decreasing. **(d)** Comparisons of cell cycle positions θ estimated from the original mHippNPC data and from the downsampled mHippNPC data. Cells are colored by the cell cycle position inferred on original data (x-axis). **(e)** Similar to (a), but the points are colored by $\log_2$ transformed library size. **(f)** Similar to (b), but the points are colored by $\log_2$ transformed library size.

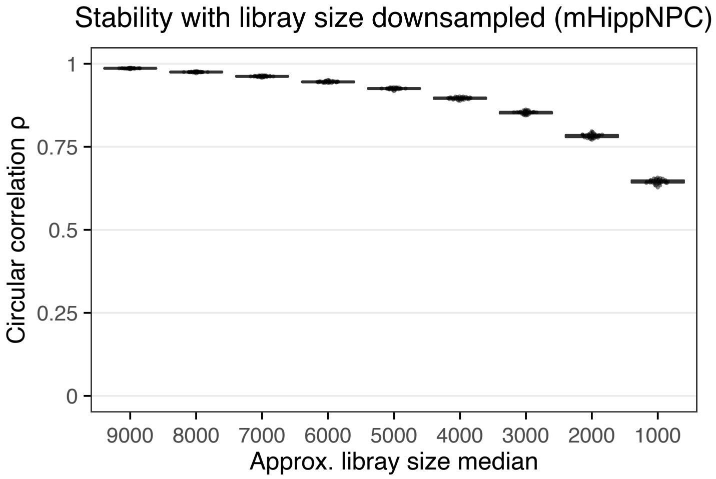

Fig. S33. Stability assessment with decreasing sequencing depths. This figure shows comprehensive assessments as complement to Fig. S32. We repeated the downsampling processes for mHippNPC for each target downsampling percentage. For each run, the circular correlation coefficient ρ was calculated between θ estimated on the original mHippNPC data and θ estimated on the downsampled data.

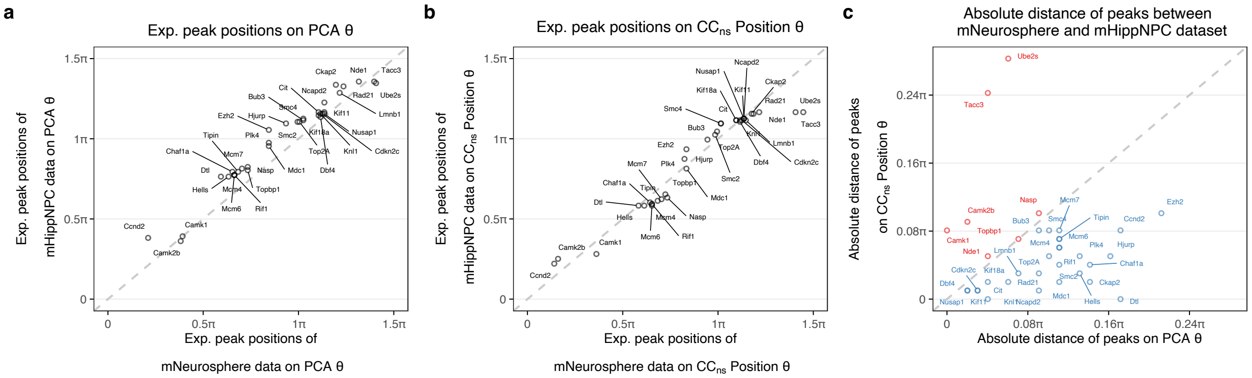

Fig. S34. Comparison of positions of peak expression for θ estimated on independent PCA and projection by mNeurosphere reference. (a) For each gene dipicted in Fig. S6, we estimate and compare when the peak expression is reached between 0 to 2π for mNeurosphere and mHippNPC data. The position θ is based on independent PCA on GO cell cycle genes of each data. **(b)** Similar to (a), but now we use position θ estimated using pre-learned mNeurosphere reference. **(c)** The majority of genes are better aligned on θ pre-learned mNeurosphere reference. x-axis represents the absolute distance of position of peak expression on θ estimated on independent PCA, while y-axis represents those estimated using pre-learned mNeurosphere reference. Genes with a larger absolute distance on θ estimated on independent PCA compared to θ estimated using pre-learned mNeurosphere reference are colored as blue, and genes are colored by red if showing the opposite direction.

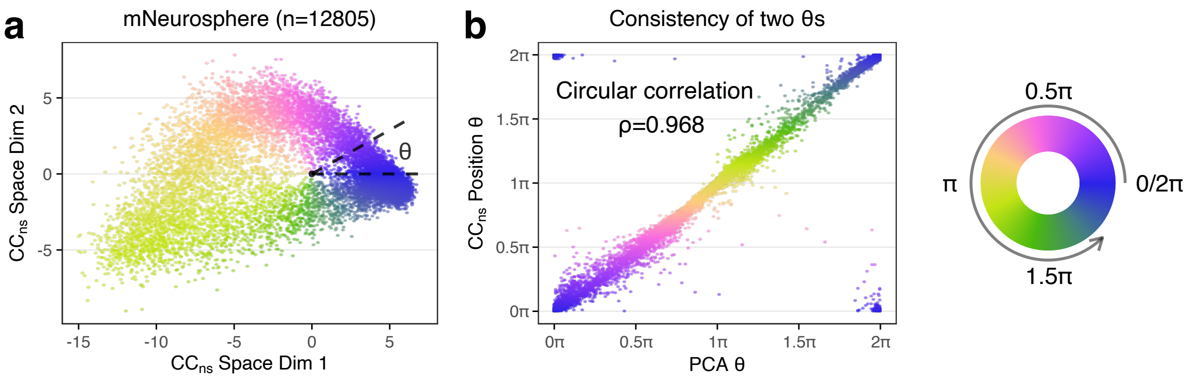

Fig. S35. Self-projection to test method sensitivity on a positive control. (a) The cell cycle embedding of mNeurosphere data using the reference learned from itself. Note the projections are different from direct PCA, as the PCA is done on Seurat corrected expressions while the projection is calculated on non-corrected expressions. **(b)** Comparisons of cell cycle positions θ estimated from the direct PCA and from the projection. Cells are colored by cell cycle positions θ estimated from the projection.

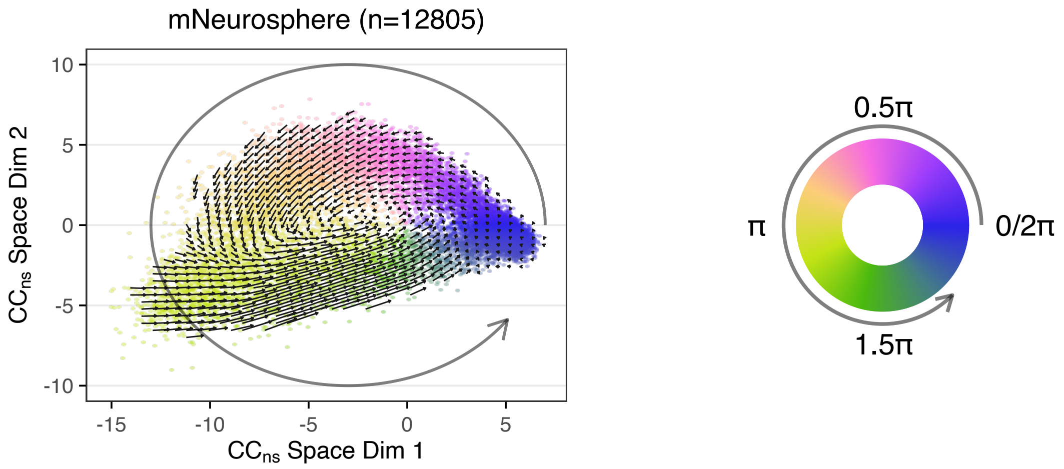

Fig. S36. RNA velocity projection into the cell cycle embedding. The arrows are projected RNA velocity estimations from the RNA velocity dynamical model in Bergen et al. (2020). Cells are colored by our cell cycle positions θ. The directions of RNA velocity projections are consistent with the directions of cell cycle positions θ.
