# Supplementary material for: Universal prediction of cell-cycle position using transfer learning
Source: Genome Biol. 2022 Jan 31;23:41. doi: 10.1186/s13059-021-02581-y (PMC8802487; doi:10.1186/s13059-021-02581-y)
Supplement: Supplementary file 2 — Additional file 2 Review history. [file 13059_2021_2581_MOESM2_ESM.docx]

Review History

**First round of review**

**Reviewer 1**

**Are you able to assess all statistics in the manuscript, including the appropriateness of statistical tests used?**

Yes, and I have assessed the statistics in my report.

**Comments to author:**

Zheng et al. propose a new computational method for inferring the cell cycle state of individual cells in single-cell RNA-seq data. This is a relatively crowded area of research where multiple methods have already been published, as the authors properly acknowledge. However, the application of current methods to noisy and heterogeneous datasets, as well as to datasets where cell cycle is strongly coupled to other cellular processes like cell differentiation, often leads to limited results. The method presented here is related to that of Schwabe et al. Mol. Sys. Bio. 16 (2020) in that it performs PCA of cell cycle genes to define a circular embedding where the cell cycle stage of each cell is manifest. However, in contrast with Schwabe et al., the embedding is defined using a high-quality and relatively homogeneous dataset that is then used as a reference for projecting (or "transferring") query datasets on. The manuscript includes applications to a great variety of datasets as well as detailed comparisons to most of the state of the art methods. The presentation is clear and rigorous, and the analyses presented make a convincing case that this new method is more robust than current methods in various applications to noisy and heterogeneous datasets. The accompanying software is well documented and I was able to successfully use it with my own data. In my opinion, this computational tool will be useful for the research community and the manuscript can be published in its current form, with no need for additional analyses or edits.

These are some typos I found:

- In page 6, second column, first and second paragraphs, I believe "(Figure 3b,c)" should read "(Figure 3b)", and "(Supplementary Figure S8a,b)" should read "(Figure 3c and Supplementary Figure S8a,b)" ?

- In page 11, second column, line 50, "compare" should read "compared"

In addition, these are some thoughts that might be useful for the authors (but that do not require any changes in the manuscript):

- An assumption made by the authors is that each gene has at most one peak over the time period (page 3, first column, line 61), so that the high-dimensional embedding of the cycle is linear and it is endured that the 2d PCA projection is circular. This assumption could be relaxed by applying the Mapper algorithm (Singh, Memoli, Carlsson "Topological Methods for the Analysis of High Dimensional Data Sets and 3D Object Recognition" (2007)) to the 2d PCA space. Consider for instance Fig. 1a of Rizvi et al. Nat. Biotechnol. 35 (2017), where a non-linear 3d embedding of a cycle is presented together with its 2d ICA/PCA projection, and its circular resolution using Mapper.

- A technical caveat of the cell cycle position is that it depends on the choice of origin of coordinates in the 2d PCA projection. This could be avoided by using some of the more rigorous circular coordinatization methods published in the mathematics literature (see for example, Perea "Sparse circular coordinates via principal Z-bundles" (2020)).

Nevertheless, it is expected these two aspects to be negligible in the vast majority of practical situations, and it is probably not worth implementing these extensions.

Pablo Camara

**Reviewer 2**

**Are you able to assess all statistics in the manuscript, including the appropriateness of statistical tests used?**

Yes, and I have assessed the statistics in my report.

**Comments to author:**

Review of Zheng et al

This paper provides a new approach (tricycle) for predicting the position of a cell in the cell cycle. The approach claims to work regardless of cell type, species and sequencing assay. There are several things to like about the paper. In particular, the tricycle method is very simple: i) compute the first two PCs on 500 annotated cell-cycle genes in a data set where cell cycle is the primary source of variation; ii) project any future observations to this 2-d embedding and compute the polar angle to predict its cell cycle position. Further, the empirical results are promising. At the same time I think the paper could be substantially improved by removing or reducing some of the less innovative parts, toning down some of the rhetoric, and focussing on the most convincing empirical results. My comments expand on these suggestions.

Main comments:

1. I found most of the material on PCA not to be especially novel or interesting. The use of PCA to determine cell cycle position has a long history (including many papers cited here), and existing mathematical results already go far beyond the analysis presented here. The behavior of PCA on cyclic phenomena is much more general than presented here, and does not rely on sinusoidal functions or "two distinct peaks" etc. Rather it stems from the result that cyclic phenomona lead to circulant covariance matrices, and all circulant matrices have the same eigenvectors: the columns of the discrete Fourier transform matrix. The result is that, when the covariance patterns primarily reflect cyclic phenomoena, the first two PCs will form a circle/ellipse. See Novembre and Stephens (2008) and references therein for further background.

Figure 1 is useful for summarizing the method, but most of the other material could be condensed or removed and I think the paper would be improved because it would better focus on what is actually new and interesting, the tricycle method (currently not introduced until p6) and the empirical assessments of its performance.

2. The paper left me asking myself this: what is the strongest empirical support that cell cycle assignments work in practice? To me, Fig 5 panels c and g are the most convincing, because they are quantitative comparisons with an alternative technology (and one that is often considered the "gold standard" in this area). I also liked the quantitative comparisons with other methods, and it seems some of those might be worth including in the main text. In contrast, the results in Fig 4 are not quantitative, and overall not that compelling. The top row of panels are kind of useful in demonstrating you get something like a circle. but we don't actually know that this corresponds to cell cycle from this picture (unless I misunderstood, the colors are inferred, not known). And looking at the mPancreas results one might be tempted to use (-3,0) as the center of the circle, which would change computation of polar angle quite a bit.

Is there reason to think that sticking with (0,0) is better? If so, any idea why does the circle show this shift? (Similar issues arise, to a lesser extent, with HippNPC). The Top2A results are, on their own, too noisy to be convincing -- why not show R2 plots for all cell-cycle genes (which could be contrasted with non-cell-cycle genes, and also compared with other methods). And as far as I can see Fig 4c is, at best, only interesting once one is convinced that the cell cycle is being correctly inferred -- nothing here to say that the cell cycle inferences are accurate.

To be clear, I'm not saying the method does not generalize well across data sets; I'm saying that the evidence for this needs to be more clearly presented.

3. A less fundamental issue: I don't really think describing this as an example of "transfer learning" is helpful. Indeed it is not even clear to me it is accurate. For example, in the cited Pan et al 2008, they describe the transfer learning problem as follows: "In a transfer learning setting, some labeled data Dsrc are available in a source domain, while only unlabeled data Dtar are available in the target domain." That does not apply here - everything is based on unlabelled data.

More generally, giving the approach a name like "transfer learning" seems to suggest that there is something going on to actually make this transfer from one dataset to another, or some deeper theoretical reason to think it should work -- but I don't believe either of these is true. You are just hoping that the PC weights learned in one (carefully chosen) data set will also work to capture cell cycle on other data sets. It isn't obvious in advance that this rather simple approach would work well, and the major contribution of the paper is to assess this empirically.

4. The abstract is hyperbolic. "ubiquitous applicability of transfer learning"; "can predict any cell's position in the cell cycle", "universally accurate", "eminently pertinent"...

5. As far as is practical please provide code and instructions to reproduce the main results

Minor:

- p2 you introduce the term "cell cycle pseudotime" only to explain later that it is not really a time at all. Why not just go straight into "cell cycle position" or "cell cycle phase"? (Also, the term "wall time" may not be familiar to all readers?)

-p5 l57: Figure 2d-> 2f?

- p8, line 32 right column: is "superficial" the right word here?

- Some of the loess fits (eg Fig 2 d-f; Fig 4 panel b, especially mHippNPC) don't look visually very good. Is this just an artifact of having 0s, whose density is impossible to see due to overplotting, or is loess over-smoothing? Might trend filtering, as used in Hsiao et al, work better?

Refs:

J Novembre and M Stephens. Interpreting principal component analyses of spatial population genetic variation. Nat Genet 40(5):646-649, May 2008

**Reviewer 3**

**Are you able to assess all statistics in the manuscript, including the appropriateness of statistical tests used?**

Yes, and I have assessed the statistics in my report.

**Comments to author:**

In this manuscript by Zheng and colleagues, the authors describe Tricycle, a method implemented as a R/Bioconductor package that infers cell cycle position from single-cell transcriptomics (scRNA-seq) data. The work tries to address the concern that while cell cycle represents cells in continuum, existing methods (reCAT, scLVM, Oscope, Cyclone, Seurat-based-cell cycle classification etc., with exception of Peco and Revelio) categorize cells into discreet cell cycle phases. Tricycle works by defining a reference 2-dimensional PCA embedding from a scRNA-seq dataset (i.e., their mNeurosphere dataset), inferring cell cycle pseudotime as an ellipsoid or the polar angle around the origin (ranging from 0-2) using cell cycle genes alone. Projecting the PC's of new datasets onto the reference embedding using orthogonal operator (transfer learning), Tricycle aims to infer the cell cycle position of single-cells (driven predominantly by G2M markers). The authors state that their approach is robust to technical/biological (batch) challenges, universally applicable to different scRNA-seq platforms, species (Mouse and Human) and scalable to atlas-scale datasets.

While the idea builds on concepts and findings from existing papers (Revelio, peco, scLVM), and is indeed interesting and the method could potentially be useful to the community. There are a few major and minor concerns stated below, which the authors need to address.

- The mNeurosphere and mHippNPC datasets were generated using 10x chromium for this study. However, no ground truth was empirically measured (by an alternative approach) and hence the tricycle inferred cell cycle position of single-cells is presumed to be correct. The ground truth measurement from alternative approaches (FUCCI, synchronization etc.,), although have caveats, but still provide a reference to compare.

- Consequently (in Figure S4), it is unclear which cell cycle staging should be considered correct. There is some congruence between cyclone and Seurat, but large discrepancies and misclassifications between Tricycle and other methods.

- The lack of consensus between Tricycle and other methods (inferred or ground truth) is very concerning (even for discrete stages core-G1, core-S and core-G2). It implies that other methods are not robust in capturing the continuum, but the same argument can applied to this work. The authors can address these issues by using datasets with experimental ground-truth as reference label and project other studies and cross-compare inferred cell stage labels.

- Capturing an ellipsoid structure from PCA (PC1-2 on cell cycle genes) in both reference and target datasets seems to be critical for inferring cell cycle position. While the authors show this to be case in down-sampled mNeurosphere and mHippNPC datasets (and simulated datasets), it is unclear how well this works for sparser target datasets with multiple cell types.

- Particularly for the mPancreas dataset, it is not clear why Tricycle fails even when using cell cycle genes. The authors argue the tight coupling and functional roles of cell cycle genes in differentiation process. However, this brings an additional concern on the broader applicability of tricycle to most studies which include multiple cell types over differentiation/development or processes.

- Tricycle orders the single-cells over cell cycle from 0-2 (driven predominantly by G2/M markers peaking at 1). However, the phase positions vary significantly between datasets. For e.g., Top2a peaks at 1 in Fig. 4, but for HeLa/mHSCs cells (Fig. S9/S10), the peak shifts to after 1. This indicates either variability in projected positioning of cells over cell cycle ().

- In Fig. 5, It is not clear why for the Mahdessian et al., the cell cycle pseduotime ranges from 0.8-to-0.8 (FUCCI pseudotime 0), while this isn't the case for other datasets (incl. Oscope and Revelio). Similarly (in Fig. S12), its unclear why Geminin levels don't correlate with both FUCCI pseudotime and CC position (should be very strong correlation with Geminin and less with Cdt1).

- The authors should demonstrate how Tricycle's works when using available scRNA-seq datasets with known ground truth as reference, and projecting to their in-house datasets and other public datasets (with ground-truth) across different cell types. This comparison would also indicate where the tricycle-inferred position on cell cycle pseudotime (0-2) is consistent across different cell types (i.e. S/G2/M ranges from 1.5 - 0.25), or whether varies for different cell types (different cycling speeds and/or time across each phase).

- (Minor) The size and resolutions of several main and supplementary figures was very poor (even for visualization on screen). For several figures showing multiple colors over cell cycle continuum, the stages were barely discernable, and often the legends, highlighted names were barely readable. The authors could either provide original resolution figures (separately) instead embedding within text or make figures larger in panels.

- (Minor) Fig. S30, typo in title (mNeurosphere).

**Authors Response**

**Point-by-point responses to the reviewers’ comments:**

Reviewer 1:

Zheng et al. propose a new computational method for inferring the cell cycle state of individual cells in single-cell RNA-seq data. This is a relatively crowded area of research where multiple methods have already been published, as the authors properly acknowledge. However, the application of current methods to noisy and heterogeneous datasets, as well as to datasets where cell cycle is strongly coupled to other cellular processes like cell differentiation, often leads to limited results. The method presented here is related to that of Schwabe et al. Mol. Sys. Bio. 16 (2020) in that it performs PCA of cell cycle genes to define a circular embedding where the cell cycle stage of each cell is manifest. However, in contrast with Schwabe et al., the embedding is defined using a high-quality and relatively homogeneous dataset that is then used as a reference for projecting (or "transferring") query datasets on. The manuscript includes applications to a great variety of datasets as well as detailed comparisons to most of the state of the art methods. The presentation is clear and rigorous, and the analyses presented make a convincing case that this new method is more robust than current methods in various applications to noisy and heterogeneous datasets. The accompanying software is well documented and I was able to successfully use it with my own data. In my opinion, this computational tool will be useful for the research community and the manuscript can be published in its current form, with no need for additional analyses or edits.

*Response: We would like to thank the reviewer for taking time to evaluate our manuscript and tool. The evaluation of tricycle on their data supports both their and our conclusion that tricycle will be useful for the research community. Further, we appreciate the re¬viewers conclusion that tricycle is more robust than current methods in various appli¬cations to noisy and heterogeneous datasets especially since, as the reviewer states, we have demonstrated “applications to a great variety of datasets as well as detailed com¬parisons to most of the state of the art methods”.*

These are some typos I found:

- In page 6, second column, first and second paragraphs, I believe "(Figure 3b,c)" should read "(Figure 3b)", and "(Supplementary Figure S8a,b)" should read "(Figure 3c and Supplementary Figure S8a,b)" ?

- In page 11, second column, line 50, "compare" should read "compared"

*Response: We have fixed the figure references as suggested. On page 6, these two sen¬tences now read*

*“ When we perform principal component analysis using only the variable cell cycle genes, the resulting PCA plot still reflects the differentiation trajectory and does not resemble the ellipsoid pattern observed in the previous section (Figure 3b)/'*

*and*

*“ However, when we perform principal component analysis only on a subset of cells from a sin¬gle, proliferating progenitor cell type, the ellipsoid pattern returns (Figure 3c and Supplementary Figure S8a,b)/'*

In addition, these are some thoughts that might be useful for the authors (but that do not require any changes in the manuscript):

- An assumption made by the authors is that each gene has at most one peak over the time period (page 3, first column, line 61), so that the high-dimensional embedding of the cycle is linear and it is endured that the 2d PCA projection is circular. This assumption could be relaxed by applying the Mapper algorithm (Singh, Memoli, Carlsson "Topological Methods for the Analysis of High Dimensional Data Sets and 3D Object Recognition" (2007)) to the 2d PCA space. Consider for instance Fig. 1a of Rizvi et al. Nat. Biotechnol. 35 (2017), where a non-linear 3d embedding of a cycle is presented together with its 2d ICA/PCA projection, and its circular resolution using Mapper.

*Response:*

*We thank the reviewer for the suggestion. The Mapper algorithm is indeed a very general solution when the circle is twisted in the high dimensional space. In simulations, we can generate such behaviour when we simulate under a model where each gene has a variable number of peaks. However, it is pretty clear that this behaviour is not relevant to cell cycle genes (although it might be relevant to other periodic/cycling behaviour in genomics). In fact, one of the main empirical insights from our work is the observation that projecting new data into a fixed reference embedding always (or at least for every single dataset we have ever considered) yields an ellipsoid. For this reason, we have decided to keep our current approach; although, we now correctly cite scTDA. We have thought about working on extending our approach to more general cycling processes, such as circadian rhythm, and for such work it would be quite interesting to apply Mapper / scTDA.*

- A technical caveat of the cell cycle position is that it depends on the choice of origin of coordinates in the 2d PCA projection. This could be avoided by using some of the more rigorous circular coordinatization methods published in the mathematics literature (see for example, Perea "Sparse circular coordinates via principal Z-bundles" (2020)).

*Response: As noted by the reviewer, we fix the choice of origin in our approach and one could certainly imagine improving on this state. We have experimented with moving the origin around and observe this has surprisingly little impact on the inferred cell cycle position (such as the example in Figure 1 for comment 2 of reviewer 2 below).*

*Having said that, a more principled approach to this would of course be ideal. We have read the Perea paper and are concerned about the sensitivity of the method to the con¬struction of the landmark set used for the Rips Filtration. We have demonstrated that the specific composition of cell cycle genes varies slightly, but nontrivially across datasets. Thus, the effect of enforcing a specific landmark set on the downstream distances is of potential concern. Perhaps, more importantly, the Perea paper does not include an imple¬mentation of the approach, and we have therefore been unable to experiment with this numerically.*

Nevertheless, it is expected these two aspects to be negligible in the vast majority of practical situations, and it is probably not worth implementing these extensions.

Reviewer 2:

This paper provides a new approach (tricycle) for predicting the position of a cell in the cell cycle. The approach claims to work regardless of cell type, species and sequencing assay. There are several things to like about the paper. In particular, the tricycle method is very simple: i) compute the first two PCs on 500 annotated cell-cycle genes in a data set where cell cycle is the primary source of variation; ii) project any future observations to this 2-d embedding and compute the polar angle to predict its cell cycle position. Further, the empirical results are promising. At the same time I think the paper could be substantially improved by removing or reducing some of the less innovative parts, toning down some of the rhetoric, and focussing on the most convincing empirical results. My comments expand on these suggestions.

*Response: We thank the reviewer for taking time to read and assess our manuscript, and for the constructive feedback.*

Main comments:

1. I found most of the material on PCA not to be especially novel or interesting. The use of PCA to determine cell cycle position has a long history (including many papers cited here), and existing mathematical results already go far beyond the analysis presented here. The behavior of PCA on cyclic phenomena is much more general than presented here, and does not rely on sinusoidal functions or "two distinct peaks" etc. Rather it stems from the result that cyclic phenomona lead to circulant covariance matrices, and all circulant matrices have the same eigenvectors: the columns of the discrete Fourier transform matrix. The result is that, when the covariance patterns primarily reflect cyclic phenomoena, the first two PCs will form a circle/ellipse. See Novembre and Stephens (2008) and references therein for further background.

Figure 1 is useful for summarizing the method, but most of the other material could be condensed or removed and I think the paper would be improved because it would better focus on what is actually new and interesting, the tricycle method (currently not introduced until p6) and the empirical assessments of its performance.

*Response: We thank the reviewer for the Novembre and Stephens (2008) reference (and more importantly, for the additionally references listed there, particularly Gray (2005)), which we have examined and which have expanded our understanding of the phenomenon.*

*Upon re-reading our exposition we very much agree with the reviewer that we gave the wrong impression that a single peak per gene was required to get a circle in the PCA plot – this is clearly wrong and we have revised our text to better reflect this point. However, we think it still makes sense to mention that the single peak per gene feature of the cell cycle makes the ”circle/time ordering” problem particularly easy. Two examples illustrate this. First, assume all genes have exactly two peaks. In that case, you get a circle in the PCA plot but you’re unable to infer the true time ordering; it is only possible to infer location within one period (granted, this is a pathological case). Second, we have done some simulations with 1-2 peak locations per gene and negative binomial noise (Figure 1 for comment 1 of reviewer 2) where the you can recover time ordering by looking at PC2 and PC3, but clearly not by PC1 and PC2.*

*We have changed the text in multiple places, most importantly in the Results section titled “Principal component analysis and periodic functions”.*

*We have kept our Methods section on the mathematics. We believe a constructive proof is useful to the community; the existing mathematical theory takes a while to appreciate.*

*Figure 1 for comment 1 of reviewer 2. (a) Expression vs. time for 2 genes with two periods (2π and π). In total, 50 genes with period 2π and other 50 genes with period π are simulated for 5,000 time points (cells). The noise follows a negative binomial distribution. (b) Principal component 1 and 2 of the simulated data from (a). (c) Similar to (b), but showing principal component 1 and 3. (d) Similar to (b), but showing principal component 1 and 4. (e) Similar to (b), but showing principal component 2 and 3. (f) Similar to (b), but showing principal component 2 and 4. (g) Inferred time point by the angle formed by PC1 and PC2. vs. true time point. (h) Inferred time point by the angle formed by PC2 and PC3. vs. true time point.*

2. The paper left me asking myself this: what is the strongest empirical support that cell cycle assignments work in practice? To me, Fig 5 panels c and g are the most convincing, because they are quantitative comparisons with an alternative technology (and one that is often considered the "gold standard" in this area). I also liked the quantitative comparisons with other methods, and it seems some of those might be worth including in the main text. In contrast, the results in Fig 4 are not quantitative, and overall not that compelling. The top row of panels are kind of useful in demonstrating you get something like a circle. but we don't actually know that this corresponds to cell cycle from this picture (unless I misunderstood, the colors are inferred, not known). And looking at the mPancreas results one might be tempted to use (-3,0) as the center of the circle, which would change computation of polar angle quite a bit.

Is there reason to think that sticking with (0,0) is better? If so, any idea why does the circle show this shift? (Similar issues arise, to a lesser extent, with HippNPC). The Top2A results are, on their own, too noisy to be convincing -- why not show R2 plots for all cell-cycle genes (which could be contrasted with non-cell-cycle genes, and also compared with other methods). And as far as I can see Fig 4c is, at best, only interesting once one is convinced that the cell cycle is being correctly inferred -- nothing here to say that the cell cycle inferences are accurate.

To be clear, I'm not saying the method does not generalize well across data sets; I'm saying that the evidence for this needs to be more clearly presented.

*Response: The reviewer is asking an important question: how do we weigh the evidence we present (as well as providing a number of interesting suggestions). This has helped to clarify our thinking. We categorize the evidence into 2 main groups.*

*·	”Internal controls”. Whenever a method supplies a continuous time prediction, you can investigate the expression dynamics (and optionally log totalUMI) across the inferred 0 ordering to see if they are as expected. We call this internal controls be¬cause it is a set of controls which are intrinsically available within each dataset; this is an unusual situation in genomics. In our opinion, these internal controls can be exploited to evaluate whether the inferred position reflects the known temporal bi¬ology of the cell cycle.*

*·	Gold standard datasets (”external controls”). Here, we particularly like the FUCCI platform because it is the only one with the potential for a continuous time prediction despite its drawbacks.*

*One critical limitation of the FUCCI datasets is that they represent easy situations: they only a single cell type and the cells are relatively deeply sequenced.*

*We have substantially revised the manuscript to include the term “internal controls” and have tried to refer to that throughout. We have also added substantial text to the Discus¬sion on this specific important point.*

*“ To address the limitation of gold-standard measurements on cell lines, we have made extensive use of internal controls. Specifically, we use inferred cell cycle position to assess whether key cell cycle genes (and log totalUMI if available) exhibit the expected expression dynamics across the learned 0 progression. These internal controls are available in any single-cell expression dataset, including complex tissue samples. These internal controls do not by themselves give a clear answer to how precise the predictions are, but they undoubtedly carry some information on whether the inferred cell cycle position is at all associated with cell cycle phases. By using these internal controls, we overcome the limitations of the available gold standard datasets – which we can think of as having “external controls” – and show that tricycle performs well on differentiation datasets and datasets with multiple cell types. Additionally, we are able to ascertain the generalizability of the method. The cell cycle genes we use for evaluation are also used to construct the reference embedding and for projection. However, at the projection stage, the weights are fixed without any dataset dependent optimization. This removes – in our opinion – any circular reasoning. We note that internal controls are useful to assess any continuous prediction of cell cycle position.”*

*The reviewer is noting that it is a weakness that we only use a single gene (Top2A) throughout our manuscript. We do that because it is our experience that if (a) the embed¬ding looks ellipsoid and (b) Top2A looks good, then we always get the same conclusion when considering additional genes. To overcome the limitation of plotting a single gene, the reviewer is suggesting to summarize them, for example, by R2 and perhaps by con¬trasting R2 between genes related to the cell cycle and not. We have tried this, and we do not think it works well. The issue is basically that R2 is not really comparable between genes and definitely not comparable between datasets. We observe that we frequently have cell cycle genes with low R2 (sometimes because they are not detected in the exper¬iment) and we also have some non-cell cycle genes (genes not in the GO cell cycle term) with very high R2 values, but upon inspection those genes have been reported by other papers as being associated with the cell cycle. In short, this suggestion does not (in our testing) really allow us to summarize the data in any sensible, consistent, and satisfying way. We do use R2 to compare across methods and this works because in that comparison we fix the dataset, we fix the gene, and we only change the predicted time variable. In this manner, we compare apples to apples (and we have now included more of these R2 comparisons between methods). We have also tried to come up with alternative ways of summarizing the gene expression dynamics and we have been unable to create something that works better than the Top2A plot.*

*The reviewer comments extensively on Figure 4. We have covered the Top2A plots above (which we believe to be the best supporting evidence in the figure). The top row, which shows that our projection method results in a ellipsoid shape, is an important foundation for the entire method and is used to contrast with Figure 3 (standard PCA of a dataset with multiple cell types). We now comment on this contrast explicitly in the description of Fig¬ure 4. The bottom row (colored UMAP) is not evidence per se, but rather an illustration of how cell cycle can be visualized on a plot many biologists believe reveals important structure in the data. The reviewer may disagree with this final point (“revealing impor¬tant structure”) but we note that we are not emphasizing this point in our manuscript, but rather attempting to illustrate the tricycle projection using a representation that is familiar to our target users.*

*Now, the choice of origin. We believe that this choice is, broadly speaking, not of sig¬nificant importance to the overall output of tricycle. At the reviewer’s suggestion, we now provide Figure 1 for comment 2 of reviewer where we have moved the origin and it is clear that the impact on cell cycle position is relatively minor (and the ordering is unaffected). Having said that, we do believe the origin is affected by a number of factors including (1) which of the 500 cell cycle genes are above detection in a given dataset (2) the proportion of G0 cells in a dataset. We observe (as pointed out by reviewer 3) that the precise location of the mode of Top2A differs slightly between datasets. This prevents us – at the current state of the method – from making very precise comparisons between dif¬ferent datasets (our caveats here are explained in the new paragraphs in the Discussion). All of this is now included in our greatly revised Discussion section.*

*“ There are two specific sources of variation we want to highlight. First is the variation in nonzero expression. Specifically, we observe that 300-500 genes – out of a total of 500 genes – have non-zero expression in a given dataset, and we show that differences in these genes can cause changes in the shape of the embedding (Supplementary Figures S28, S29, and S30). These differences in nonzero expression are also connected to the utility of using a larger set of genes to define the embedding space, as discussed above.*

*Second, the variation in the proportion of cells in G0 and G1, which is associated with the actual wall clock length of the cell cycle. Reflecting the biology of the system, this ratio also affects the placement of the origin of the projected data. However, this also potentially complicates across data set comparisons, as the only normalization currently performed in tricycle is the mean centering of each gene, which is susceptible to differences in this ratio. Lastly, we note that the peak expression location variations might be a consequence of different cell cycle dynamics in different systems. We hope the biological implications will be examined closely in the future. Methods to expand tricycle to allow cross data comparisons are currently an active area of research.”*

*Figure 1 for comment 2 of reviewer 2. Consistency of inferred cell cycle position using two different centers for mPancreas dataset.*

*Finally, we are appreciative that the reviewer likes our quantitative comparisons with other methods, and we have also learned about the problem of cell cycle estimation from doing these comparisons. Of paramount concern with this study is that we have a large number of interesting observations and we have had to choose which to focus on. Given that the methods against which we compare are widely variable in their useability, scal-ability, generalizability, and output (e.g. discrete predictions, soft-assignments, or continuous predictions), a comprehensive comparison requires a substantial amount of text. Given the amount of material we already include, we still believe it is sensible to delegate this to the supplement and just keep the highlights.*

3. A less fundamental issue: I don't really think describing this as an example of "transfer learning" is helpful. Indeed it is not even clear to me it is accurate. For example, in the cited Pan et al 2008, they describe the transfer learning problem as follows: "In a transfer learning setting, some labeled data Dsrc are available in a source domain, while only unlabeled data Dtar are available in the target domain." That does not apply here - everything is based on unlabelled data.

More generally, giving the approach a name like "transfer learning" seems to suggest that there is something going on to actually make this transfer from one dataset to another, or some deeper theoretical reason to think it should work -- but I don't believe either of these is true. You are just hoping that the PC weights learned in one (carefully chosen) data set will also work to capture cell cycle on other data sets. It isn't obvious in advance that this rather simple approach would work well, and the major contribution of the paper is to assess this empirically.

*Response: What is actually meant by transfer learning seems to be poorly specified. We are citing Pan, Kwok, and Yang (2008) because it is credited in the machine learning liter¬ature for the claim that it is a smart idea to reduce data to a latent space and then combine them. We have now included a citation to a review (Pan and Yang, 2009) which goes into more detail. However, as the reviewer will surely note, definition 1 in that review is so broad as to be almost meaningless!*

*However, some of us have previously defined transfer learning specifically to be about using embeddings and projections (as we do here) to compare datasets, see Stein-O’Brien et al. (2019), and we use the term transfer learning in this sense, to at least be consistent with this and other prior work in the single cell analysis space. We agree that there is no real theory that justifies why this works, and we do see the empirical validation of tricycle to be a major step forward towards eventually understanding the basis of this approach.*

4. The abstract is hyperbolic. "ubiquitous applicability of transfer learning"; "can predict any cell's position in the cell cycle", "universally accurate", "eminently pertinent"...

*Response: We agree with the reviewer that we were too hyperbolic in our abstract. We have decided to keep the word ”Universal” in the title because we do believe that this approach works across any dataset (which is impossible to prove of course). We have otherwise modified the abstract to be more constrained.*

5. As far as is practical please provide code and instructions to reproduce the main results

*Response: We note that our method is fully documented as a Bioconductor package. In addition, we have now made the scripts used to produce all figures in the manuscript available at https://github.com/hansenlab/tricycle_paper_figs. Our pro¬cessed data is available at https://doi.org/10.5281/zenodo.5519841. These links have been added to our manuscript as well.*

Minor:

- p2 you introduce the term "cell cycle pseudotime" only to explain later that it is not really a time at all. Why not just go straight into "cell cycle position" or "cell cycle phase"? (Also, the term "wall time" may not be familiar to all readers?)

*Response: Unfortunately pseudotime has been used by other authors and is strongly related to the popular trajectory analysis methods which do not result in a circular time. We felt we needed a precise different word. We start off with pseudotime because of its usage in the literature. We are now explaining wall time in the text, with*

*“ and is unrelated to wall time (time measured by a clock in SI units)”*

-p5 l57: Figure 2d-> 2f?

*Response: The typo has been fixed now. It now reads*

*“ Figure 2f shows the log 2 transformed total UMI numbers against θ, with a periodic loess smoother for each dataset.”.*

- p8, line 32 right column: is "superficial" the right word here?

*Response: We have replaced ”superficial” with ”apparent”.*

- Some of the loess fits (eg Fig 2 d-f; Fig 4 panel b, especially mHippNPC) don't look visually very good. Is this just an artifact of having 0s, whose density is impossible to see due to overplotting, or is loess over-smoothing? Might trend filtering, as used in Hsiao et al, work better?

*Response: We thank the reviewer for the visualization suggestion. We have tried us¬ing the trendfilter smoother as used in Hsiao et al. (2020), shown in the Figure of using trendfilter below. Comparing the fitted lines of the two smothers, we did not find a clear improvement when using trendfilter. Trendfilter has a more rapid decline following π (which we think looks better), but is pretty unsmooth just before the peak (around 0.75π) (which we think is bad). We think this is the artifact of having 0s, which are unavoid¬able in most single-cell RNA-seq data. Considering trendfilter takes much more time to evaluate than loess, we chose to stick with loess.*

*Evaluation of fitting models (loess v trendfilter). (a-c) These panels are panel d-f of Figure 2 in the manuscript. (a,b) The expression dynamics of (a) Top2A and (b) Smc4 using the inferred cell cycle position, with a periodic loess line (Methods). (c) The dynamics of total UMI using the inferred cell cycle position, with a periodic loess line, illustrating the high agreement of the dynamics between datasets. (d-f) Similar to (a-c), instead of using loess smoother, we are now using trendfilter smoother as used in Hsiao et al. (2020).*

Refs:

J Novembre and M Stephens. Interpreting principal component analyses of spatial population genetic variation. Nat Genet 40(5):646-649, May 2008

Reviewer 3:

In this manuscript by Zheng and colleagues, the authors describe Tricycle, a method implemented as a R/Bioconductor package that infers cell cycle position from single-cell transcriptomics (scRNA-seq) data. The work tries to address the concern that while cell cycle represents cells in continuum, existing methods (reCAT, scLVM, Oscope, Cyclone, Seurat-based-cell cycle classification etc., with exception of Peco and Revelio) categorize cells into discreet cell cycle phases. Tricycle works by defining a reference 2-dimensional PCA embedding from a scRNA-seq dataset (i.e., their mNeurosphere dataset), inferring cell cycle pseudotime as an ellipsoid or the polar angle around the origin (ranging from 0-2) using cell cycle genes alone. Projecting the PC's of new datasets onto the reference embedding using orthogonal operator (transfer learning), Tricycle aims to infer the cell cycle position of single-cells (driven predominantly by G2M markers). The authors state that their approach is robust to technical/biological (batch) challenges, universally applicable to different scRNA-seq platforms, species (Mouse and Human) and scalable to atlas-scale datasets.

While the idea builds on concepts and findings from existing papers (Revelio, peco, scLVM), and is indeed interesting and the method could potentially be useful to the community. There are a few major and minor concerns stated below, which the authors need to address.

*Response: We thank the reviewer for pointing out our significant contribution to this area and their extensive feedback which has helped to improve the manuscript. We have made numerous revisions (detailed below) in order to improve the overall clarity of the manuscript and have completely revised the discussion based on this feedback.*

- The mNeurosphere and mHippNPC datasets were generated using 10x chromium for this study. However, no ground truth was empirically measured (by an alternative approach) and hence the tricycle inferred cell cycle position of single-cells is presumed to be correct. The ground truth measurement from alternative approaches (FUCCI, synchronization etc.,), although have caveats, but still provide a reference to compare.

*Response: To ensure a comprehensive response to this concern, we have addressed the question of gold standard measurements on both the dataset we use to construct the ref¬erence embedding (mNeurosphere) and for data measured using the 10x chromium plat¬form.*

*Re: gold standard measurements on the dataset used to construct the reference embed¬ding. To provide the most rigorous assessment of tricycle, we deliberately held out the gold standard datasets for validation rather than construction. We are much more inter¬ested in checking how tricycle works at predicting cell cycle position, and to do so we need to use a dataset with gold standard measurements, estimate cell cycle position and compare the estimate to the gold standard measurements. We argue –through the use of internal controls (a new term we use in our manuscript which describes the assessment of expression dynamics) that our inferred cell cycle progression on the reference dataset is reasonable. Specifically, we show the expression dynamics of the most important cell cycle genes and the number of total UMIs (proxy for cell size and DNA content) fit the inferred cell cycle position. In addition, we have shown that tricycle inferred cell cycle position agrees well with other methods on this dataset. This evidence does not prove the accuracy of our cell cycle position estimates, but it does strongly suggest that there is a true signal.*

*Re: gold standard measurements on 10x chromium data. The best gold-standard mea¬surements are based on the FUCCI platform and this requires imaging of individual cells and the ability to associate this image with the expression profile. This is technically very difficult to combine with a droplet based assay like 10x chromium. It is possible to synchronize cells or to FACS sort cells into very broad cell populations and then use the 10x platform, but this type of gold standard data, in which cells are collectively pre-aggregated by FACS gating is much less conclusive compared to imaging-based FUCCI analysis. We believe that our use of internal controls (total UMI as a function of cell cy¬cle position and expression dynamics of key cell cycle genes) strongly support that our method works well on 10x chromium data.*

- Consequently (in Figure S4), it is unclear which cell cycle staging should be considered correct. There is some congruence between cyclone and Seurat, but large discrepancies and misclassifications between Tricycle and other methods.

*Response: Importantly, Figure S4 does not contain any predictions from our method. In our original submission, we consistently colored embedding plots like Figure S4 by the 5-stage cell cycle prediction using the SchwabeCC estimator (from the Revelio paper), and we (confusingly) labelled this coloring ”CC stage”. This was confusing, because it could be interpreted to mean stage predictions obtained using tricycle, which are explicitly not determined using our method. To rectify this issue, we have taken the following steps*

*·	We have changed the coloring label from ”CC stage” to ”SchwabeCC” throughout the manuscript and have also emphasized this in each figure caption.*

*·	We have removed the SchwabeCC coloring from many figures where it was un¬necessary. In the original submission we had this coloring because we felt it help put our predictions in context by implicitly comparing to a different method, but in practice it is clearly confusing that we introduce another predictor every time we make this kind of plot.*

*This has led to a change in many panels of our figures including Figure 4a-b, Figure 5e-f, Supplementary Figure S9a-b, Supplementary Figure S10, Supplementary Figure S12, Supplementary Figure S13, Supplementary Figure S15, Supplementary Figure S17, Sup¬plementary Figure S28, Supplementary Figure S29, Supplementary Figure S31, and Sup¬plementary Figure S34. We thank the reviewer for pointing out this source of confusion and hope that addressing this point will clarify many of the reviewer’s concerns.*

*As an illustration of our high agreement between Seurat and Tricycle we have made a figure for the reviewer which compares Seurat stage predictions with tricycle time on a UMAP plot of our reference dataset (“Figure for comment 2 of reviewer 3”).*

*Figure for comment 2 of reviewer 3. Scatter plots show the UMAPs of Seurat3 mergerd Neurosphere data colored by (a) inferred cell cycle stage by Seurat, (b) cell cycle position inferred by tricycle.*

- The lack of consensus between Tricycle and other methods (inferred or ground truth) is very concerning (even for discrete stages core-G1, core-S and core-G2). It implies that other methods are not robust in capturing the continuum, but the same argument can applied to this work. The authors can address these issues by using datasets with experimental ground-truth as reference label and project other studies and cross-compare inferred cell stage labels.

*Response: We believe that the confusing plot coloring from the previous concern (concern 2) is also the culprit here. Specifically, our use of “CC stage” as a label when we really mean “Schwabe CC stage”. Therefore, the comment is really about a lack of consensus between Schwabe CC and other methods.*

- Capturing an ellipsoid structure from PCA (PC1-2 on cell cycle genes) in both reference and target datasets seems to be critical for inferring cell cycle position. While the authors show this to be case in down-sampled mNeurosphere and mHippNPC datasets (and simulated datasets), it is unclear how well this works for sparser target datasets with multiple cell types.

*Response: The reviewer highlights the importance of capturing an ellipsoid structure. As the reviewer points out, doing PCA on the target dataset does not ensure such a structure (which is true for the mPancreas data for example, see our Figure 3). One of our main contributions is the realization that we do obtain such a structure if we project the target data into the reference embedding. We observe this empirically on all datasets we consider. For this to be true, we believe it is necessary that the reference data is ellipsoidal but we have verified that for the reference data we use throughout (and which we recommend to be used in general): the mNeurosphere data.*

*To address the issue of sparsity we have added a sparse dataset (generated using com¬binatorial indexing) to Supplemental Figure S9 and have described this dataset in the section “Transfer learning through projection”. We do indeed get an ellipsoid structure although the ellipsoid is very tight (as we would expect based on our simulation analysis in the section “stability of the cell cycle position assignments”). When we evaluate the in¬ferred expression dynamics (eg. Top2A), they show the expected behavior which strongly suggests we are inferring a reasonable cell cycle time ordering. In addition to this mate¬rial, we also have an extended application of tricycle to a human fetal tissue atlas, which encompass 15 sparse datasets with multiple cell types (relatively lightly sequenced with a median of 429 - 892 total UMIs for 4 single-cell profiled tissues and 354 - 795 for 11 single-nuclei profiled tissues), in addition to the down-sample simulations. We have dedicated a subsection to describe the application results on page 10-11:*

*“ Within tissues, lymphoid cells are often the cell type with the highest proliferation index (Supple-mentary Figures S25 and S26); often with a greater number of actively proliferating cells than not. Within thefetal liver and spleen – both sites of early embryonic erythropoiesis during human devel¬opment (Cumano and Godin, 2007) – erythroblasts represent the cell type with the highest fraction of proliferating cells. Across developmental time, most tissues maintain relatively monotonic pro¬liferation indices, however several (liver, placenta, intestine) exhibit dynamic changes across the sampled timepoints. This application illustrates the utility of tricycle to atlas-level data.”*

- Particularly for the mPancreas dataset, it is not clear why Tricycle fails even when using cell cycle genes. The authors argue the tight coupling and functional roles of cell cycle genes in differentiation process. However, this brings an additional concern on the broader applicability of tricycle to most studies which include multiple cell types over differentiation/development or processes.

*Response: We believe this concern is a result of the same confusing plot coloring we have already addressed as tricycle does not fail on the mPancreas dataset. Indeed, for the mPancreas dataset, running PCA on cell cycle genes fails (Figure 3b), but tricycle works well and successfully recovers the cell cycle progression. The next couple of paragraphs describes this in more detail.*

*In our manuscript, we first introduce the mPancreas dataset in the sub-section ”When principal component analysis fails to reflect cell cycle position”. In Figure 3b, we explicitly show that the first two PCs did not form an ellipsoid shape and we interpret this to be because of the tight coupling and functional roles of cell cycle genes. The PCA failure corresponds to the reviewer’s comment of ”the tight coupling and functional roles of cell cycle genes in differentiation process.”*

*However, when we later show tricycle’s results in sub-section ”Transfer learning through projection”, Figure 4 second column shows that tricycle works well on the mPancreas dataset. We put this contrast example in our manuscript to highlight tricycle’s superiority over PCA, and that tricycle works well on datasets which include multiple cell types over differentiation/development or processes.*

*Altogether this is an important point (dealing with datasets with multiple cell types) and we apologize that our figure coloring / labelling has obscured this in our previous ver¬sion. And we have now emphasized that tricycle works well on datasets which include multiple cell types over differentiation/development in the last paragraph of sub-section ”Transfer learning through projection”:*

*“ We draw attention to two specific datasets in these figures: the mPancreas and mRetina datasets (Figure 4) and the HCA (human cell atlas) Pancreas data (Supplementary Figure S9). These three datasets contain many different cell types and strong drivers of gene expression in addition to the cell cycle, such as differentiation. We have previously seen how principal component analysis fails to be ellipsoid on the mPancreas dataset (Figure 3) and this example shows how tricycle – by projecting the data into a fixed reference embedding – overcomes the limitations of principal component analysis.”*

- Tricycle orders the single-cells over cell cycle from 0-2 (driven predominantly by G2/M markers peaking at 1). However, the phase positions vary significantly between datasets. For e.g., Top2a peaks at 1 in Fig. 4, but for HeLa/mHSCs cells (Fig. S9/S10), the peak shifts to after 1. This indicates either variability in projected positioning of cells over cell cycle ().

*Response: We do realize there are some variability in the exact peak location across datasets, although we still maintain that they are largely similar (it’s not the case that Top2A sudden peaks at 2ir for example). We hypothesize that this variability is caused by several factors. They include differences in platforms and also differences in which genes are detected in each dataset, and therefore meaningfully contribute to the projec¬tion. These differences slightly alter the projection of the new data into the reference em¬bedding and we get some variation in peak position as a result. We discuss the specific effect of including a subset of our 500 projection genes in our manuscript (Supplemental Figures S28, S29 and S30) which mimics the situation where only some of the genes are above detection level in a new dataset. We conclude that there is a good agreement be¬tween the inferred cell cycle positions (and most importantly: their ordering) although there can be some local changes (see for example the scatterplot in S28b). Finally, it may also be the case that there is biological variation in the relative position of peak gene ex¬pression across datasets, which is an area of future exploration for us.*

*This is an important discussion point and we thank the reviewer for raising it. We now write in the Discussion*

*“ There are two specific sources of variation we want to highlight. First is the variation in nonzero expression. Specifically, we observe that 300-500 genes – out of a total of 500 genes – have non-zero expression in a given dataset, and we show that differences in these genes can cause changes in the shape of the embedding (Supplementary Figures S28, S29, and S30). These differences in nonzero expression are also connected to the utility of using a larger set of genes to define the embedding space, as discussed above.*

*Second, the variation in the proportion of cells in G0 and G1, which is associated with the actual wall clock length of the cell cycle. Reflecting the biology of the system, this ratio also affects the placement of the origin of the projected data. However, this also potentially complicates across data set comparisons, as the only normalization currently performed in tricycle is the mean centering of each gene, which is susceptible to differences in this ratio. Lastly, we note that the peak expression location variations might be a consequence of different cell cycle dynamics in different systems. We hope the biological implications will be examined closely in the future. Methods to expand tricycle to allow cross data comparisons are currently an active area of research.”*

- In Fig. 5, It is not clear why for the Mahdessian et al., the cell cycle pseduotime ranges from 0.8-to-0.8 (FUCCI pseudotime 0), while this isn't the case for other datasets (incl. Oscope and Revelio). Similarly (in Fig. S12), its unclear why Geminin levels don't correlate with both FUCCI pseudotime and CC position (should be very strong correlation with Geminin and less with Cdt1).

*Response: FUCCI time from Mahdessian et al. (2021) lies in [0, 1]. However, we differ in the choice of origin (which is arbitrary) so FUCCI time of 0 matches with our cell cycle position time of 0.9m. To make a visualization where we get close to a y = x line we have made the x-axis (our cell cycle position) go from [0.9m, 0.9m + 2m] = [0.9m, 0.9m]. Basically, we are making the points monotonically increasing. The points in the box on Figure 5b have been moved by an adjustment factor, which is explained in the Results. This is basically acknowledging that at this specific time point, FUCCI has greater resolution than tricycle. We have made this adjustment in Figure 5b and not for example in Figure 5c and elsewhere. Note that Figure 5c shows (without the adjustment factor) that we do better then FUCCI at explaining gene expression dynamics over the entire cell cy¬cle (this is because we do better at the parts of the cell cycle away from this specific time point). We now also supplement the comparison of FUCCI pseudotime and our cell cycle position by using a circular correlation coefficient (which respects that the data lies on a circle). We do not use the box adjustment when we compute p which has a value of 0.7 (granted, while p has a similar range to a normal correlation, it is a different quantity and it takes a while to get a feel for how good or bad 0.7 is). To make a comparison to FUCCI pseudotime we multiple by m to get values in [0, 2m]. We edited the caption of Figure 5 to explain this more clearly. It now reads (we only quote the relevant parts of the caption; panels a-c)*

*“ Evaluation of tricycle on FUCCI datasets. (a-c) Data from Mahdessian et al. (2021). (a) FUCCI scores colored by tricycle cell cycle position. (b) Comparison between FUCCI pseudo-time and tricycle cell cycle position with a periodic loess line. We are displaying the data on [0.9m, 0.9m + 2m] (compared to [0, 2m] elsewhere) because FUCCI pseudotime of 0 roughly corre¬sponds to a tricycle position of 0.9m. Cells in the dotted rectangle were moved for display purposes in this panel by adding 2m (one period) to tricycle 0 to reflect the higher temporal resolution around the anaphase-metaphase transition for FUCCI pseudotime (see Results). (c) R2 values of periodic loess line of all projection genes when using tricycle 0 and FUCCI pseudotime as the predictor. The dashed line represents y = x.”*

*NOTE: Here, there may be some confusion about why we are talking about 0.9m vs. the reviewer’s comment about 0.8m. This is because during the revision of our paper, the au¬thors of Mahdessian et al. (2021) alerted us to the fact that they have updated their FUCCI time normalization. We have changed our FUCCI time to use the improved normalization and while the impact is relatively minimal, it used to be that FUCCI time 0 corresponds to tricycle time 0.8m but now FUCCI time 0 corresponds to tricycle time 0.9m.*

*The last part of the comment asks about why the Geminin level does not correlate with FUCCI pseudotime (which has nothing to do with tricycle and is derived using protein levels, not gene expression) and cell cycle position. Note that FUCCI pseudotime is based on the protein measurements of the gene, and we display the expression level. This result is striking evidence that the protein level of Geminin is regulated post-transcriptionally.*

*We have edited the caption of Supplemental Figure S12 to more clearly describe this. It now says*

*“ All data is from the hu2OS dataset. (a,b) The gene expression of (a) GMNN and (b) CDT1 as a function of FUCCI pseudotime derived using imaging of the protein levels. (c,d) Like (a,b) but as a function of tricycle cell cycle position inferred using scRNA data. Note that GMNN has constant expression over the cell cycle while CDT1 oscillates. This strongly suggests that the protein level of GMNN is regulated post transcriptionally.””*

- The authors should demonstrate how Tricycle's works when using available scRNA-seq datasets with known ground truth as reference, and projecting to their in-house datasets and other public datasets (with ground-truth) across different cell types. This comparison would also indicate where the tricycle-inferred position on cell cycle pseudotime (0-2) is consistent across different cell types (i.e. S/G2/M ranges from 1.5 - 0.25), or whether varies for different cell types (different cycling speeds and/or time across each phase).

*Response: We thank the reviewer for the suggestion. This concern reflects questions raised in concerns 1 and 6 and are partly addressed in our responses to those concerns. We will briefly summarize them. We prefer to use gold-standard datasets to evaluate our predictions by comparing predicted cell cycle positions to gold-standard measurements. In contrast using a gold-standard dataset to construct the reference embedding does not give us this kind of evaluation. Furthermore, we also believe it is better to use a reference dataset with many cells and the available gold standard datasets are all of the variety ”few cells, high information content per cell”.*

*We now address the second part of the concern (”This comparison would also indicate ...”). First, all datasets we have ever analyzed results in cell cycle position estimates us¬ing the full [0, 2m] interval. We believe the reviewer’s question is inspired by Figure 5 where we plot cell cycle position from [0.9m, 0.9m + 2m] which is not a smaller range; it is just shifted for display purpose. Note that tricycle estimates a circular variable so the offset can change as long as we use a full period. Second, as the reviewer points out, we have some small discrepancies in the precise locations of the various expression peaks, but they largely coincide in the sense that for some datasets, the G0/G1 peak is around 0 and sometimes it may be around 0.05 or 1.95m. This happens when we make compar¬isons between datasets with different technologies. We have added new language to the Discussion describing this and cautioning about comparing the precise peak locations be¬tween different technologies. Thirdly, it is still an open problem to obtain cell cycle length estimates from tricycle (as opposed to angles). We are actively working on this including experimentally changing cell cycle length in a fixed cell population.*

- (Minor) The size and resolutions of several main and supplementary figures was very poor (even for visualization on screen). For several figures showing multiple colors over cell cycle continuum, the stages were barely discernable, and often the legends, highlighted names were barely readable. The authors could either provide original resolution figures (separately) instead embedding within text or make figures larger in panels.

*Response: We submitted a full resolution PDF to Genome Biology, but this PDF is con¬verted into PDF (!) during the submission process and this conversion has led to un¬acceptable degration in image resolution for some (but strangely not all) images. We are not sure we can bypass the PDF conversion by the manuscript submission system, but we will update the bioRxiv version as well; the version on bioRxiv has been high resolution throughout.*

- (Minor) Fig. S30, typo in title (mNeurosphere).

*Response: The title now reads as*

*“ Tolerance of removing genes from the mNeurosphere reference”.*

**Second round of review**

**Reviewer 2**

Review of Zheng et al The revised paper is much improved, and the authors have taken my comments (and, I believe, those of other reviewers) seriously. I have only a few remaining comments to be addressed.

P3: "For this result to hold...." I think this paragraph needs improving. It is unclear what "this result" refers to, and the meaning of "at least two peak locations are required" is also unclear (it could be read as meaning that each gene must have two peaks, but in fact under the model *every* gene has one peak -- and one trough -- and the claim seems to be that these must not occur in the same place for every gene). And is it really "required"? (It seems that a constructive proof cannot show that it is "required", only that it is sufficient?) In fact stating the result this way ("at least two peak locations are required") suggests that this is a non-trivial requirement; I would suggest a phrasing along the lines of "This result holds unless all genes peak and trough in exactly the same places" would better emphasise the fact that the result will hold except in pathological cases.

p3/4 "Using the negative binomial distrbuted data required more than 2 distinct peaks to be stable". I was suprised by this so I dug a bit into the simulations for Supp Fig S1. I believe that the result obtained here is not due to a difference between negative binomial vs Gaussian models, but the fact that the negative binomial simulations *normalize the expression of each gene relative to the total expression*. If there are only two distinct patterns in the data (say G1 and G2) then this normalization effectively imposes a constraint G1+ G2 = const, creating a linear dependence between the two patterns. As a result the means lie in a 1d subspace which yields a 1-d pattern as in Supp Fig S1. (To be clear, I'm not saying the simulations are unreasonable; rather I am saying the explanation of this result as being due to the negative binomial sampling is misleading -- you would likely see something similar with Gaussian errors). [In my investigations of these simulations I found what looks like a bug in the code, detailed below, but I don't think it affects the qualitative conclusions.]

p3-6 While I agree that the explicit analysis of a very simple model (here that mean expression of each gene is a periodic function of the form A cos(theta-L)) is pedagogically helpful, some of the later references to "models" and "theory" give the impression -- misleading in my opinion -- that tricycle is based on this simple model. eg p4 "we next assess our model"; p5 "as suggested by our model..." and p6 "A clustering analysis... fits the theoretical requirements of our model", and ... "as predicted by our mathematical model".

p5: "We center the coordinate plane on (0,0) whose location corrsponds to cells with 0 expression for all 500 variable cell cycle genes". This claim seems unlikely to me, due to the fact that i) you are transforming expression using log2 with pseduo-count; ii) (I think) you are subtractacting mean (transformed) expression before performing PCA? I do think it would be useful to discuss the interpretation of (0,0)...

p7: "Because our reference embedding... well-understood mathematical properties" This sentence makes no sense to me and I suggest removing. (This paragraph would probably benefit from further shortening as it seems rather repetitive.) On loess vs trendfilter, I thank the authors for looking at this. In my opinion the trendfilter results shown here are preferable - I agree there are places where they could be smoother, but the eye easily adjusts for this. In comparison some of the oversmoothed loess plots just seem to make every thing look the same even when the data look pretty different... (eg Fig 4b). Ultimately though I leave this to the authors.

typos: p1: "and integral" p5: "relate theta to the measured..." should this be "estimated theta"? p13: "to similarity" p13: wrt p13: "conformed"

code: there is a bug in simul_negbin (eg in the file sfig.nbsimulationLibrary.R and others). Specifically I think the term lambda / colSums(lambda) in lambda_scaled <- sweep(lambda / colSums(lambda), MARGIN = 1, STATS = libsizes, FUN = "*") does not make sense (R processes matrix elements by row first, and dividing the rows of a matrix by its column sums will hardly ever make sense.) I believe it should be lambda_scaled <- sweep(t(t(lambda) / colSums(lambda)), MARGIN = 1, STATS = libsizes, FUN = "*") although you might want to check (the sweep function is a pretty obscure way to do this normalization in my opinion...)

More generally other aspects of the code don't look quite right to me, although i don't think it affects results. For example, take the line libsizes <- rep(libsize, nGenes). It doesn't make sense to repeat library size by number of genes, it should be number of cells (which here is length(timepoints))? And timepoints should really be passed in as a variable... As I said, I don't think it affects results here, but if you use this function in future work it might be worth fixing.

**Reviewer 3**

In this second revision by Zheng et al, describing ‘tricycle’ i.e. an R/Bioconductor package to infer cell cycle from scRNA-seq datasets; the authors have improved the overall flow, readability of the manuscript, as well as addressing some of the stated concerns (incl. clarification on Schwabe vs tricycle classification). A few key points and suggestions are listed below, which the authors should highlight and address.

- The authors report ‘internal controls’ (i.e. expression of some cell cycle genes and total UMIs) as a measure of continuous ordering and tricycle inference. However what ‘internal controls’ measure and show (in context of tricycle inference) is still unclear. For individual cell types, the number of UMIs should scale over cell cycle for a (i.e DNA content and replication alongside its coupled transcriptomic, proteomic turnover). But for heterogeneous multi-cell-type sample with variable cell size/amounts, it is unclear how different cells (say M/G1 or G1S) are placed in the tricycle inferred continuum.

- Regarding gold standard (FUCCI protein markers), the authors state that FACS gating of FUCCI markers is less conclusive than imaging based analysis. This is not particularly true (FACS provides a quantitative measurement of same protein fluorescence without any addition steps including antibody conjugation etc.,)

- Secondly, many scRNA-seq studies utilise index sorting of single-cells either with DNA content (DAPI/PI, Hoechst etc.,) or cell cycle markers; instead of sorting on broad stages (e.g. Quartz-seq2). These authors could also compare the tricycle inference with respective index sorted markers. - While tricycle provides an continuum/ordering of cells over 0-2 that relates to cell cycle stages; the discreet cell cycle inference of individual cells becomes tricky and challenging (especially for multi-cell type datasets). This is particularly use for when researchers are interested in cell cycle stage classification and interpretability of the inference. The authors could provide, highlight the demarcation of each cell cycle stage (with error/variance). Additionally, the authors state that peak shifts across datasets could be due batch effects (platforms etc.,); yet this affects the interpretation of inference. Particularly for cells at the transition (incl. Fig 2a: yellow G2M cells after S but also after purple MG1 cells). For example in Fig 2f, the S-phase peaks at 0.75, G2 ends at 1.5 and M-G1 is from 1.5-2. The figure (comment 2 of reviewer 3) shows G1 (0-0.3), S (0.3-), G2M (1.75). The authors can clarify if this isn’t the case; or help reconcile variation between different runs of tricycle on different datasets.

- The concern about which cell cycle stage classification to trust (between tricycle and other tools) is not really resolved. If different methods provides a slightly different stage (discrepancy), which method should be used/trusted. The authors may not be able to address this without their own gold-standard data, inference and comparison; the authors should certainly highlight this in discussion.

- It’s unclear why the initial inference on public data, followed by transferred projections to authors dataset (mNeurosphere, mHippNPC) was not shown. The public smart-seq2 datasets have more detected genes, increase quantification and estimation (with lower throughput). The author can clarify whether this out-or underperforms to current workflow (for reviewers alone).

**Authors Response**

**Point-by-point responses to the reviewers’ comments:**

Reviewer 2

“The revised paper is much improved, and the authors have taken my com¬ments (and, I believe, those of other reviewers) seriously. I have only a few remaining comments to be addressed.”

*Response: We sincerely thank the reviewer for carefully reading our manuscript. We have made changes to improve our manuscript based on constructive feedback from the reviewer.*

“P3: ”For this result to hold....” I think this paragraph needs improving. It is unclear what ”this result” refers to, and the meaning of ”at least two peak locations are required” is also unclear (it could be read as meaning that each gene must have two peaks, but in fact under the model *every* gene has one peak – and one trough – and the claim seems to be that these must not occur in the same place for every gene). And is it really ”required”? (It seems that a constructive proof cannot show that it is ”required”, only that it is sufficient?)

In fact stating the result this way (”at least two peak locations are required”) suggests that this is a non-trivial requirement; I would suggest a phrasing along the lines of ”This result holds unless all genes peak and trough in exactly the same places” would better emphasise the fact that the result will hold except in pathological cases.”

*Response: We agree with the reviewer that the current writing suggests something nontrivial where the opposite is true. In the figure below, we are showing what happens if all genes have the same peak location, but different heights, and it is clear that we do not get an ellipsoid in the first 2 PCs.*

*However – as noted by the reviewer – all of this is unimportant to data analysis and the cell cycle. We have therefore removed the sentence from Results and we are now stating the following Methods:*

*“ We are assuming at least two peak locations not separated by π (ie. two different dg’s).”*

“p3/4 ”Using the negative binomial distrbuted data required more than 2 dis¬tinct peaks to be stable”. I was suprised by this so I dug a bit into the simulations for Supp Fig S1. I believe that the result obtained here is not due to a difference between negative binomial vs Gaussian models, but the fact that the negative binomial simulations normalize the expression of each gene relative to the total expression. If there are only two distinct patterns in the data (say G1 and G2) then this normalization effectively imposes a constraint G1+ G2 = const, creating a linear dependence between the two patterns. As a result the means lie in a 1d subspace which yields a 1-d pattern as in Supp Fig S1. (To be clear, I’m not saying the simulations are unreasonable; rather I am saying the explanation of this result as being due to the negative binomial sampling is misleading – you would likely see something similar with Gaussian errors). [In my investigations of these simulations I found what looks like a bug in the code, detailed below, but I don’t think it affects the qualitative conclusions.]”

*Figure showing the necessity of two peak locations across genes. (a-d) Simulations are based on cosine functions with Gaussian noise with 2 distinct peak locations across gene, as in Figure 1 of the manuscript. (a) Expression vs. time for 2 genes with different peak locations and amplitudes. Each of the two gene peaks are replicated 50 times for a total of 500 genes and 1,000 time points (cells). (b) Principal component analysis of the data. (c) PC1 vs. the true timepoint. (d) PC2 vs. the true timepoint. (e-h) Similar to (a-d), but now all genes share the same location of peak and trough. (e) Expression vs. time for 2 genes with different amplitudes and the same peak locations.*

*Response: The reviewer is correct here and we are grateful for being corrected. We have confirmed this by doing a small experiment where we don’t impose the total counts con¬straints. Following this insight, we have changed the text in our manuscript to read: “ In addition to using the negative binomial distribution, we also mimic the library size normaliza¬tion step usually performed in scRNA-seq data analysis, which imposes a constraint on the total counts. With the total counts constraint, we need at least 3 distinct peak locations to be stable (Fig. S1, S2).”*

“p3-6 While I agree that the explicit analysis of a very simple model (here that mean expression of each gene is a periodic function of the form A cos(theta-L)) is pedagogically helpful, some of the later references to ”models” and ”theory” give the impression – misleading in my opinion – that tricycle is based on this simple model. eg p4 ”we next assess our model”; p5 ”as suggested by our model...” and p6 ”A clustering analysis... fits the theoretical requirements of our model”, and ... ”as predicted by our mathematical model”.”

*Response: We appreciate these comments which highlight a problem in our terminology. We agree that tricycle itself does not really incorporate the cosine model we present. Instead we view the model as explanation for why we observe an ellipsoid in the PCs. To clarify this, we have changed the language as follows*

*·	p4: “ We next show that PCA of cell cycle genes form an ellipsoid as predicted by the cosine model presented in the previous section, and learn an embedding representing cell cycle.”*

*·	p5: “ The first two principal components form an ellipsoid with a sparse/empty interior (Figure 2a); this reflects the behavior of our cosine model.”*

*·	p6: “ Thus, the observed behavior of the cell cycle genes in these data sets satisfy the as-sumptions of the cosine model.”*

*·	p6: “ In summary, principal component analysis of the cell cycle genes predicts cell cycle progression for the mNeurosphere and mHippNPC datasets with a high degree of similarity between the cell cycle position inferred independently in the two datasets. This observed behavior aligns with the results from the cosine model.”*

“p5: ”We center the coordinate plane on (0,0) whose location corresponds to cells with 0 expression for all 500 variable cell cycle genes”. This claim seems unlikely to me, due to the fact that i) you are transforming expression us¬ing log2 with pseduo-count; ii) (I think) you are subtractacting mean (trans¬formed) expression before performing PCA? I do think it would be useful to discuss the interpretation of (0,0)...”

*Response: Thank you for spotting this mistake. We now write*

*“ We center the coordinate plane on (0, 0). Because we mean-center the expression data prior to performing PCA, this location corresponds to having mean (across the dataset) expression for all 500 variable cell cycle genes. Linking to our idealized example with periodic functions, this will correspond to a timepoint (cell) where all genes have 0 expression after mean centering.”*

“p7: ”Because our reference embedding... well-understood mathematical prop¬erties” This sentence makes no sense to me and I suggest removing. (This paragraph would probably benefit from further shortening as it seems rather repetitive.)”

*Response: What we are trying to articulate with this sentence is the fact that we “know” how to project*

*new data into the space defined by PCA(neurosphere/reference data set). This may seem trivial to the reviewer, but we draw attention to the fact that many ”low-dimensional representations” popular in scRNA-seq analysis do not have this property. An example is UMAP, where it is not clear how to project new data into the UMAP em¬bedding. The authors of UMAP have proposed a solution to this (a solution which btw. is different from the solution used in RNA velocity), but their solution is not a standard orthogonal projection operator that we know and love from linear algebra. In contrast,* *because we use standard linear subspaces, we know how to do this.*

*However, as noted by the reviewer, this entire paragraph repeats a lot from our first overview section. We have substantially shorten it (there is still some repetition, but we believe we need to provide a short overview when we introduce tricycle).*

*The new paragraph reads“ To overcome the challenges of inferring cell cycle position in arbi-trary datasets, we propose a simple, yet highly effective transfer learning approach we term tricycle (transferable representation and inference of cell cycle). In short, we first construct a reference em-bedding representing the cell cycle process using a fixed dataset where cell cycle is the primary source of transcriptional variation. For the remainder of this manuscript we will use the cortical neurosphere data as this reference, and we claim this is a useful reference regardless of dataset. We project new data into this embedding and infer cell cycle position for each cell by the polar angle around the origin.”*

“On loess vs trendfilter, I thank the authors for looking at this. In my opinion the trendfilter results shown here are preferable - I agree there are places where they could be smoother, but the eye easily adjusts for this. In comparison some of the oversmoothed loess plots just seem to make every thing look the same even when the data look pretty different... (eg Fig 4b). Ultimately though I leave this to the authors.”

*Response: This is a combination of the smoother and the visualization of the smoothed function together*

*with the data. We think there is still room to improve the visualization, and we do not think the loess approach we use is the best. Considering the much longer computation time of trendfilter, we decide to stick with loess. We feel it is good enough for our purpose.*

“typos:

p1: ”and integral”

p5: ”relate theta to the measured...” should this be ”estimated theta”?

p13: ”to similarity”

p13: wrt

p13: ”conformed””

*Response: We have corrected these typos. The text now reads*

*·	p1: “ Cell cycle is a highly conserved mechanism with an integral role in ...”*

*·	p5: “ Next, we directly relate estimated θ to the measured transcription values.”*

*·	p13: “ ... and used the circular correlation coefficient to quantify the similarity...”*

*·	p13: “ Next, we examined the stability of tricycle regarding the choice of reference embed-ding.”*

*·	p13: “ We also confirmed the direction of ...”*

“code: there is a bug in simul negbin (eg in the file sfig.nbsimulationLibrary.R and others). Specifically I think the term

lambda / colSums(lambda)

in

lambda_ scaled <- sweep(lambda / colSums(lambda),

MARGIN = 1, STATS = libsizes, FUN = "*")

does not make sense (R processes matrix elements by row first, and dividing the rows of a matrix by its column sums will hardly ever make sense.)

I believe it should be

lambda_scaled <- sweep(t(t(lambda) / colSums(lambda)), MARGIN = 1, STATS = libsizes, FUN = "*")

although you might want to check (the sweep function is a pretty obscure way to do this normalization in my opinion...)

More generally other aspects of the code don’t look quite right to me, although i don’t think it affects results. For example, take the line libsizes

<- rep(libsize, nGenes). It doesn’t make sense to repeat library size by number of genes, it should be number of cells (which here is length(timepoints))?

And timepoints should really be passed in as a variable... As I said, I don’t think it affects results here, but if you use this function in future work it might be worth fixing.”

*Response:*

*The reviewer is totally right about the issues in simul negbin function. We have now fixed the bug and libsizes operations in simul negbin function, and updated 3 as¬sociated Fig. S1-S3. The code has been changed to*

*lambda_scaled <- sweep(lambda, MARGIN = 2,*

*STATS = colSums(lambda) / libsize, FUN = "/")*

*The associated results remain the same with the corrected Fig. S1-S3.*

Reviewer 3

“In this second revision by Zheng et al, describing ‘tricycle’ i.e. an R/Bioconductor package to infer cell cycle from scRNA-seq datasets; the authors have im¬proved the overall flow, readability of the manuscript, as well as addressing some of the stated concerns (incl. clarification on Schwabe vs tricycle classification). A few key points and suggestions are listed below, which the authors should highlight and address.”

*Response: Thank you.*

“- The authors report ‘internal controls’ (i.e. expression of some cell cycle genes and total UMIs) as a measure of continuous ordering and tricycle inference. However what ‘internal controls’ measure and show (in context of tri¬cycle inference) is still unclear. For individual cell types, the number of UMIs should scale over cell cycle for a (i.e DNA content and replication alongside its coupled transcriptomic, proteomic turnover). But for heterogeneous multi-cell-type sample with variable cell size/amounts, it is unclear how different cells (say M/G1 or G1S) are placed in the tricycle inferred continuum.”

*Response: The reviewer is concerned about the usability of tricycle on complex system with multiple cell types. The mPancreas data that we analyze in depth in our manuscript is an example of such a dataset. To help with the reviewer’s concern, we have created a new figure S12 (also depicted below) which shows the details of tricycle for each cell type. As we have emphasized in our manuscript, ductal and Ngn3 low EP cells are mostly cycling and the other cell types are mostly (but not exclusively) non-cycling.*

“- Regarding gold standard (FUCCI protein markers), the authors state that FACS gating of FUCCI markers is less conclusive than imaging based analy¬sis. This is not particularly true (FACS provides a quantitative measurement of same protein fluorescence without any addition steps including antibody conjugation etc.,)

- Secondly, many scRNA-seq studies utilise index sorting of single-cells either with DNA content (DAPI/PI, Hoechst etc.,) or cell cycle markers; instead of sorting on broad stages (e.g. Quartz-seq2). These authors could also compare the tricycle inference with respective index sorted markers.”

*Response: We did not intend to say that the experimental cell separation by FACS is any less good than the separation by FUCCI. However, there are two advantages to FUCCI (one which is relevant to our data and one which is not). (1) We get a protein measurement (by imaging) attached to individual cells, whereas in FACS we only know that the population of cells have protein measurements as determined by the gating. This is relates to cell cycle stages; the discreet cell cycle inference of individual cells becomes tricky and challenging (especially for multi-cell type datasets). This is particularly use for when researchers are interested in cell cycle stage classification and interpretability of the inference. The authors could provide, highlight the demarcation of each cell cycle stage (with error/variance).*

*“ Fig. S12. A pre-learned rotation matrix learned from proliferating cortical neurospheres enables cell cycle position estimation in the mouse developing pancreas data. (a) The mouse developing pancreas data projected into the cell cycle embedding defined by the cortical neurosphere dataset. (b-i) As (a), but we only highlight one cell type in each panel, with the rest cells as background grey points. Note that the multi potency is decreasing from the ductal cells to more differentiated secretory cell types, which are not cycling.”*

Additionally, the authors state that peak shifts across datasets could be due to batch effects (platforms etc.,); yet this affects the interpretation of inference. Particularly for cells at the transition (incl. Fig 2a: yellow G2M cells after S but also after purple MG1 cells). For example in Fig 2f, the S-phase peaks at 0.75ir, G2 ends at 1.5ir and M-G1 is from 1.5ir-2ir. The figure (comment 2 of reviewer 3) shows G1 (0-0.3ir), S (0.3-ir), G2M (1.75ir). The authors can clarify if this isn’t the case; or help reconcile variation between different runs of tricycle on different datasets.”

*Response: The reviewer is asking about the discrepancy across different datasets and to illustrate this they draw attention to Figure 2a, 2f as well as Figure “comment 2 of reviewer 3” (made for the last round of revision). Here we note that these figures all represent the same dataset, although this dataset is visualized in different ways (cell cycle embedding for Figure 2a, UMAP for Figure “comment 2 of reviewer 3”). Since tricycle positions are deterministic, ie. the tricycle assignment is the exact same in these figures. Perhaps the reviewer is confused by the colors in these figures, which are either from the SchwabeCC stage predictor (Figure 2a) or Seurat (Figure “comment 2 of reviewer 3”), but that is a discrepancy between these two other methods (which do not agree).*

*To help readers relate our continuous cell cycle “time” to the discrete stage, we have now put the discrete stage labels in the circular legends in Figure 4-6. But we emphasize that the positions are approximated based on peak expression patterns of mNeurosphere dataset in Fig S7, and we are not trying to discretize the assignments.*

“- The concern about which cell cycle stage classification to trust (between tricycle and other tools) is not really resolved. If different methods provides a slightly different stage (discrepancy), which method should be used/trusted. The authors may not be able to address this without their own gold-standard data, inference and comparison; the authors should certainly highlight this in discussion.”

*Response: The main advantage of tricycle is two-fold. First, as a design principle for tricycle, we provide an estimate of continuous cell cycle “time” and do not attempt to force a discrete classification of cell cycle stage on the cells. We believe that this approach can have significant advantages over discrete classification methods, however, we make these comparisons as these are the only available methods against which to compare. Whether the use of a continuous estimate of cell cycle position is an advantage over a discrete stage assignment depends on the user’s needs. We do not dispute the statement*

*that some users may only care about coarse stage assignments.*

*Second, tricycle has an unusual degree of robustness. It appears to yield reasonable estimates across all datasets we use (roughly assessed using internal controls), something we have shown is not true for any other method with the possible exception of Seurat’s 3 stage assignment.*

*For a continuous cell cycle “time”, we show that tricycle is comparable in performance to external, gold-standard measurements. We believe that to be a substantial accomplish¬ment. And this is happening without any dataset dependent optimization.*

*In light of this (including the applicability to datasets with multiple cell types) – which is critical for data analysis, and which is much harder to assess for discrete stage assignments – we have no reservations about recommending tricycle for any application.*

*Essentially all of our method comparisons are in the supplement. Here we show that all the continuous time estimators have some issues with robustness. We also show that – for some datasets – some of the discrete stage assignments are inferior to tricycle, such as the SchwabeCC predictions having a high degree of mislabelled G0/G1 cells and cyclone having issues with correctly delimiting S phase. Of the discrete cell cycle assignments, Seurat appears to be the most robust method. The method comparisons (which are de¬scribed in detail in the supplement) are summarized in the Results section “Comparison with existing tools for cell cycle position inference”, and we currently believe it would be too repetitive to repeat these conclusions in the Discussion.*

“- It’s unclear why the initial inference on public data, followed by transferred projections to authors dataset (mNeurosphere, mHippNPC) was not shown. The public smart-seq2 datasets have more detected genes, increase quantifica¬tion and estimation (with lower throughput). The author can clarify whether this out-or underperforms to current workflow (for reviewers alone).”

*Response: Throughout our manuscript we have used the mNeurosphere dataset as a reference and we claim that this results in excellent cell cycle positions for all datasets we have used. This claim is strongly supported by our analyses, including performance on external gold standard datasets.*

*However, one could certainly imagine using different reference data. Because we start with an unsupervised learning (estimation of the embedding), there are some requirements for the learning process to work. We want the major source of variation to be cell cycle and we want minimal technical variation. One advantage of the mNeurosphere dataset is that it has two replicates and we integrate these two replicates using Seurat (CCA). This effectively removes variation which is not shared between the replicates, which has a substantial positive impact on the unsupervised learning process (not shown in the manuscript). However, because the learning process is unsupervised, we do not use any cell cycle time labels; ie. we don’t see what benefit there would be to use the gold-standard datasets.*

*In fact, we don’t think that the gold standard datasets are better for constructing the reference embedding (for example, the best FUCCI dataset from Mahdessian et al does not have any replicates), although we acknowledge they have higher information content per cell.*

*In summary, we claim the mNeurosphere data is suitable for the job and we used gold-standard datasets to support this claim.*
